# Introducing a Comprehensive Framework for Competency-based Procedure Training

**DOI:** 10.1007/s11606-025-09677-2

**Published:** 2025-07-08

**Authors:** Pete Meliagros, Rebecca Forrest

**Affiliations:** 1 Department of Internal Medicine, Hospital Medicine, VCU Health, West Hospital, 6Th Floor, East Wing 206, 1200 East Broad Street, P.O. Box 980509, Richmond, VA 23298-0509 USA; 2 Internal Medicine Training Program, Department of Internal Medicine, Hospital Medicine, Virginia Commonwealth University School of Medicine/VCU Health, Richmond, VA USA

**Keywords:** Curriculum development, Procedure competency, Procedure training and assessment

## Abstract

**Background:**

Select graduating residents across various specialties will need to perform bedside procedures as fellows, as supervising faculty, or as practicing providers. Therefore, the development of a focused and structured curriculum is needed to enhance procedure training.

**Aim:**

We aim to introduce reproducible frameworks and tools for procedure training.

**Setting:**

Virginia Commonwealth University internal medicine residents rotate through a two-tertiary care hospital system which consists of 1200 inpatient beds.

**Participants:**

Categorical internal medicine residents participated in this curriculum with no significant difference in age, gender, or percentage of residents pursuing procedure-related fields before or after curriculum implementation.

**Program Description:**

The procedure curriculum entails just-in-time simulation-based mastery learning (SBML) for bedside procedures with complete supervision in a clinical environment thereafter with multimodal assessments regardless of competency level achieved at the discretion of a procedure competency committee.

**Program Evaluation:**

Residents provide feedback via dedicated evaluations for faculty, SBML sessions, procedure rotation, and program evaluations.

**Discussion:**

Our findings reinforce the use of just-in-time SBML and a medicine procedure service and show the benefit of continued supervision with multimodal assessments regardless of competency level assigned by a procedure competency committee.

**Supplementary Information:**

The online version contains supplementary material available at 10.1007/s11606-025-09677-2.

## BACKGROUND

The American Board of Internal Medicine (ABIM) and Accreditation Council for Graduate Medical Education (ACGME) do not provide standards for training and assessing learners in bedside procedures and program directors report a wide variety of approaches.^1–3^ Holmboe et al. describe competency-based medical education as an outcomes approach to and philosophy in designing the explicit developmental progression of health professionals to meet the needs of those they serve.^4^ Among its fundamental characteristics is the shift in emphasis from time-based programs based solely on exposure to experiences in favor of an emphasis on progressively sequenced needs-based graduate outcomes and learner centeredness.^4–6^ This approach is well known and used for ACGME milestones,^2^ but has yet to translate to procedural education.

Barsuk et al. described the benefits of simulation-based mastery learning (SBML) on resident education and patient safety.^7^ Lenchus et al. showed the added benefit of enhanced supervision by a medicine procedure service (MPS) combined with SBML.^8^

More recently McGaghie et al. described “powerful medical education” which uses new technologies informed by the science and expertise of stakeholders that value evidence-based education and support innovation.^9^ Extended reality also has some potential for procedural training which may aid resource-poor areas.^10^ Despite refining and standardizing teaching practices, assessment of procedural competency is not robust.

The complex nature of competencies requires an organized multifaceted approach to assessing different types of learners. Miller’s pyramid (knows, knows how, shows how, does) begins to provide a framework.^11,12^ Hubert and Stuart Dreyfus describe characteristics of learners and steps to acquire competence.^13^ Criticism of these models includes the difficulty assessing stages and fluidity of competency with limited tools provided to reliably assess competence at higher levels.^14–17^

Van Der Vleuten suggests these variables guide the selection of appropriate assessment tools: validity, reliability, educational impact, cost effectiveness, and acceptability.^18^ As the ability of the assessor limits the quality of assessment tools, faculty development is key to successful evaluations.^19^

In global assessment, if a group of individuals utilizes effective practices and a shared mental model, they make better decisions about learners and better support professional development.^20^ We previously reported the feasibility and benefits of implementation of a procedure competency committee (PCC).^21^

**Figure 1:**
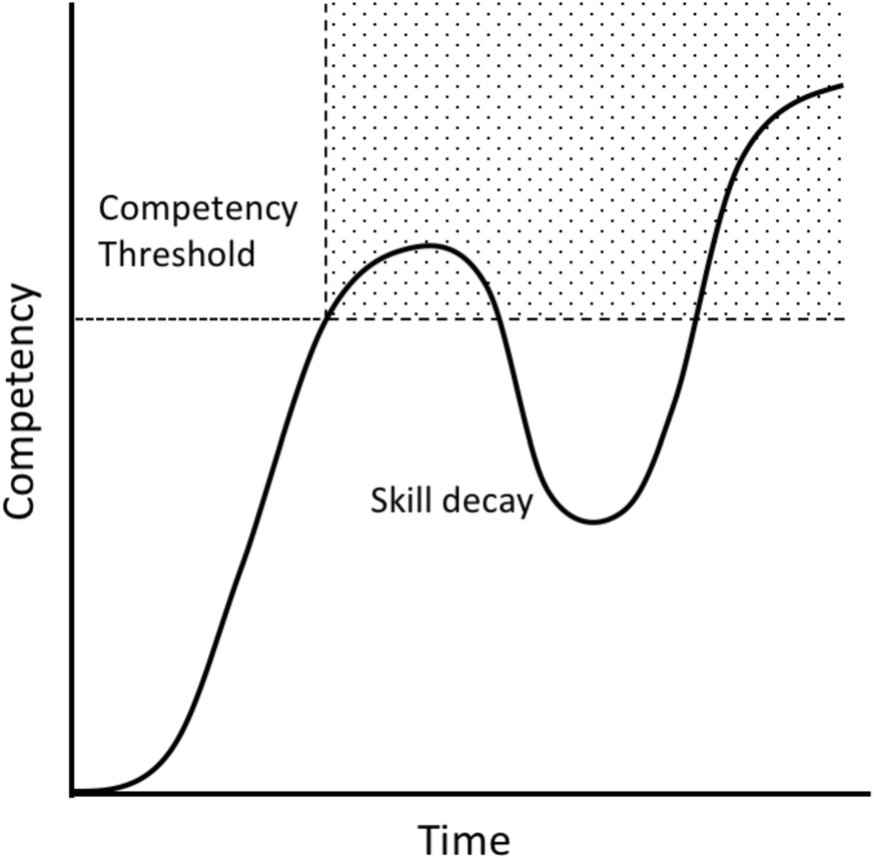
Learning curve showing common arbitrary line utilized for competency threshold for independence. Current models are not addressing skill decay nor advancement into higher levels of competency.

One institution ambitiously standardized training across the institution utilizing technology to provide optimal transparency of learners’ competency but still relies on minimum numbers to determine competency.^22^ Figure 1 shows how this common threshold ignores skill decay and does not provide opportunities for growth to higher competency.

## AIM

We aim to introduce tools and reproducible frameworks that incorporate some of these educational concepts to address the current shortcomings in procedure education.

## SETTING AND PARTICIPANTS

VCU categorical internal medicine residents rotate through a two-hospital system which consists of VCU Health which is a level 1 tertiary care hospital with 820 staffed inpatient beds as well as Central Virginia VA Health Care System which is a tertiary care hospital with 399 staffed inpatient beds.

The average class size was larger after the curriculum was introduced (30.33 vs 32.29, *p* < 0.01). Resident age at time of graduation (29.86 vs 30.74, *p* = 0.2), gender (43.2 vs 44.35% female, *p* = 0.47), number of international students (10.9 vs 6.4%, *p* = 0.58), and number of osteopathic students (10.8 vs 14.1%, *p* = 0.48) were not significantly different before or after curriculum implementation of longitudinal curriculum. On average, there was no significant difference in percentage of residents pursuing procedure related fields (hospital medicine, pulmonary critical care, cardiology, and gastroenterology) before or after curriculum implementation (64 vs 64.5%, *p* = 69.7).

## PROGRAM DESCRIPTION

In 2016, the program introduced a formal procedure curriculum based on Sawyer et al.’s educational framework to provide residents with fundamental knowledge and hands-on simulation training followed by clinical opportunities under appropriate supervision.^23^ Concurrently, the Department of Internal Medicine established a hospitalist-staffed MPS and required residents to perform all procedures under expert supervision. The MPS is a 2-week consultation service consisting of a hospitalist attending and 0–2 residents, staffed 7 days a week providing consults to all medical and surgical services. All PGY2 residents rotate on this service, and residents pursuing procedure-oriented careers or who require additional exposure to meet minimum program requirements rotate again during PGY3. Experience with procedures otherwise occurs during night and ICU rotations.

The curriculum encompasses informed consent, sterile technique, universal precautions, and use of ultrasound guidance for common bedside procedures: peripheral intravenous catheter placement, arterial blood gas acquisition, central venous catheter insertion and removal, arthrocentesis, arterial line insertion, paracentesis, thoracentesis, and lumbar puncture. Training uses a consistent format for each procedure and includes obligatory simulations.

SBML is a cornerstone of our training and benefits from advanced mannikins, procedure-specific equipment, ultrasound technology, and the guidance of experienced facilitators.

Utilizing Workday®, we created online learning modules with quizzes for each procedure and assigned them to residents prior to simulation sessions. These modules outline the indications, contraindications, complications, proper use of ultrasound, and procedural components. PGY1 residents have the opportunity for arterial line and central venous catheter insertion in the ICU, so they complete a vascular access SBML course prior to that rotation. PGY2 residents participate in a central venous catheter insertion, paracentesis, and lumbar puncture SBML course at the beginning of their PGY2 year over a 4-week period. They additionally spend the first day of the MPS in the simulation lab reviewing these procedures. Thoracentesis training occurs during dedicated conference time for select residents. All sessions typically run for 3–4 hours with a ratio of one to two faculty to five learners.

In pursuit of rigorous training and assessment, we adapted previously validated checklists for central line placement, paracentesis, and lumbar puncture.^5^ For the remaining procedures, a group of procedure experts in our institution crafted similar checklists using a Delphi approach.^24^ To establish a standardized benchmark for advancement in our competency spectrum, a group of experts determined a minimum passing standard for each procedure by utilizing the Mastery Angoff method.^25^ Residents must achieve these standards before pursuing key components of procedures in the clinical environment.

Residents and faculty can access supplemental materials via a program designed procedure medicine application using the Outlook platform, Power Apps®. Materials include longitudinal and rotation curriculums, training modules, checklists, videos, and relevant literature.

Residents log each procedure in the data management system New Innovations® and we encourage immediate post procedure review with their supervisor. Logs initially included checklist and global rating scale assessments and we later added an entrustment scale and procedure complexity to enhance data available to the PCC. While checklists are an effective tool particularly with early learners, global rating and entrustment scales offer better reliability and sensitivity.^26,27^

The Associate Program Director of Procedure Education is responsible for onboarding faculty who supervise procedures using identical SBML and training in assessment tools to standardize supervisor practice and assessment. All supervisors understand the tools and utilize criterion-referenced methods over normative or ordinal assessment methods. Our MPS faculty supervise the majority of procedures and are well trained and involved in SBML sessions where assessment tools are first utilized. We also focus on training residents in self-evaluations while they rotate on the MPS. We are actively working to build a process to similarly train fellows.

A team of experts in education and procedure medicine created procedure milestones.^21^ These milestones are similar to the Dreyfus model, ranging from “able to observe” to “able to conduct with indirect supervision and supervise others.”^13^ To facilitate this process, we established a PCC consisting of MPS attendings. The committee, led by the Associate Program Director for Procedure Education, assesses resident portfolios of individual loggers, rotation evaluations, and performance in simulation and on knowledge acquisition quizzes, to determine competency level for each procedure and provides semiannual feedback to residents.

Residents must achieve specific objectives in knowledge, attitude, and skills to advance to higher levels of competency. The residency program tailored the final verification of training form to include the quantity and competency level for each procedure performed.

To prepare residents pursuing procedure-oriented subspecialty fields, we developed specific goals for more advanced procedures based on career plans. We selected higher graduation requirements based on our data suggesting the number which most residents achieve a competency level of indirect supervision,^17^ and recommend residents obtain higher levels of competency in relevant procedures.

## PROGRAM EVALUATION

Initial surveys for SBML sessions showed unanimous positive feedback for three consecutive years and are no longer distributed. Residents complete procedure rotation and faculty evaluations and an annual program survey that seeks feedback on individual procedure curriculum components. The Associate Program Director of Procedure Education monitors impacts of curriculum changes on procedures logged and resident performance.

We summarized data using mean and standard deviation for continuous variables and frequencies of percentages for categorical variables. We compared pre-implementation (2013–2016) with post implementation (2016–2023) data using Student’s* t*-test. All analysis was conducted using Excel. The Institutional Review Board at our institution deemed the study exempt.

**Figure 2:**
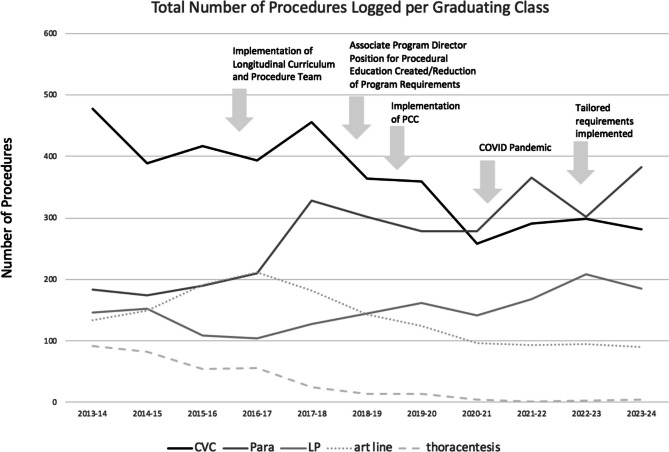
The total number of procedures logged per graduating class of AY 2013–2024 along with important curriculum milestones. Procedure requirement modifications in 2018 included the removal of graduation requirement of 3 thoracentesis and 5 arterial line insertions as well as transition from 5 femoral central line insertions and 5 internal jugular line insertions to 5 central line insertions. SBML and MPS introduced in 2016 were focused on paracentesis, lumbar puncture, and central line insertion, with later implementation of arterial line insertion and thoracentesis SBML when tailored requirements were implemented

Figure 2 shows total procedures logged by graduating residents between 2013 and 2024 highlighting curriculum changes. The number of paracenteses logged increased significantly after implementation of a MPS (182.67 vs 305.85 paracentesis, *p* < 0.001). There was a non-statistically significant increase in lumbar punctures (135 vs 155, *p* = 0.33). There was a significant decline in central line insertion (427 vs 336, *p* = 0.045). There was non-significant decrease in arterial lines logged (158 vs 129.25, *p* = 0.26). There was a significant decrease in the number of thoracentesis procedures logged (76 vs 15.5, *p* = < 0.01).

Since the implementation of the MPS, 63% of all procedures logged by residents are supervised by procedure team attendings (> 90% of paracenteses and lumbar punctures and < 20% of central venous catheterizations). The remainder of procedures are supervised by fellows, advanced practice providers, and specialty attendings.

The internal medicine procedure rotation evaluations from 2017 to 2024 show > 92% of residents having positive experiences with respect to their clinical experience, quality of education, and quality of supervision on the service (100% response rate).

All categorical residents participate in SBML and note that it is comparable but does not supplant supervised learning in the clinical environment. Only three residents required additional simulation time to meet minimum passing standards.

This PCC was established in 2019 and provides twice annual reviews to each resident regarding their progression through the procedure milestones. When asked on an annual survey in 2021 if the PCC had a meaningful impact on their education, only 32% of residents had a positive impression while 20% had a negative impression (> 60% annual response rate). There has been a gradual improvement in perception with the most recent survey in 2024 showing 50% of residents had a positive vs 6% negative impression of the PCC’s impact on their education.

## DISCUSSION

We describe the first longitudinal curriculum for procedure medicine utilizing previously described training and assessment tools in a more tailored and holistic framework. Our findings further support the use of just-in-time SBML and a MPS and show that a MPS significantly improves resident exposure to procedures. Utilizing multimodal assessments of residents along with independently developed milestones allows our unique PCC to provide substantial and comprehensive feedback to residents and transitions from determining competency solely based on numbers. A crucial component of this curriculum, which may be constrained by resources, is to have all procedures supervised by experts regardless if a resident is deemed competent. This is imperative to our curriculum as it optimizes evaluations and allows continued feedback to learners to advance further in the competency spectrum and is our main means of tracking skill decay in the clinical environment. We find that a MPS with members involved in our SBML and PCC allows for more effective standardized teaching and assessment. It is important to educate supervisors in the assessment tools, but we also see value in coaching residents in self-evaluation. This approach is likely to be beneficial in resource-limited settings where expert supervision is not readily available.

These adaptations improved data acquisition guiding curriculum improvements and providing more informative summaries to residents.^21^ Cultural changes take time, and program surveys show a gradual improvement in appreciation of curricular components.

Residents were surprisingly sensitive to changes in program requirements, seen in the decrease in number of thoracentesis and central line insertions following modification in graduation requirements. Therefore, it is important to assure transparency and communicate changes to residents while monitoring outcomes in order to respond appropriately to resident and system needs.

### Limitations

This is a single-institution innovation with a small sample size; however, our program shares commonalities with other Internal Medicine training programs, which allows for generalization. We have not evaluated patient outcomes and therefore focus changes based on survey data and resident global performance as outcome measures. The tools utilized in our study lack formal testing of validity and reliability, but we feel we have outlined how we have worked to optimize this within our limitations.

The methods tested benefit from ample resources, including a simulation center, MPS, and experienced faculty willing to invest time in teaching and competency reviews. We are able to provide year-round supervision for procedures by our fellows, intensivists, and nocturnists.

### Conclusion

Our proposed curriculum promotes methodically progressive and tailored training with robust feedback mechanisms to aid residents in their procedural education. It begins to provide a format on how to transcend between stages of competency that is currently lacking in existing educational models. We also provide comprehensive training and assessment tools which can be tailored and validated at individual institutions based on their needs. We hope that this will assist other institutions to provide a more standardized approach to better prepare learners pursuing procedure-oriented careers.

## Supplementary Information

Below is the link to the electronic supplementary material.

Supplementary file1 (DOCX 32.8 KB)

Supplementary file2 (DOCX 18.4 KB)

Supplementary file3 (DOCX 31.7 KB)

Supplementary file4 (DOCX 32.1 KB)

Supplementary file5 (DOCX 23.6 KB)

Supplementary file6 (DOCX 18.2 KB)

Supplementary file7 (DOCX 31.5 KB)

Supplementary file8 (DOCX 31.5 KB)

Supplementary file9 (DOCX 14.9 KB)

Supplementary file10 (DOCX 32.3 KB)

Supplementary file11 (DOCX 15.1 KB)

Supplementary file12 (DOC 2.08 MB)

Supplementary file13 (DOC 2.29 MB)

Supplementary file14 (PDF 1.00 MB)

Supplementary file15 (DOC 1.77 MB)

Supplementary file16 (PDF 3.45 MB)

Supplementary file17 (DOC 4.56 MB)

Supplementary file18 (DOC 2.32 MB)

Supplementary file19 (DOCX 16.3 KB)

Supplementary file20 (DOCX 17.6 KB)

Supplementary file21 (DOCX 18.2 KB)

Supplementary file22 (DOCX 17.3 KB)

Supplementary file23 (DOCX 14.6 KB)

Supplementary file24 (DOCX 16.3 KB)

Supplementary file25 (DOCX 185 KB)

Supplementary file26 (DOCX 1.37 MB)

Supplementary file27 (DOCX 40.0 KB)

## Data Availability

The data utilized in this manuscript are available upon request.
