# Supplementary material for: Introducing a Comprehensive Framework for Competency-based Procedure Training
Source: J Gen Intern Med. 2025 Jul 8;40(15):3560–5. doi: 10.1007/s11606-025-09677-2 (PMC12612326; doi:10.1007/s11606-025-09677-2)
Supplement: Supplementary file 1 — Supplementary file1 (DOCX 32.8 KB) [file 11606_2025_9677_MOESM1_ESM.docx]

**Arterial Line Placement**
Performance Checklist

| Name |  | Date |  |
| --- | --- | --- | --- |
| Training Program |  | Procedure/Site |  |
| Training Year |  | Attending |  |

| Task  (chronological Order) | | Incompletely Performed | Completely Performed | Notes  (Complete if not done at all or incompletely performed) |
| --- | --- | --- | --- | --- |
| Pre-Procedure | 1) Review patient’s chart, labs, and imaging (as relevant) |  |  |  |
|  | 2) Locate radial or femoral pulse via palpation and ultrasound |  |  |  |
|  | 3) Obtain informed consent and inform nursing |  |  |  |
|  | 4) Gather supplies: arterial line kit, set up flushed arterial line tubing, etc |  |  |  |
|  | 5) Position patient: arm abducted, wrist extended for radial approach, leg straight vs externally rotated for femoral approach |  |  |  |
|  | 6) Wash hands and don personal protective equipment (sterile gown and sterile gloves, eye protection) |  |  |  |
|  | 7) Prepare site using chlorhexidine |  |  |  |
|  | 8) Drape site using sterile technique |  |  |  |
|  | 9) “time out”: verify patient, procedure, and insertion site are correct |  |  |  |
|  | 10) Utilize local anesthetic (1 % w/o epi) |  |  |  |
|  | | | | |
| Procedure | 11) Prepare the kit: assemble the needle/catheter device |  |  |  |
|  | 12) Insert needle bevel up with dominant hand at 30-45%, stop when blood flash |  |  |  |
|  | 13) Insert wire through needle into vessel, remove needle if applicable |  |  |  |
|  | 14) Holding wire still, guide catheter over needle wire system |  |  |  |
|  | 15) Withdraw wire (and needle) and apply pressure proximate to puncture site or hold finger over catheter hub |  |  |  |
|  | 16) Place plastic coupler to pressure transducer, and assure appropriate waveform with patient level |  |  |  |
|  | 17) Secure catheter in place |  |  |  |
|  |  |  |  |  |
| Post-  Procedure | 18) Clean the area, ensure no bleeding, and apply dressing |  |  |  |
|  | 19) Throw away sharps |  |  |  |
|  | 20) Discard protective clothing |  |  |  |
|  | 21) Wash hands |  |  |  |
|  | 23) Document procedure and update nursing and primary team |  |  |  |

Number of attempts at procedure: ______
