# Supplementary material for: Introducing a Comprehensive Framework for Competency-based Procedure Training
Source: J Gen Intern Med. 2025 Jul 8;40(15):3560–5. doi: 10.1007/s11606-025-09677-2 (PMC12612326; doi:10.1007/s11606-025-09677-2)
Supplement: Supplementary file 2 — Supplementary file2 (DOCX 18.4 KB) [file 11606_2025_9677_MOESM2_ESM.docx]

**Arterial Puncture for Blood Gas**

Performance Checklist

**Name: ­­­­­ Date**:

**Proctor:**

| **Task** | | **Incompletely**  **Performed**  **(1 point)** | **Completely**  **Performed**  **(2 points)** | **Notes**  (Complete this section if learner does not complete tasks or incompletely performs) |
| --- | --- | --- | --- | --- |
| **Pre-Procedure** | 1. Lists indications and contraindications |  |  |  |
|  | 1. Performs Allen Test |  |  |  |
|  | 1. Gather supplies: arterial puncture kit (syringe, 20-25G needle, heparinized cap), antiseptic swab, sterile gauze, tape. |  |  |  |
|  | 1. Position Patient: Arm abducted, wrist extended (consider rolled towel) |  |  |  |
|  | 1. Palpate radial pulse or with identify with ultrasound (probe cover recommended) |  |  |  |
|  | 1. Wash hands and don personal protective equipment (nonsterile gloves, goggles) |  |  |  |
|  | 1. Sterilize procedure site |  |  |  |
|  | | | | |
| **Procedure** | 1. Locate radial pulse by palpation or with US while maintain sterility of procedure site |  |  |  |
|  | 1. Insert needle with dominant hand at 30-45^o^ angle – needle bevel up; stop when blood flash evident |  |  |  |
|  | 1. Allow fill of syringe to 1-2 cc |  |  |  |
|  | 1. Remove needle and hold pressure over procedure site for appropriate duration based on patient parameters |  |  |  |
|  | 1. Apply gauze and tape |  |  |  |
|  | | | | |
| **Post-procedure** | 1. Expel air from syringe; cap needle or place needle in stopper. |  |  |  |
|  | 1. Apply heparinized cap to syringe and push plunger to ensure blood encounters the heparin |  |  |  |
|  | 1. Place patient label; place in ice if sending to lab |  |  |  |
|  | 1. Discard all sharps appropriately |  |  |  |
|  | 1. Remove protective equipment; wash hands |  |  |  |
