# Supplementary material for: Introducing a Comprehensive Framework for Competency-based Procedure Training
Source: J Gen Intern Med. 2025 Jul 8;40(15):3560–5. doi: 10.1007/s11606-025-09677-2 (PMC12612326; doi:10.1007/s11606-025-09677-2)
Supplement: Supplementary file 3 — Supplementary file3 (DOCX 31.7 KB) [file 11606_2025_9677_MOESM3_ESM.docx]

**Knee Arthrocentesis**
Performance Checklist

| Name |  | Date |  |
| --- | --- | --- | --- |
| Training Program |  | Procedure/Site |  |
| Training Year |  | Attending |  |

| Task  (chronological Order) | | Incompletely Performed | Completely Performed | Notes  (Complete if not done at all or incompletely performed) |
| --- | --- | --- | --- | --- |
| Pre-Procedure | 1) Review patient’s chart, labs, and imaging (as relevant) |  |  |  |
|  | 2) Obtain informed consent |  |  |  |
|  | 3) Position patient |  |  |  |
|  | 4) Localize/mark needle insertion site by palpation or ultrasound |  |  |  |
|  | 6) Wash hands and don personal protective equipment |  |  |  |
|  | 7) Prepare site using chlorhexidine |  |  |  |
|  | 8) Drape site using sterile technique |  |  |  |
|  | 9) “time out”: verify patient, procedure, and insertion site are correct |  |  |  |
|  | 10) Utilize local anesthetic (1 % w/o epi) or topical spray |  |  |  |
|  | | | | |
| Procedure | 11) Insert needle |  |  |  |
|  | 12) Stop advancement of needle once fluid is aspirated |  |  |  |
|  | 13) Aspirate fluid |  |  |  |
|  | 14) Withdraw needle |  |  |  |
|  |  |  |  |  |
| Post-  Procedure | 18) Clean the area and apply dressing |  |  |  |
|  | 19) Throw away sharps |  |  |  |
|  | 20) Discard protective clothing |  |  |  |
|  | 21) Wash hands |  |  |  |
|  | 23) Document procedure and update nursing and primary team |  |  |  |

Number of attempts at procedure: ______
