# Supplementary material for: Introducing a Comprehensive Framework for Competency-based Procedure Training
Source: J Gen Intern Med. 2025 Jul 8;40(15):3560–5. doi: 10.1007/s11606-025-09677-2 (PMC12612326; doi:10.1007/s11606-025-09677-2)
Supplement: Supplementary file 4 — Supplementary file4 (DOCX 32.1 KB) [file 11606_2025_9677_MOESM4_ESM.docx]

**Central Venous Catheterization**
Performance Checklist

| Name |  | Date |  |
| --- | --- | --- | --- |
| Training Program |  | Procedure/Site |  |
| Training Year |  | Attending |  |

| Task  (chronological Order) | | Incompletely Performed | Completely Performed | Notes  (Complete if not done at all or incompletely performed) |
| --- | --- | --- | --- | --- |
| Pre-Procedure | 1)Review Patients’ chart, labs, and imaging (as relevant) |  |  |  |
|  | 2) Obtain informed consent: verify patient, procedure, and site |  |  |  |
|  | 3) Position patient: supine with HOB at 0 degrees or Trendelenburg for IJ or SC; HOB at up to 30 degrees for fem |  |  |  |
|  | 4) Localize/mark needle insertion site |  |  |  |
|  | 5) Put on hat and mask; wash hands with soap and water |  |  |  |
|  | 6) Don protective clothing: sterile gown and sterile gloves |  |  |  |
|  | 7) Prepare site using chlorhexidine |  |  |  |
|  | 8) Drape site using maximal barrier precautions; sheath ultrasound with probe cover |  |  |  |
|  | 9) “time out”: verify patient, procedure, and insertion site are correct |  |  |  |
|  | 10) Inject anesthetic |  |  |  |
|  |  |  |  |  |
| Procedure | 11) Prepare the kit: flush all ports of the catheter with saline |  |  |  |
|  | 12) Insert needle: track advance with real-time US guidance |  |  |  |
|  | 13) Obtain venous access (perform a. OR b. below |  |  |  |
|  | 1. Disconnect syringe; se the introducer to advance the curved end of the guide wire through the needle |  |  |  |
|  | 1. Pass the guide wire through the perforated end of the syringe plunger |  |  |  |
|  | 14) Holding the guidewire, withdraw the needle with or without the syringe |  |  |  |
|  | 15) Verify wire in vein with ultrasound. |  |  |  |
|  | 16) Thread the dilator over the guide wire; make a small superficial skin incision at the entry of the wire, and advance the dilator(s) |  |  |  |
|  | 17) Withdraw the dilator(s) and advance the catheter over the guide wire wile firmly holding the wire |  |  |  |
|  | 18) Remove the guidewire – state “wire out” |  |  |  |
|  | 19) Check for blood return in all ports. Flush the ports. Place cabs on the hubs. |  |  |  |
|  | 20) Secure the catheter in place |  |  |  |
|  | 21) clean the area, apply adhesive glue and CHG Tegaderm |  |  |  |
|  |  |  |  |  |
| Post-  Procedure | 22) Throw away sharps |  |  |  |
|  | 23) Discard protective clothing |  |  |  |
|  | 24) Wash hands |  |  |  |
|  | 25) Obtain chest x-ray if IJ or SC site accessed |  |  |  |
|  | 26) Document procedure and notify nurse and primary team |  |  |  |

Number of attempts at procedure: ______

Modified in May 2020 with permission from Joshua D. Lenchus, DO, RPh, FACP, SFHM; University of Miami – Jackson Memorial Hospital for Patient safety
