# Supplementary material for: Introducing a Comprehensive Framework for Competency-based Procedure Training
Source: J Gen Intern Med. 2025 Jul 8;40(15):3560–5. doi: 10.1007/s11606-025-09677-2 (PMC12612326; doi:10.1007/s11606-025-09677-2)
Supplement: Supplementary file 5 — Supplementary file5 (DOCX 23.6 KB) [file 11606_2025_9677_MOESM5_ESM.docx]

**Central Line Removal**
Performance Checklist

| Name |  | Date |  |
| --- | --- | --- | --- |
| Training Program |  | Procedure/Site |  |
| Training Year |  | Attending |  |

| Task  (chronological Order) | | Incompletely Performed | Completely Performed | Notes  (Complete if not done at all or incompletely performed) |
| --- | --- | --- | --- | --- |
| Pre-Procedure | 1) Review Patient’s chart, labs, and imaging (as relevant) |  |  |  |
|  | 2) Identify patient and explain to them the procedure |  |  |  |
|  | 2) Position patient: supine or preferably Trendelenburg for thorax access |  |  |  |
|  | 3) Assure alternative access has already been obtained if necessary |  |  |  |
|  | 5) Clamp and disconnect any IV that is running through CVC |  |  |  |
|  | 6) Wash hands with soap and water and put on gloves |  |  |  |
|  |  |  |  |  |
| Procedure | 11) Remove old dressing and sutures |  |  |  |
|  | 12) Instruct patient to perform Valsalva maneuver (utilize vent if necessary) |  |  |  |
|  | 13) Gently withdraw catheter while applying firm, direct pressure at insertion site using an occlusive petrolatum dressing |  |  |  |
|  | 14) Hold pressure as needed to achieve hemostasis (minimum 5 min) |  |  |  |
|  | 15) Apply occlusive dressing and pressure bandage (to remain in place for at least 24 hours) |  |  |  |
|  | 16) Instruct patient to remain supine for 30 min for IJ, subclavian, and PICC or 2 hours for femoral access |  |  |  |
|  |  |  |  |  |
| Post-  Procedure | 18) Evaluate removed catheter to assure intact |  |  |  |
|  | 19) Discard used materials |  |  |  |
|  | 21) Wash hands |  |  |  |
|  | 23) Update nursing and primary team |  |  |  |

Number of attempts at procedure: ______
