# Supplementary material for: Introducing a Comprehensive Framework for Competency-based Procedure Training
Source: J Gen Intern Med. 2025 Jul 8;40(15):3560–5. doi: 10.1007/s11606-025-09677-2 (PMC12612326; doi:10.1007/s11606-025-09677-2)
Supplement: Supplementary file 7 — Supplementary file7 (DOCX 31.5 KB) [file 11606_2025_9677_MOESM7_ESM.docx]

**Lumbar Puncture**
Performance Checklist

| Name |  | Date |  |
| --- | --- | --- | --- |
| Training Program |  | Procedure/Site |  |
| Training Year |  | Attending |  |

| Task  (chronological Order) | | Incompletely Performed | Completely Performed | Notes  (Complete if not done at all or incompletely performed) |
| --- | --- | --- | --- | --- |
| Pre-Procedure | 1)Review Patients’ chart, labs, and imaging (as relevant) |  |  |  |
|  | 2) Obtain informed consent: verify patient, procedure, and site |  |  |  |
|  | 3) Position patient: lateral recumbent preferred if desire accurate opening pressure |  |  |  |
|  | 4) Localize/mark needle insertion site: L3-L4/L4-L5/L5-S1 |  |  |  |
|  | 5) Put on hat and mask; wash hands with soap and water |  |  |  |
|  | 6) Don protective clothing: sterile gown and sterile gloves |  |  |  |
|  | 7) Prepare site using chlorhexidine |  |  |  |
|  | 8) Drape site using sterile technique |  |  |  |
|  | 9) “time out”: verify patient, procedure, and insertion site are correct |  |  |  |
|  | 10) Inject anesthetic |  |  |  |
|  |  |  |  |  |
| Procedure | 11) Prepare the kit: open the tubes and assemble the manometer |  |  |  |
|  | 12) Insert needle about half its length, remove the stylet to check for CSF |  |  |  |
|  | 13) Slowly advance the needle about 1-2 mm at a time, removing the styelt to check for CSF after each advance |  |  |  |
|  | 14) CSF return noted |  |  |  |
|  | 15) Measure CSF opening pressure (optional) |  |  |  |
|  | 16) Collect specimens |  |  |  |
|  | 17) Measure CSF closing pressure (optional) |  |  |  |
|  | 18) Replace stylus |  |  |  |
|  | 19) Withdraw needle |  |  |  |
|  |  |  |  |  |
| Post-  Procedure | 20) Clean the area and apply dressing |  |  |  |
|  | 21) Throw away sharps |  |  |  |
|  | 22) Discard protective clothing |  |  |  |
|  | 23) Wash hands |  |  |  |
|  | 24) Properly label specimens |  |  |  |
|  | 25) Document procedure and update nursing and primary team |  |  |  |

Number of attempts at procedure: ______

Modified in May 2020 with permission from Joshua D. Lenchus, DO, RPh, FACP, SFHM; University of Miami – Jackson Memorial Hospital Center for Patient safety
