# Supplementary material for: Introducing a Comprehensive Framework for Competency-based Procedure Training
Source: J Gen Intern Med. 2025 Jul 8;40(15):3560–5. doi: 10.1007/s11606-025-09677-2 (PMC12612326; doi:10.1007/s11606-025-09677-2)
Supplement: Supplementary file 8 — Supplementary file8 (DOCX 31.5 KB) [file 11606_2025_9677_MOESM8_ESM.docx]

**Abdominal Paracentesis**
Performance Checklist

| Name |  | Date |  |
| --- | --- | --- | --- |
| Training Program |  | Procedure/Site |  |
| Training Year |  | Attending |  |

| Task  (chronological Order) | | Incompletely Performed | Completely Performed | Notes  (Complete if not done at all or incompletely performed) |
| --- | --- | --- | --- | --- |
| Pre-Procedure | 1)Review Patients’ chart, labs, and imaging (as relevant) |  |  |  |
|  | 2) Obtain informed consent: verify patient, procedure, and site |  |  |  |
|  | 3) Position patient: supine with HOB at 30 degrees |  |  |  |
|  | 4) Localize/mark needle insertion site: (anatomic/US): anterior axillary line |  |  |  |
|  | 5) Put on hat and mask; wash hands with soap and water |  |  |  |
|  | 6) Don protective clothing: sterile gown and sterile gloves |  |  |  |
|  | 7) Prepare site using chlorhexidine |  |  |  |
|  | 8) Drape site using sterile technique |  |  |  |
|  | 9) “time out”: verify patient, procedure, and insertion site are correct |  |  |  |
|  | 10) Inject anesthetic |  |  |  |
|  |  |  |  |  |
| Procedure | 11) Prepare the kit: assemble the needle/catheter device |  |  |  |
|  | 12) Make a nick and Insert needle: angular or Z-track technique |  |  |  |
|  | 13) Advance needle with negative pressure and once fluid is aspirated, advance an additional .5 cm to assure catheter has traversed peritoneum |  |  |  |
|  | 14) Holding needle still, guide catheter over needle |  |  |  |
|  | 15) Withdraw needle |  |  |  |
|  | 16) Aspirate fluid for diagnostic and/or therapeutic purpose |  |  |  |
|  | 17) Remove catheter |  |  |  |
|  |  |  |  |  |
| Post-  Procedure | 18) Clean the area, ensure no fluid leak, and apply dressing |  |  |  |
|  | 19) Throw away sharps |  |  |  |
|  | 20) Discard protective clothing |  |  |  |
|  | 21) Wash hands |  |  |  |
|  | 22) Properly label specimens |  |  |  |
|  | 23) Document procedure and update nursing and primary team |  |  |  |

Number of attempts at procedure: ______

Modified in May 2020 with permission from Joshua D. Lenchus, DO, RPh, FACP, SFHM; University of Miami – Jackson Memorial Hospital Center for Patient safety
