# Supplementary material for: Introducing a Comprehensive Framework for Competency-based Procedure Training
Source: J Gen Intern Med. 2025 Jul 8;40(15):3560–5. doi: 10.1007/s11606-025-09677-2 (PMC12612326; doi:10.1007/s11606-025-09677-2)
Supplement: Supplementary file 9 — Supplementary file9 (DOCX 14.9 KB) [file 11606_2025_9677_MOESM9_ESM.docx]

**STERILE TECHNIQUE**

Performance Checklist for Intern Orientation

**Name: ­­­­­ Date**:

**Proctor:**

| **Task** | | **Incompletely**  **Performed**  **(1 point)** | **Completely**  **Performed**  **(2 points)** | **Notes**  (Complete this section if learner does not complete tasks or incompletely performs) |
| --- | --- | --- | --- | --- |
| **Sterile Technique** | 1. List indications |  |  |  |
|  | 1. Gather equipment (sterile gown, mask, face shield/goggles, sterile gloves, surgical cap, shoe covers) |  |  |  |
|  | 1. Wash hands |  |  |  |
|  | 1. Don equipment while maintaining sterility |  |  |  |
|  | 1. Prepare self and area in sterile way; create a sterile field |  |  |  |
|  | 1. Use procedure equipment in a way that maintains self and area sterility |  |  |  |
|  | 1. List steps to properly dispose of contaminated equipment both during procedure and once procedure completed |  |  |  |
|  | 1. Remove equipment without contaminating self or others in environment |  |  |  |
|  | 1. Dispose of contaminated protective equipment appropriately |  |  |  |
|  | 1. Wash hands |  |  |  |
