# Supplementary material for: Introducing a Comprehensive Framework for Competency-based Procedure Training
Source: J Gen Intern Med. 2025 Jul 8;40(15):3560–5. doi: 10.1007/s11606-025-09677-2 (PMC12612326; doi:10.1007/s11606-025-09677-2)
Supplement: Supplementary file 10 — Supplementary file10 (DOCX 32.3 KB) [file 11606_2025_9677_MOESM10_ESM.docx]

**Thoracentesis**
Performance Checklist

| Name |  | Date |  |
| --- | --- | --- | --- |
| Training Program |  | Procedure/Site |  |
| Training Year |  | Attending |  |

| Task  (chronological Order) | | Incompletely Performed | Completely Performed | Notes  (Complete if not done at all or incompletely performed) |
| --- | --- | --- | --- | --- |
| Pre-Procedure | 1) Review Patient’s chart, labs, and imaging (as relevant) |  |  |  |
|  | 2) Use POCUS to determine the size and nature of the effusion  (Is an effusion present? Simple vs complex? Loculated?) |  |  |  |
|  | 3) Obtain informed consent: verify patient, procedure, and site |  |  |  |
|  | 4) Gather supplies: thoracentesis kit, etc |  |  |  |
|  | 5) Position patient:  a) posterior approach – standard upright position with arms lifted to help move scapula out of the way  b) lateral approach – recumbent position with arms lifted above the head (if patient unable to sit up for any specific reason) |  |  |  |
|  | 6) Use US to identify site  a) phased array probe – to assure adequate pocket to not injure lung and/or diaphragm  b) linear probe with color power doppler to identity any vessels in needle trajectory site |  |  |  |
|  | 7) Wash hands and don personal protective equipment (sterile gown and sterile gloves, eye protection) |  |  |  |
|  | 8) Prepare site using chlorhexidine |  |  |  |
|  | 9) Drape site using sterile technique |  |  |  |
|  | 10) “time out”: verify patient, procedure, and insertion site are correct | | | |
|  |  |  |  |  |
| Procedure | 11) Prepare and inject anesthetic in trajectory identified earlier |  |  |  |
|  | 12) Prepare the kit: assemble the needle/catheter device, and tubing |  |  |  |
|  | 13) Insert needle perpendicular to skin superior to inferior rib to avoid neuromuscular bundle |  |  |  |
|  | 14) Slowly advance the needle with dominant hand and control depth with non-dominant hand closer to skin while constantly applying negative pressure to skin |  |  |  |
|  | 15) Advance needle 5 mm after fluid is aspirated to assure catheter has entered pleural space |  |  |  |
|  | 16) Stabilize the needle and advance catheter over the needle |  |  |  |
|  | 17) Withdraw the needle |  |  |  |
|  | 18) Using 60 cc syringe, aspirate fluid for diagnostic studies |  |  |  |
|  | 19) Aspirate for therapeutic purpose – connect Y-shaped tubing to 60 cc syringe and manually withdraw fluid  a) stop removing fluid if 1) no more output, 2) if patient becomes symptomatic – severe coughing, increased SOB, chest pain, 3) if manometry > -20 cm H20 |  |  |  |
|  | 20) Stop suction prior to removing the catheter |  |  |  |
|  |  |  |  |  |
| Post-  Procedure | 21) Clean the area, ensure no bleeding, and apply dressing |  |  |  |
|  | 22) Throw away sharps |  |  |  |
|  | 23) Discard protective clothing |  |  |  |
|  | 24) Wash hands |  |  |  |
|  | 25) Properly label specimens |  |  |  |
|  | 26) Document procedure and update nursing and primary team |  |  |  |

Number of attempts at procedure: ______
