# Supplementary material for: Introducing a Comprehensive Framework for Competency-based Procedure Training
Source: J Gen Intern Med. 2025 Jul 8;40(15):3560–5. doi: 10.1007/s11606-025-09677-2 (PMC12612326; doi:10.1007/s11606-025-09677-2)
Supplement: Supplementary file 11 — Supplementary file11 (DOCX 15.1 KB) [file 11606_2025_9677_MOESM11_ESM.docx]

**Informed Consent**

Performance Checklist

**Name: ­­­­­ Date**: **____**

**Proctor:**

| **Task** | | **Incompletely**  **Performed**  **(1 point)** | **Completely**  **Performed**  **(2 points)** | **Notes**  (Complete this section if learner does not complete tasks or incompletely performs) |
| --- | --- | --- | --- | --- |
| **Informed Consent** | 1. List elements of informed consent |  |  |  |
|  | 1. Identify whether informed consent needs to be obtained |  |  |  |
|  | 1. Determine whether patient has capacity to make medical decision |  |  |  |
|  | 1. Identify appropriate surrogate decision maker |  |  |  |
|  | 1. Discuss indication for intervention |  |  |  |
|  | 1. Discuss risks of intervention |  |  |  |
|  | 1. Discuss benefits of intervention |  |  |  |
|  | 1. Discuss possible consequences of refusing intervention |  |  |  |
|  | 1. Briefly discuss potential alternative of intervention |  |  |  |
|  | 1. Correctly fill out consent form |  |  |  |
