# Supplementary material for: Introducing a Comprehensive Framework for Competency-based Procedure Training
Source: J Gen Intern Med. 2025 Jul 8;40(15):3560–5. doi: 10.1007/s11606-025-09677-2 (PMC12612326; doi:10.1007/s11606-025-09677-2)

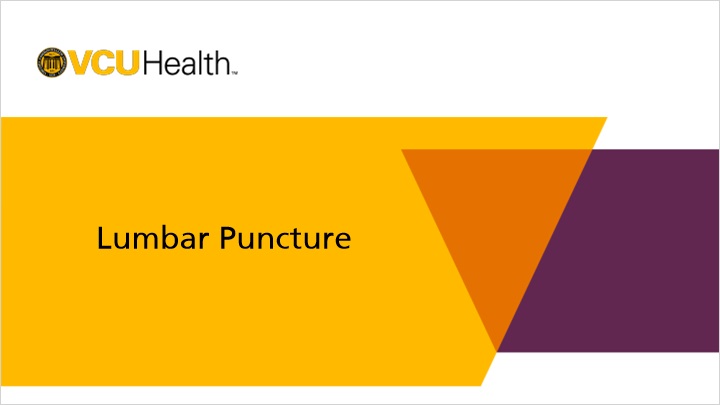


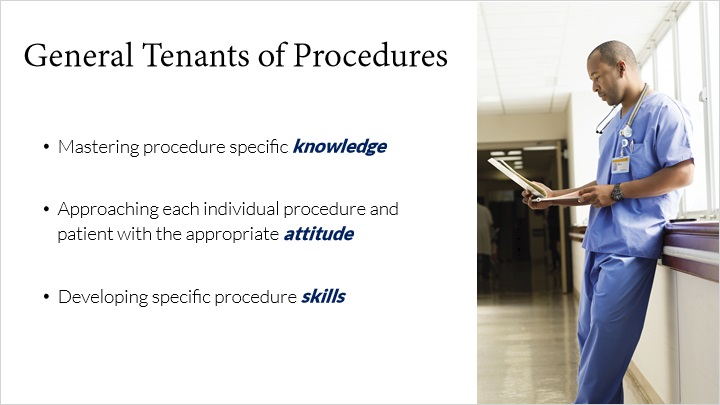


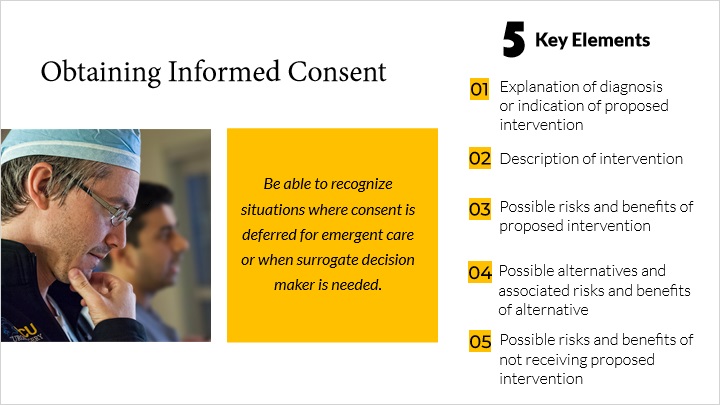


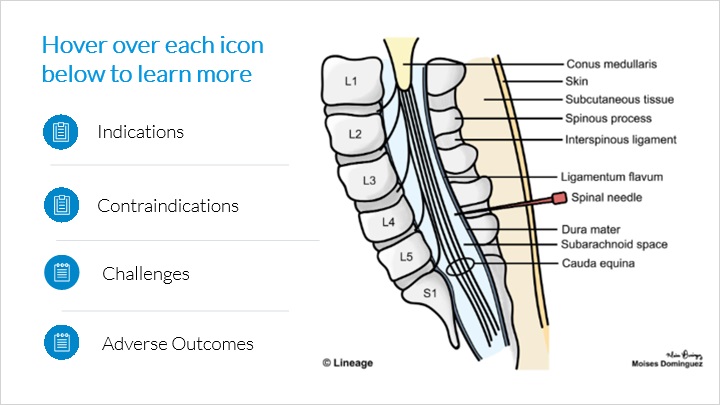


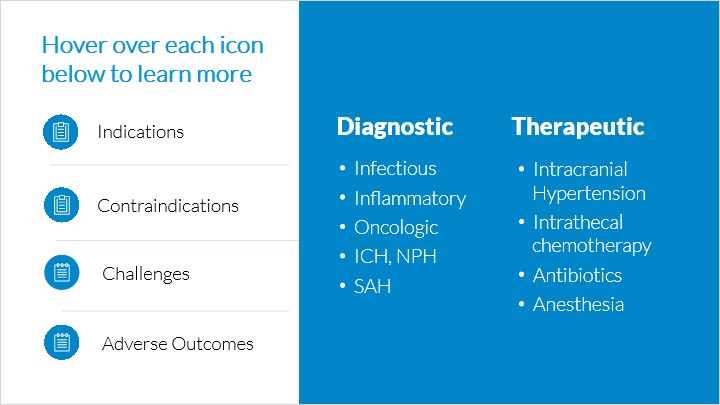


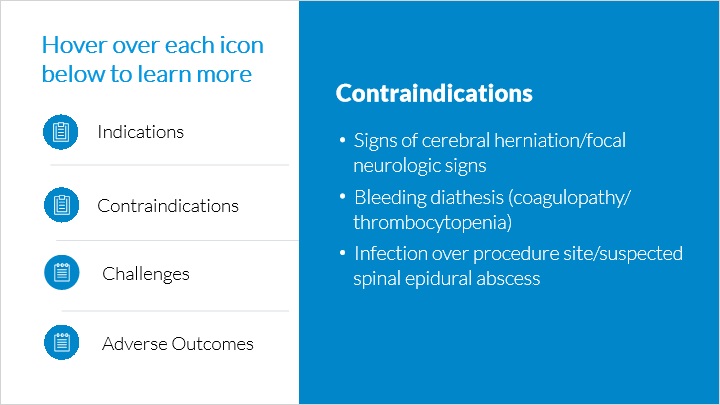


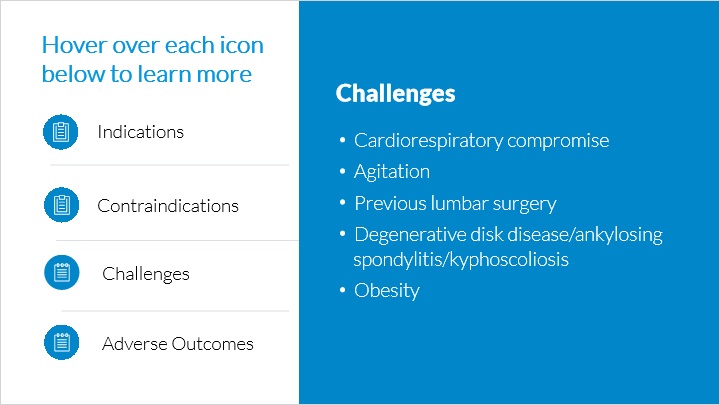


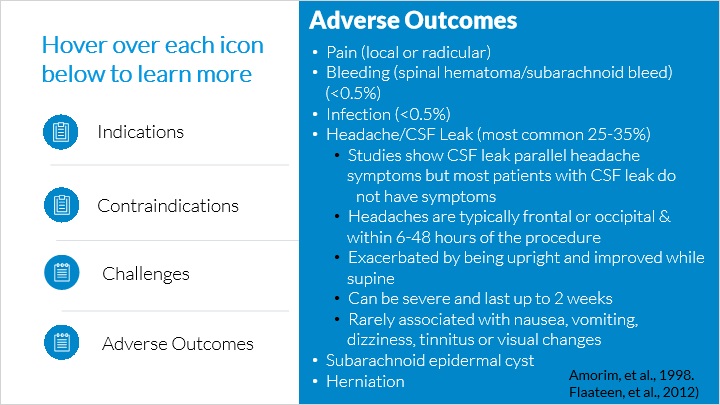


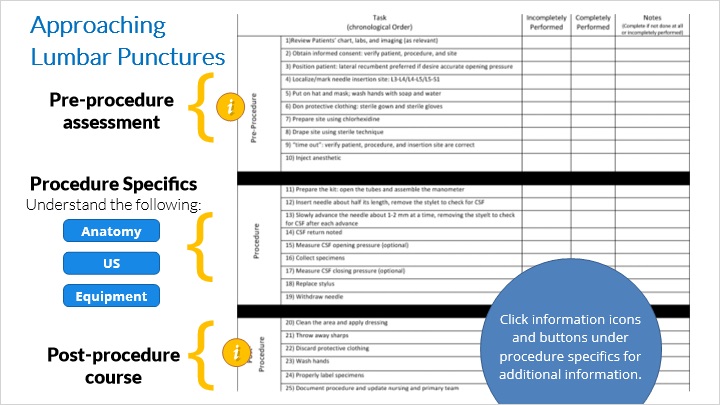


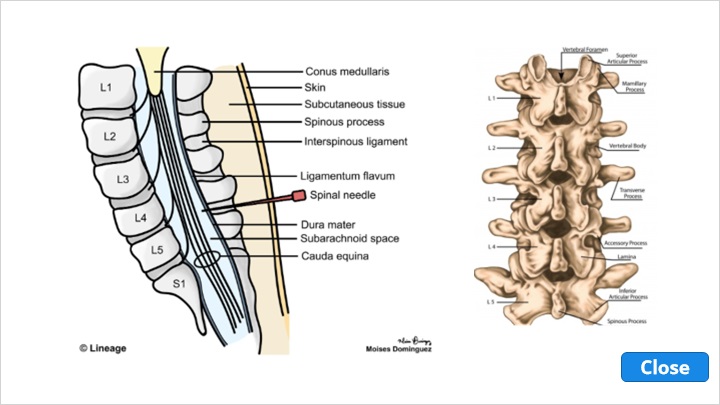


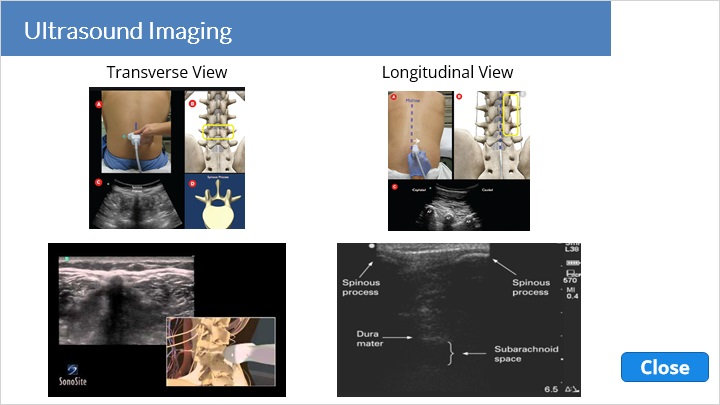


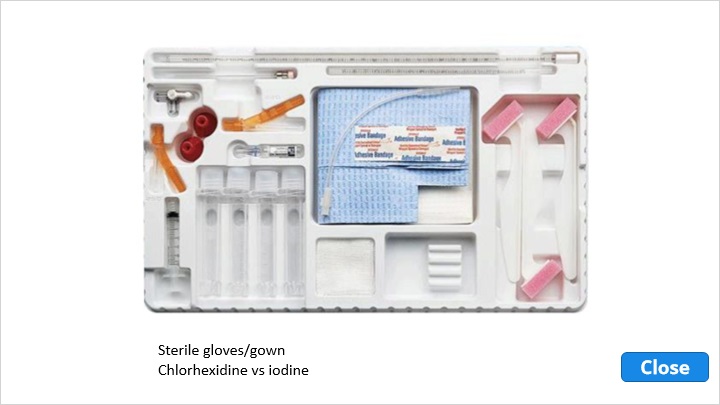


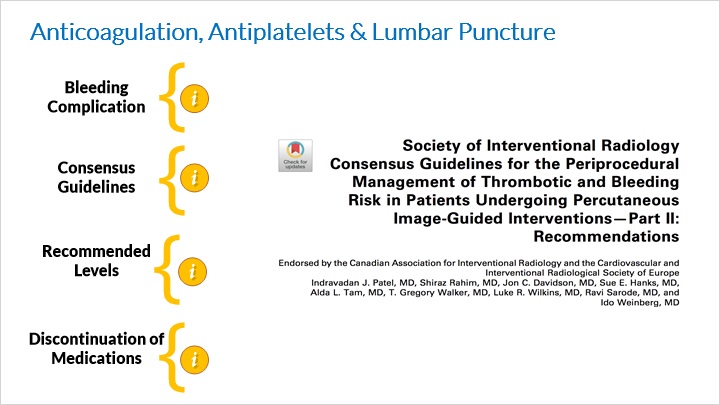


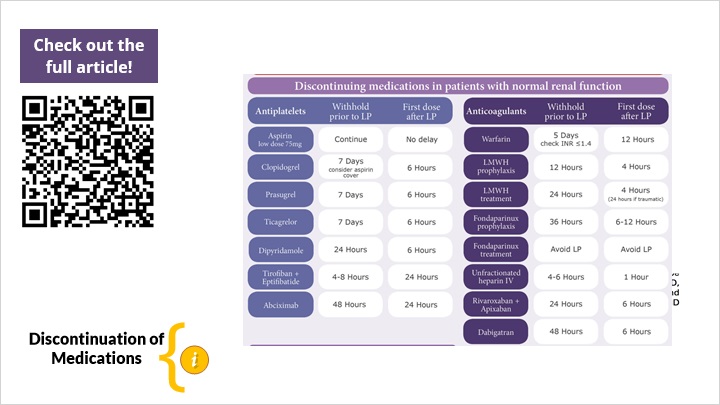


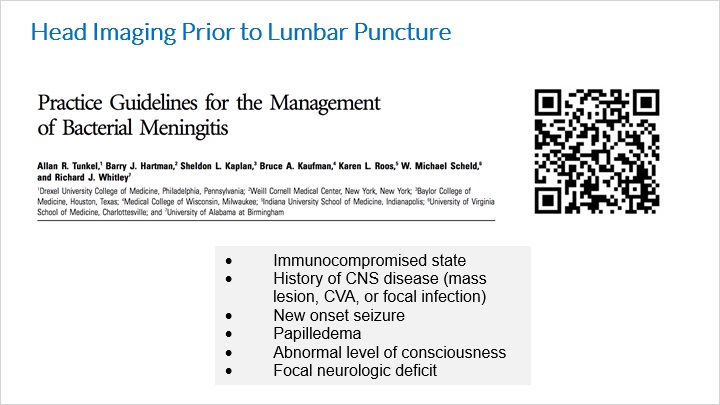


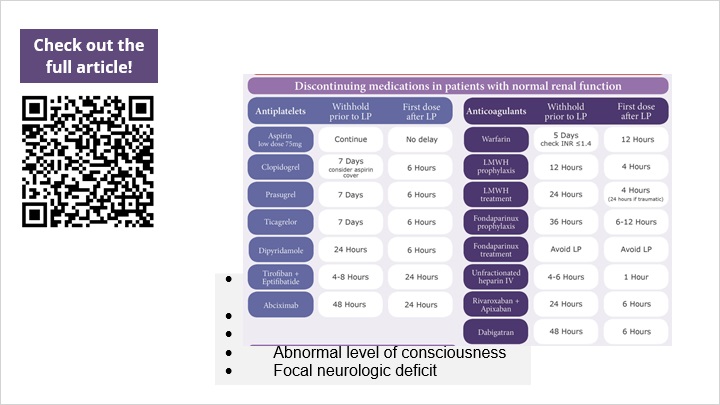


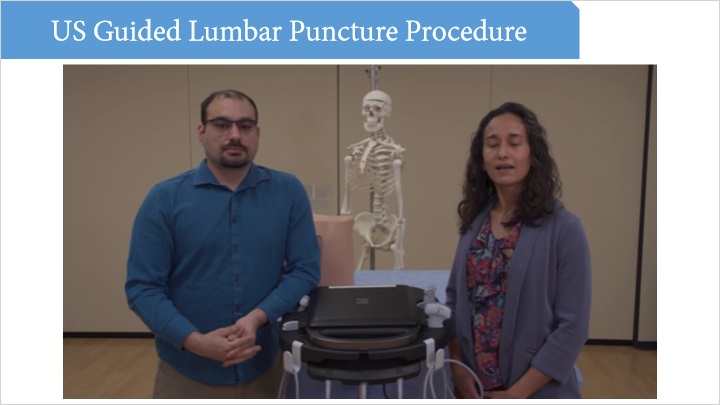


**Notes: video of procedure**


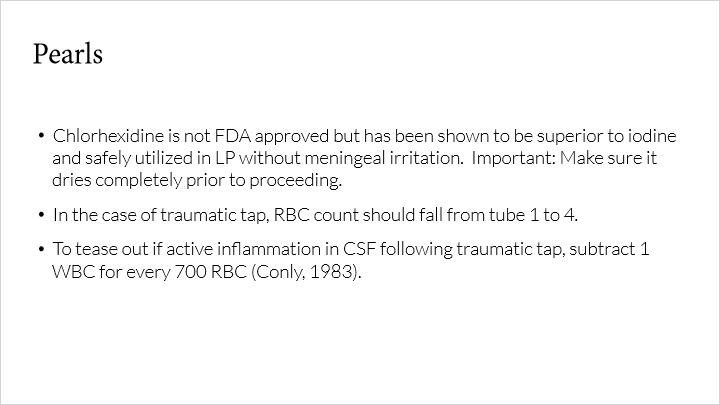


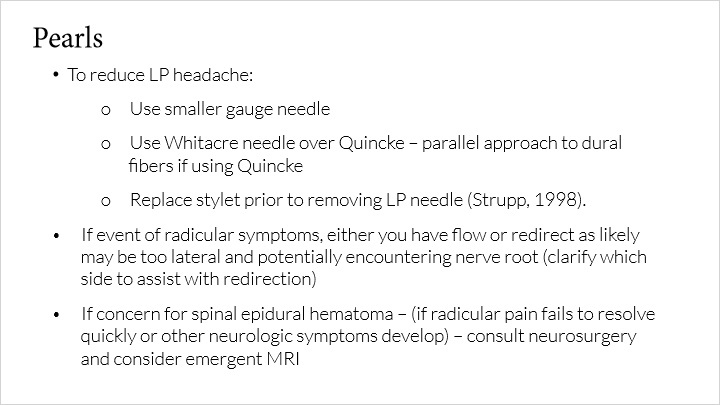


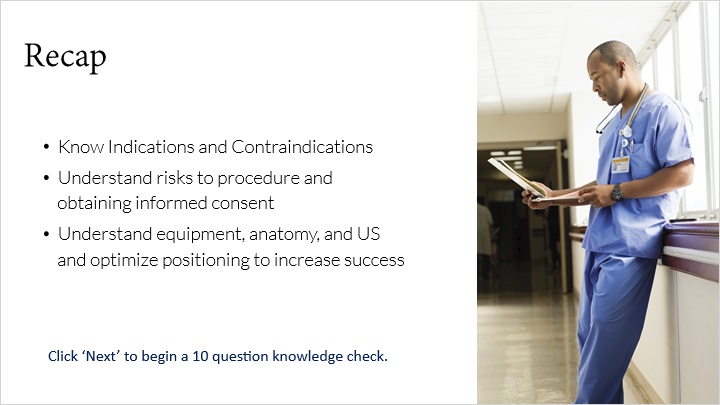


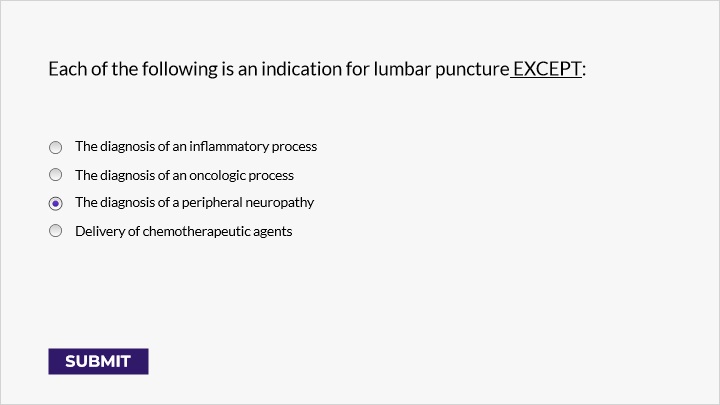


| Correct | Choice |
| --- | --- |
|  | The diagnosis of an inflammatory process |
|  | The diagnosis of an oncologic process |
| X | The diagnosis of a peripheral neuropathy |
|  | Delivery of chemotherapeutic agents |

**Feedback when correct:**

You selected the correct response.

**Feedback when incorrect:**

You did not answer correctly.


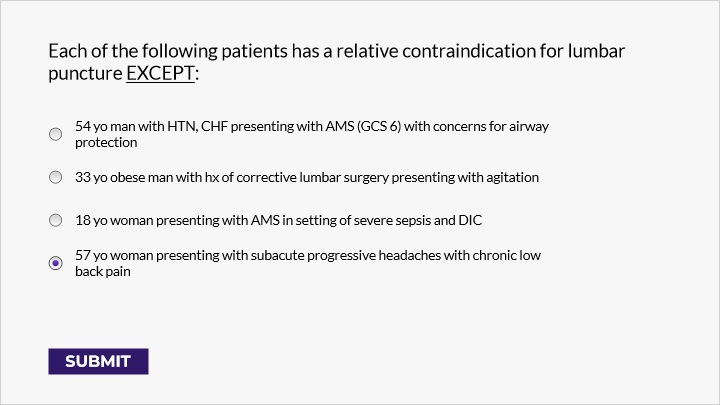


| Correct | Choice |
| --- | --- |
|  | 54 yo man with HTN, CHF presenting with AMS (GCS 6) with concerns for airway protection |
|  | 33 yo obese man with hx of corrective lumbar surgery presenting with agitation |
|  | 18 yo woman presenting with AMS in setting of severe sepsis and DIC |
| X | 57 yo woman presenting with subacute progressive headaches with chronic low back pain |

**Feedback when correct:**

You selected the correct response.

**Feedback when incorrect:**

You did not answer correctly.


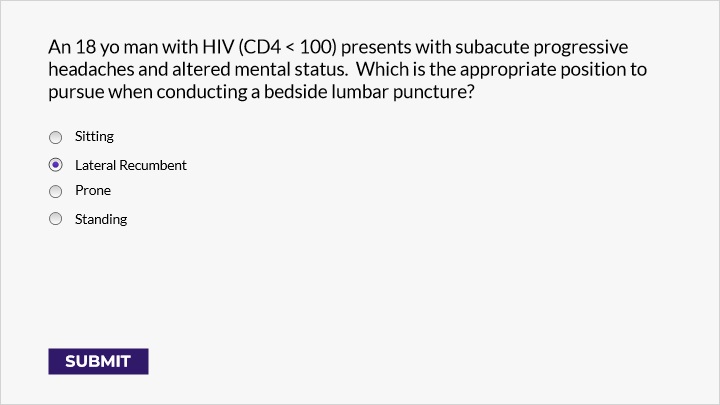


| Correct | Choice |
| --- | --- |
|  | Sitting |
| X | Lateral Recumbent |
|  | Prone |
|  | Standing |

**Feedback when correct:**

The lateral recumbent position is necessary to obtain an opening pressure which is vital in this patient who is at risk for cryptococcal meningitis.

**Feedback when incorrect:**

You did not answer correctly.


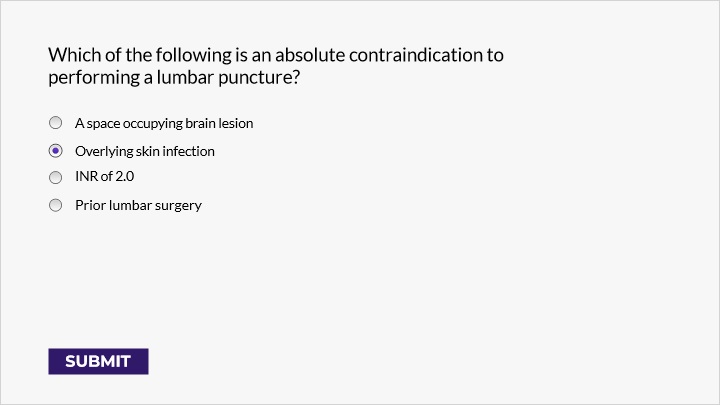


| Correct | Choice |
| --- | --- |
|  | A space occupying brain lesion |
| X | Overlying skin infection |
|  | INR of 2.0 |
|  | Prior lumbar surgery |

**Feedback when correct:**

You cannot traverse a superficial infection or epidural abscess as this may seed deeper structures/compartments.

While cerebral herniation is the most feared complication of an LP, not all space occupying lesions lead to increased ICP and not all conditions with increased ICP will result in herniation following an LP.

Patients with any of the following clinical findings should have a head CT prior to LP and a discussion with neurosurgery should be considered prior to LP if there are any concerning findings found on CT: AMS, focal neurologic signs, papilledema, recent seizure, impaired cellular immunity.

INR of 2 is a relative contraindication. While LP bleeding risk is low, the consequences of a bleed are significant. Increased bleeding risk factors should be taken in context with all patient factors and weighed against the urgency and need of the procedure.

Hardware is a relative contraindication as it may increase risk and challenges with approach and related infections.

**Feedback when incorrect:**

You did not answer correctly.


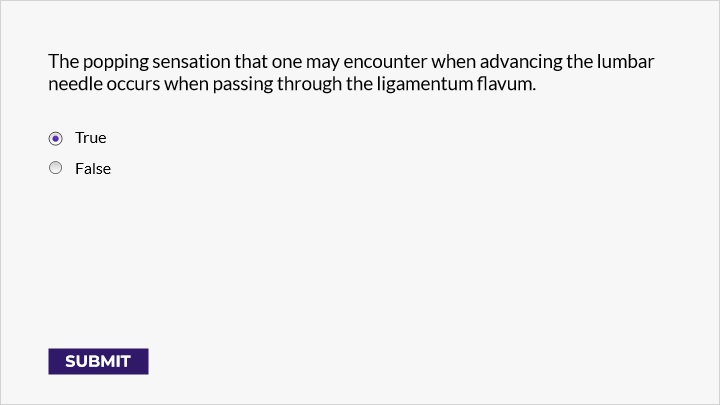


| Correct | Choice |
| --- | --- |
| X | True |
|  | False |

**Feedback when correct:**

This sensation is not always appreciated but typically when it is encountered it is when the needle is passing through the ligamentum flavum.

**Feedback when incorrect:**

You did not answer correctly.


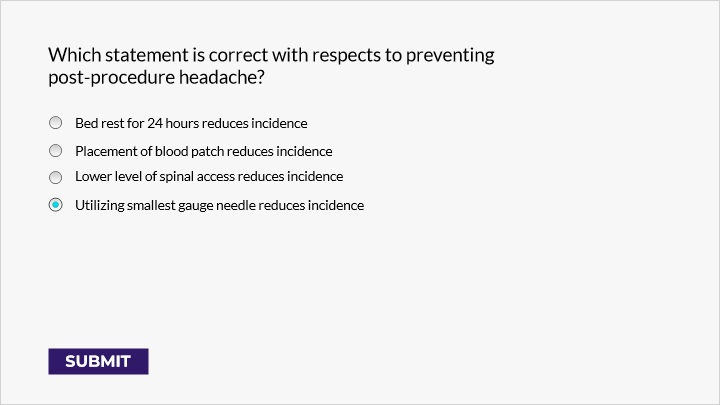


| Correct | Choice |
| --- | --- |
|  | Bed rest for 24 hours reduces incidence |
|  | Placement of blood patch reduces incidence |
|  | Lower level of spinal access reduces incidence |
| X | Utilizing smallest gauge needle reduces incidence |

**Feedback when correct:**

Evidence shows atraumatic needles have lower rates of post-procedure headache. Larger meta-analysis did not show benefit of laying supine reducing post-procedure headache. Blood patches can be utilized to manage severe persistent post-procedure headaches with 95 % efficacy.

**Feedback when incorrect:**

You did not answer correctly.


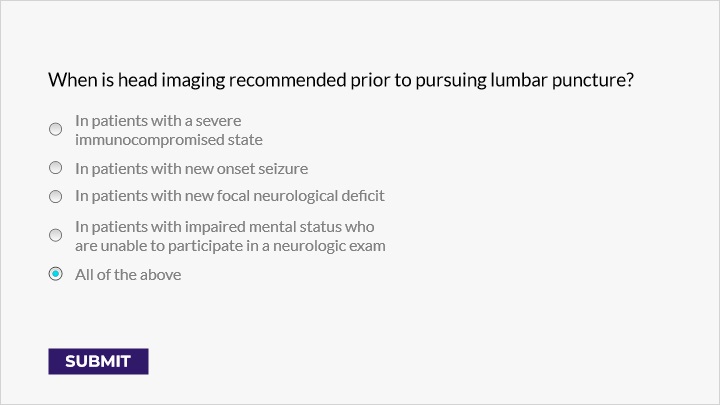


| Correct | Choice |
| --- | --- |
|  | In patients with a severe immunocompromised state |
|  | In patients with new onset seizure |
|  | In patients with new focal neurological deficit |
|  | In patients with impaired mental status who are unable to participate in a neurologic exam |
| X | All of the above |

**Feedback when correct:**

Imaging is recommended for patients who cannot have thorough neurologic examination at risk for or with evidence of potential space occupying intracranial lesions which would place them at higher risk for herniation.

**Feedback when incorrect:**

You did not answer correctly.


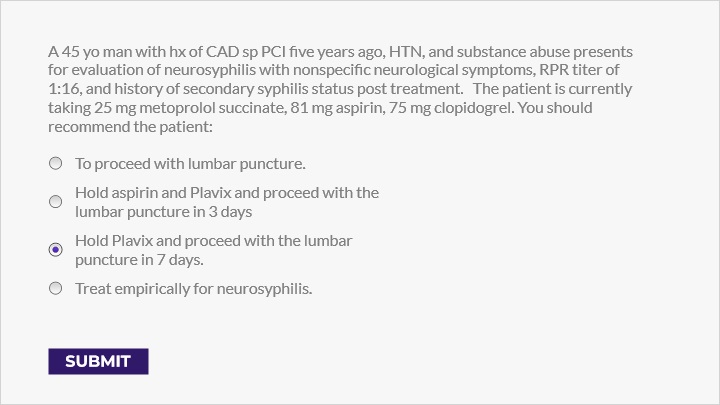


| Correct | Choice |
| --- | --- |
|  | To proceed with lumbar puncture.​ |
|  | Hold aspirin and Plavix and proceed with the lumbar puncture in 3 days​ |
| X | Hold Plavix and proceed with the lumbar puncture in 7 days.​ |
|  | Treat empirically for neurosyphilis. |

**Feedback when correct:**

It is safe to perform LP in patients on aspirin but it is recommended to hold clopidogrel for 7 days. Typically PLT goals > 40 are desired but studies have shown > 20 is adequate but take into consideration other factors that may lead to bleeding or difficulty obtaining access. INR goals are typically < 1.5.​ It is recommended to hold one dose of pharmacologic DVT ppx. DOACs should be held a minimum 48 hours if they have normal renal function. Patients on warfarin require correction of INR for more urgent procedures. ​

It is recommended to hold one dose of pharmacologic DVT ppx. DOACs should be held a minimum 48 hours if they have normal renal function. Patients on warfarin require correction of INR for more urgent procedures. ​

**Feedback when incorrect:**

You did not answer correctly.


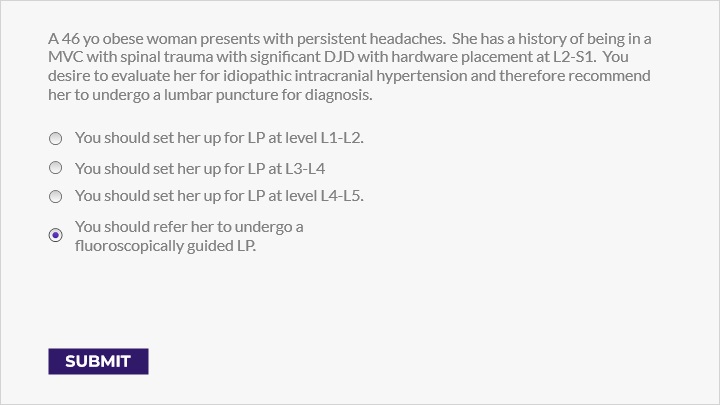


| Correct | Choice |
| --- | --- |
|  | You should set her up for LP at level L1-L2. |
|  | You should set her up for LP at L3-L4​ |
|  | You should set her up for LP at level L4-L5. |
| X | You should refer her to undergo a fluoroscopically guided LP. |

**Feedback when correct:**

You selected the correct response.

**Feedback when incorrect:**

You did not answer correctly.


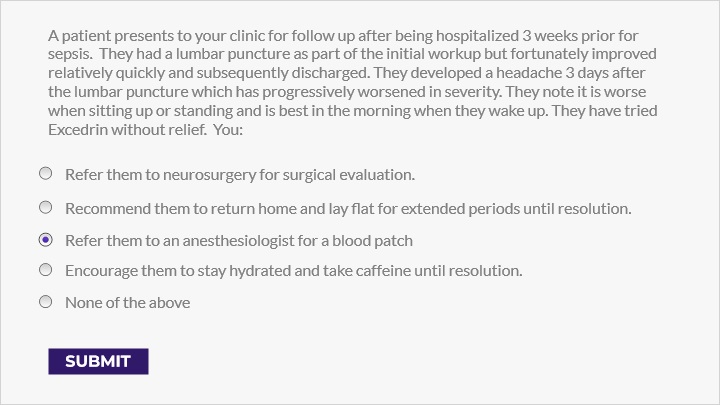


| Correct | Choice |
| --- | --- |
|  | Refer them to neurosurgery for surgical evaluation.​ |
|  | Recommend them to return home and lay flat for extended periods until resolution.​ |
| X | Refer them to an anesthesiologist for a blood patch |
|  | Encourage them to stay hydrated and take caffeine until resolution.​ |
|  | None of the above |

**Feedback when correct:**

You selected the correct response.

**Feedback when incorrect:**

You did not answer correctly.

### CORRECT (Slide Layer)


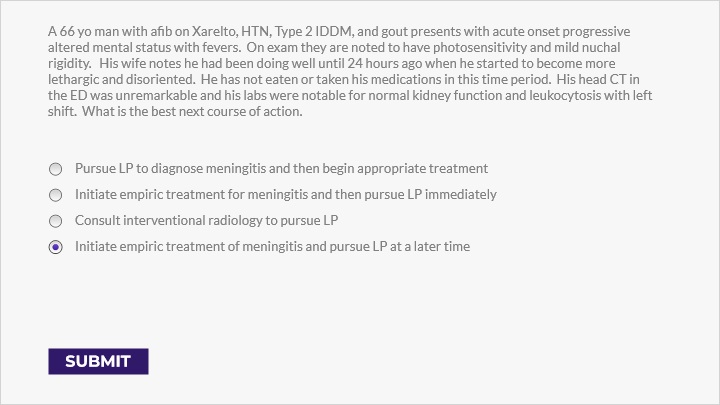


| Correct | Choice |
| --- | --- |
|  | Pursue LP to diagnose meningitis and then begin appropriate treatment​ |
|  | Initiate empiric treatment for meningitis and then pursue LP immediately​ |
|  | Consult interventional radiology to pursue LP​ |
| X | Initiate empiric treatment of meningitis and pursue LP at a later time |

**Feedback when correct:**

You selected the correct response.

**Feedback when incorrect:**

You did not answer correctly.

### INCORRECT (Slide Layer)

*(Multiple Choice, 10 points, 1 attempt permitted)*


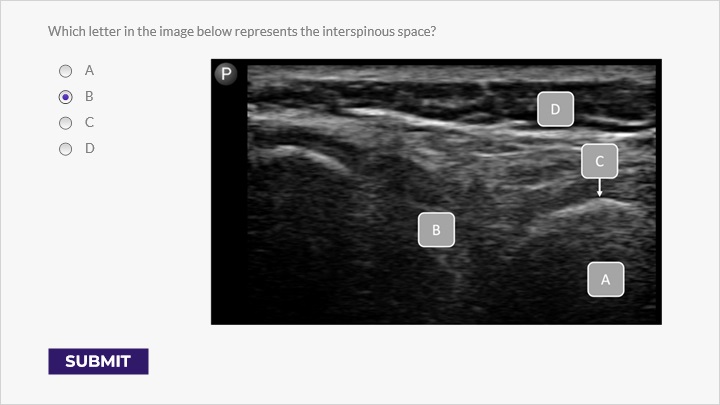


| Correct | Choice |
| --- | --- |
|  | A |
| X | B​ |
|  | C |
|  | D |

**Feedback when correct:**

You selected the correct response.

**Feedback when incorrect:**

You did not answer correctly.


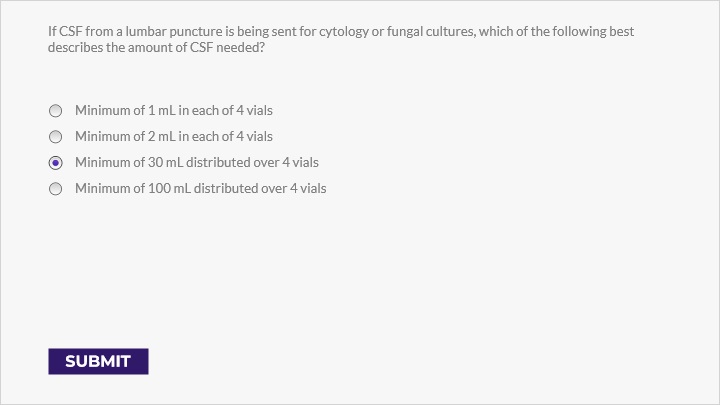


| Correct | Choice |
| --- | --- |
|  | Minimum of 1 mL in each of 4 vials |
|  | Minimum of 2 mL in each of 4 vials​ |
| X | Minimum of 30 mL distributed over 4 vials​ |
|  | Minimum of 100 mL distributed over 4 vials |

**Feedback when correct:**

You selected the correct response.

**Feedback when incorrect:**

You did not answer correctly.


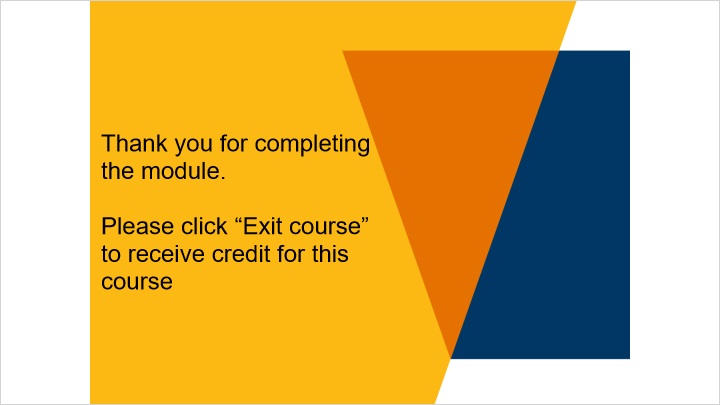

Supplement: Supplementary file 12 — Supplementary file12 (DOC 2.08 MB) [file 11606_2025_9677_MOESM12_ESM.doc]
