# Supplementary material for: Introducing a Comprehensive Framework for Competency-based Procedure Training
Source: J Gen Intern Med. 2025 Jul 8;40(15):3560–5. doi: 10.1007/s11606-025-09677-2 (PMC12612326; doi:10.1007/s11606-025-09677-2)

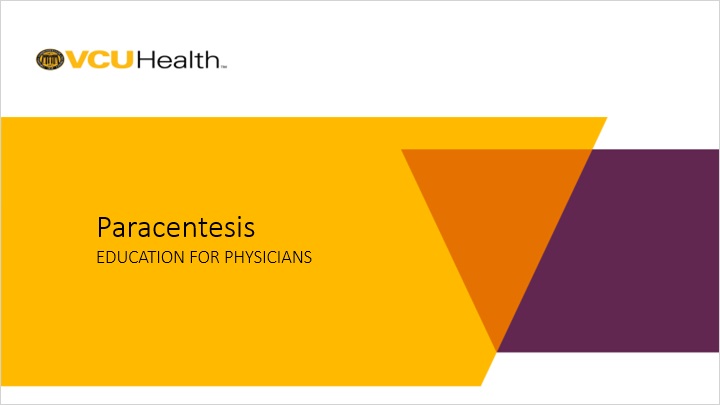


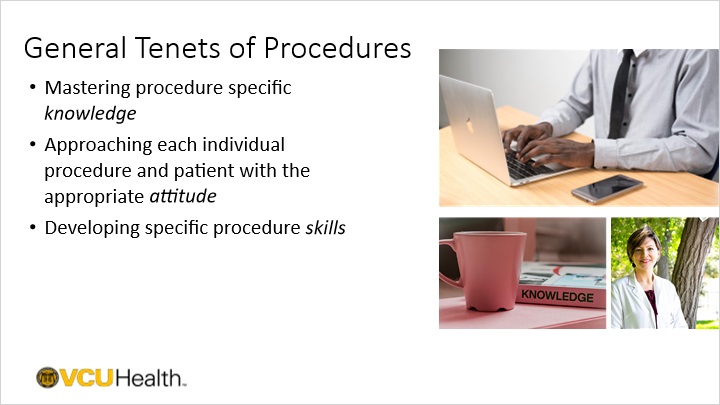


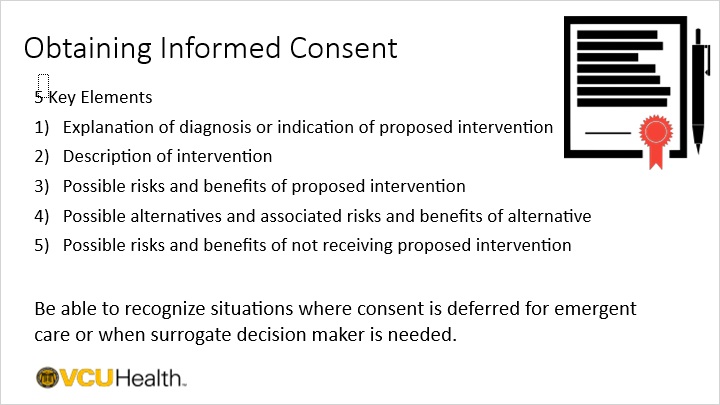


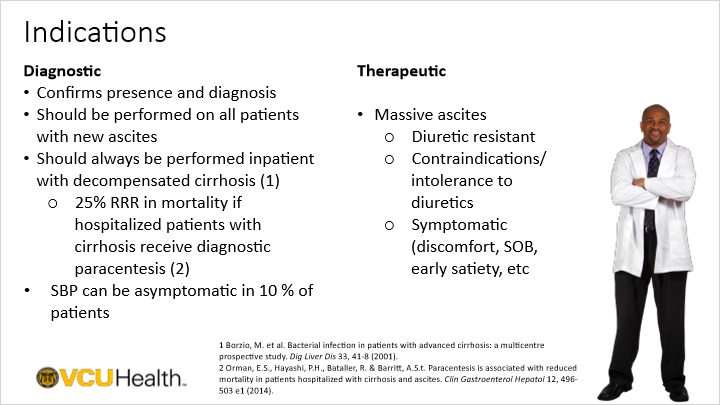


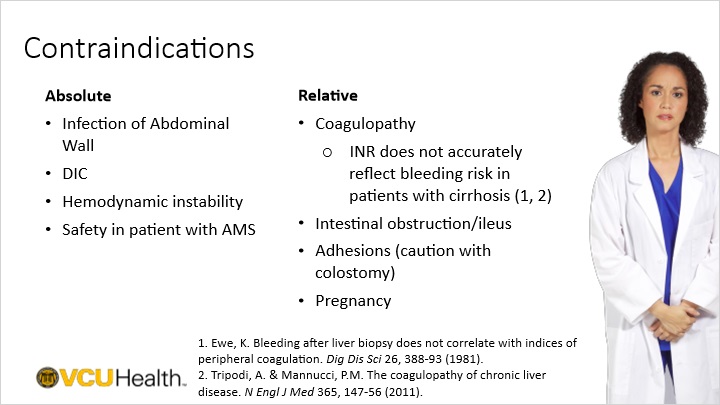


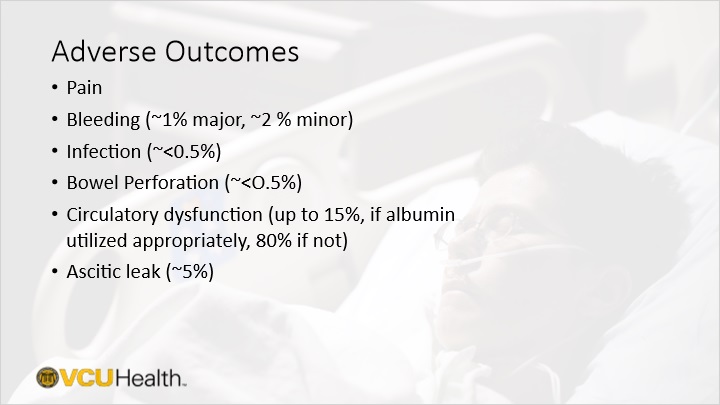


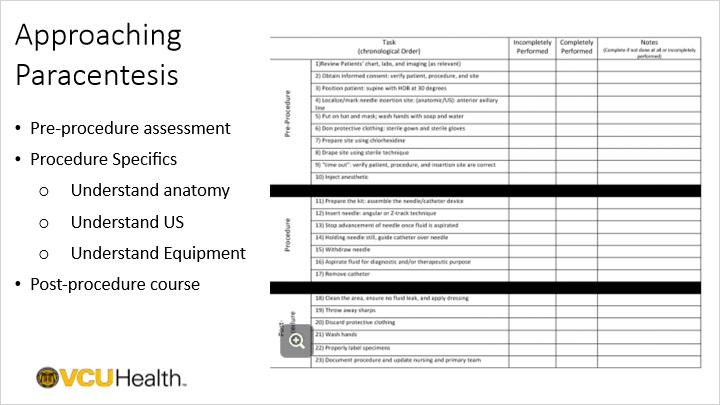


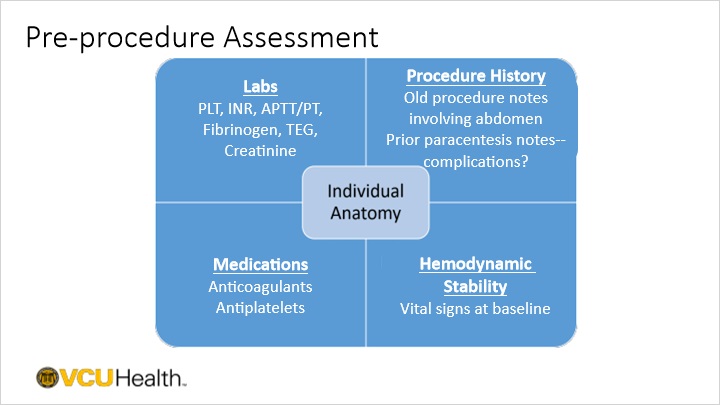


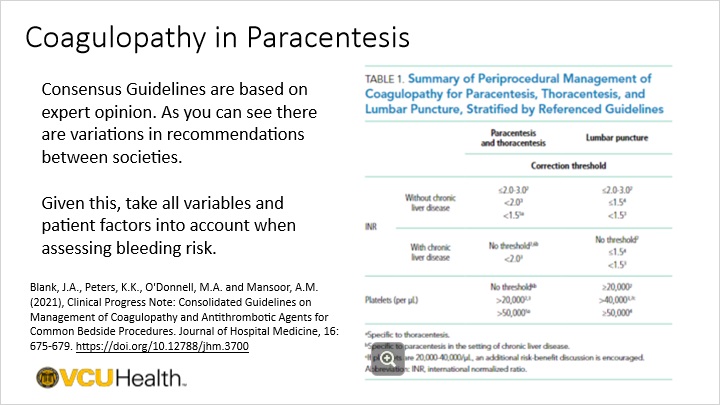


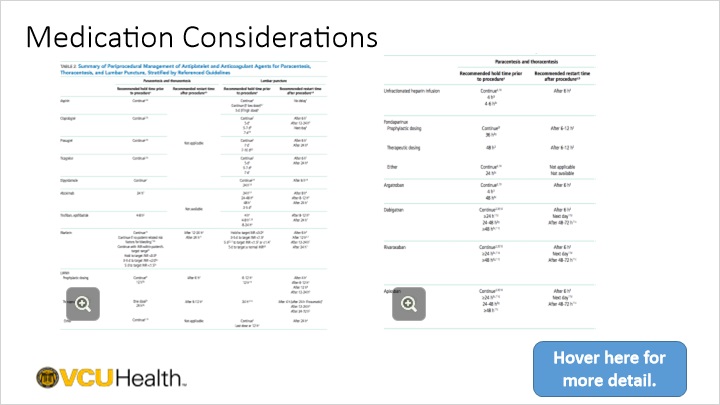


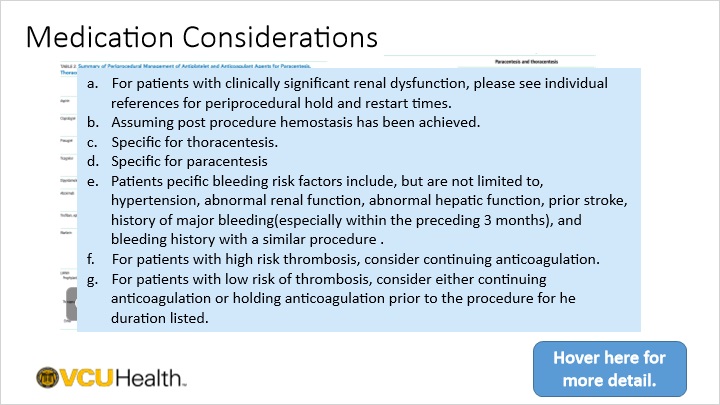


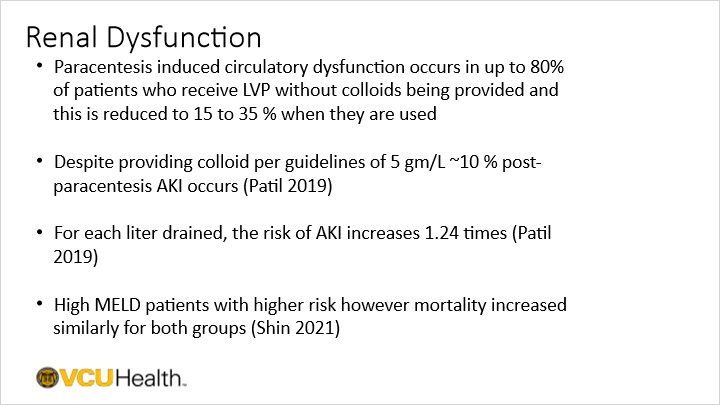


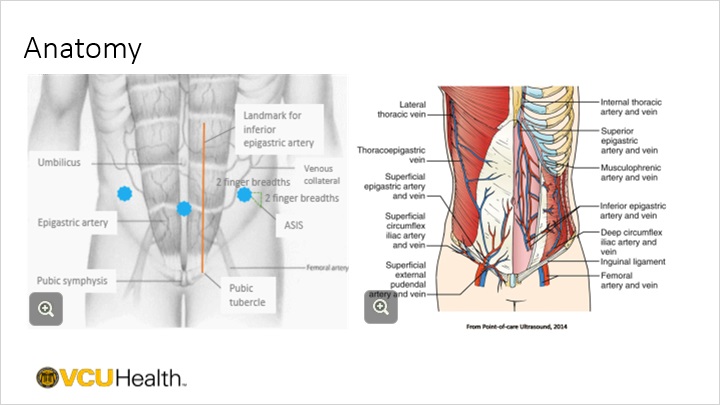


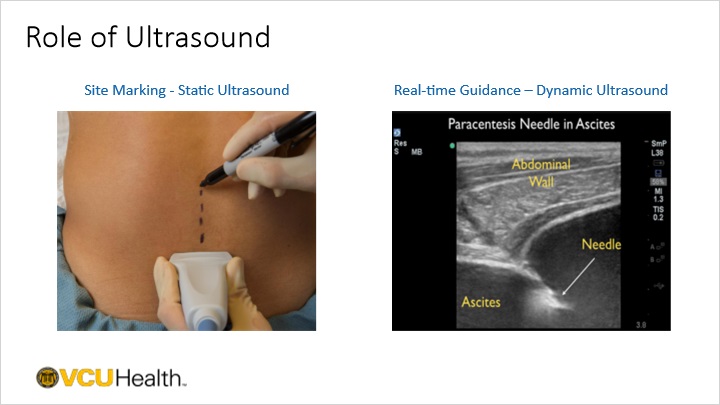


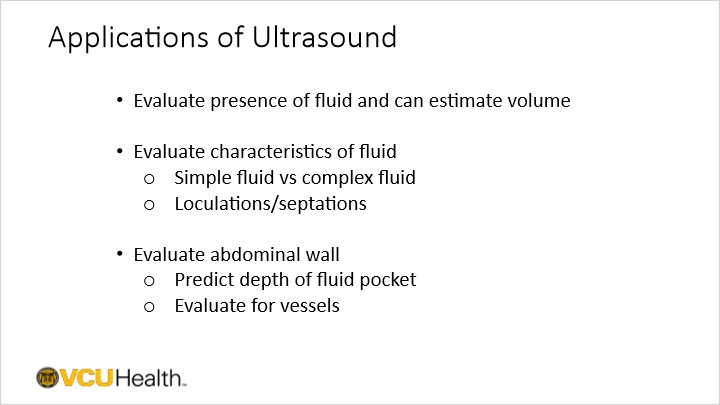


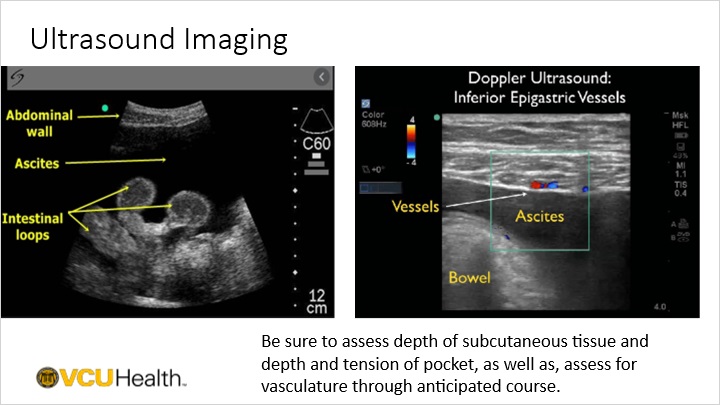


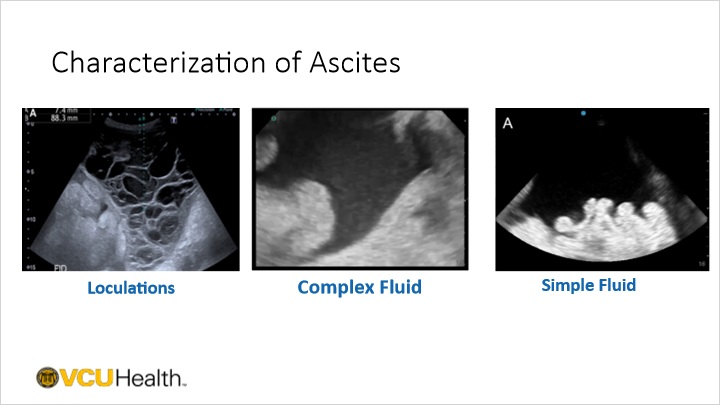


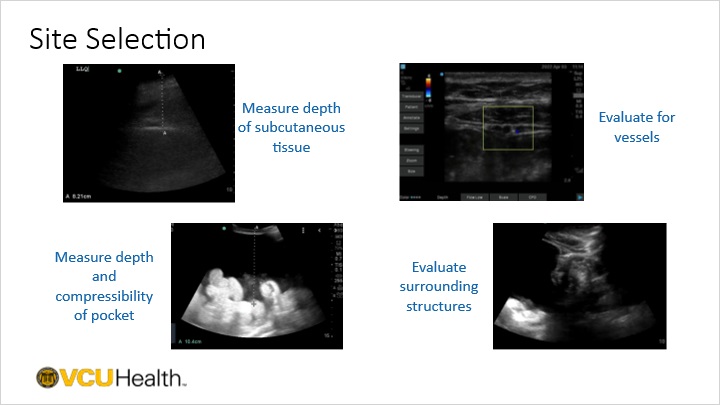


**Notes:**


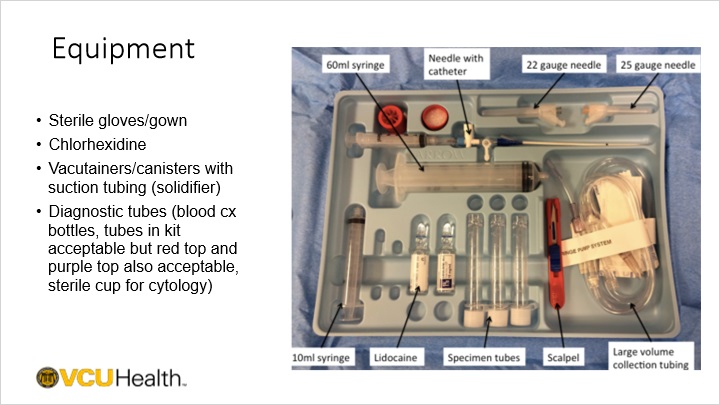


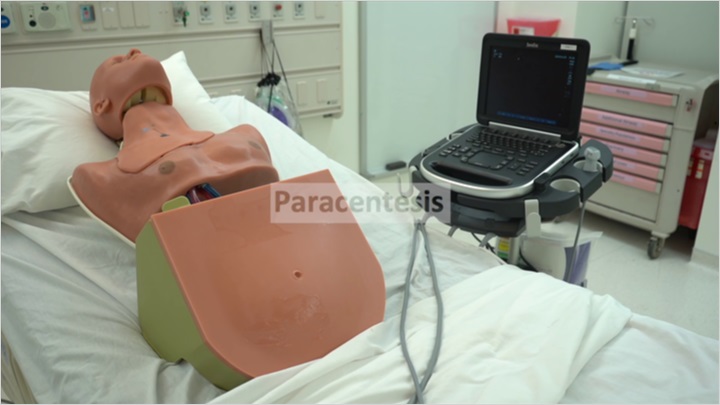


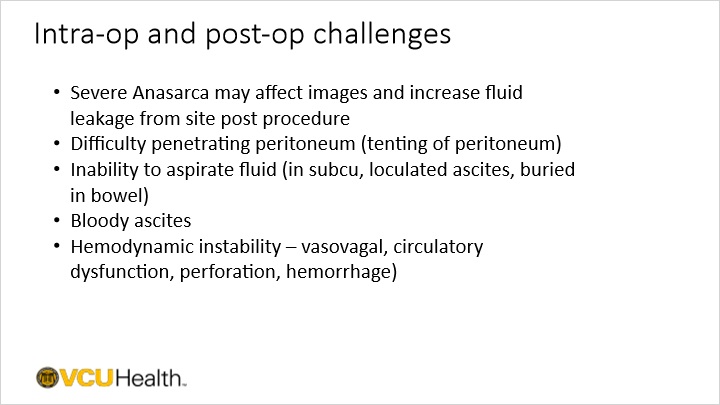


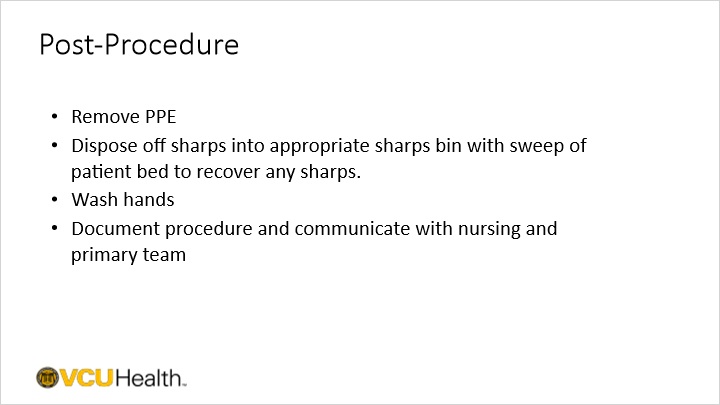


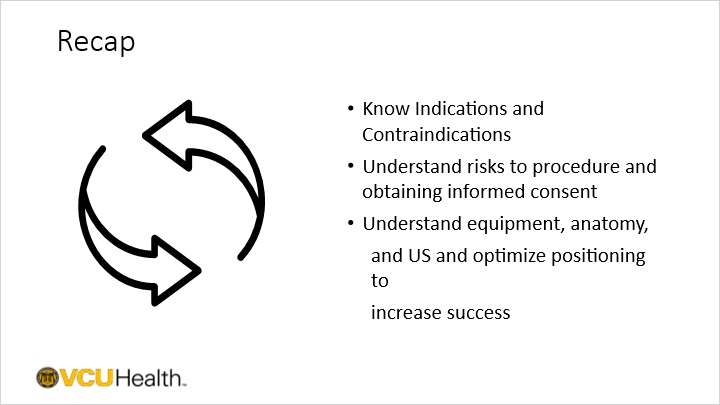


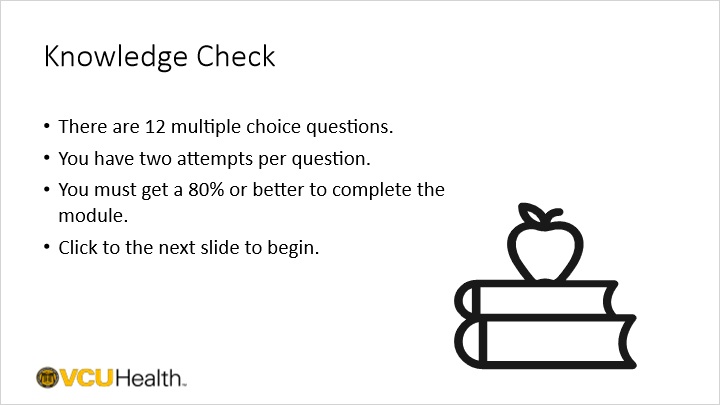


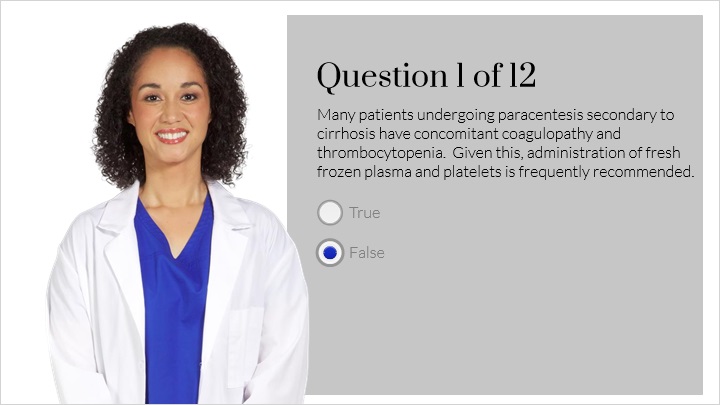


| Correct | Choice |
| --- | --- |
|  | True |
| X | False |

**Feedback when correct:**

In most cases, it is not recommended to provide patients with product prior to procedure. This being said, evaluate each patient individually. Recognize clinical changes and deviation from the patient’s baseline. There is evidence to suggest TEG’s may be helpful to further distinguish bleeding risk in more complicated patients. Remember, although major risk of bleeding is ~ 1 %, risk of mortality approaches 40% for those that incur significant bleeding.

**Feedback when incorrect:**

You did not select the correct response.


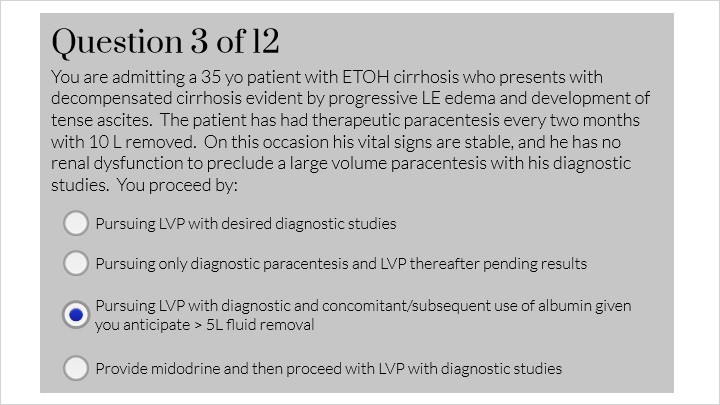


| Correct | Choice |
| --- | --- |
|  | Pursuing LVP with desired diagnostic studies |
|  | Pursuing only diagnostic paracentesis and LVP thereafter pending results |
| X | Pursuing LVP with diagnostic and concomitant/subsequent use of albumin given you anticipate > 5L fluid removal |
|  | Provide midodrine and then proceed with LVP with diagnostic studies |

**Feedback when correct:**

Albumin is recommended when taking off > 5 L. Doing so during or immediately after is recommend to prevent circulatory dysfunction. Assure patient receives previously/simultaneously if documented issues with prior procedures and watch VS closely during procedure to terminate procedure if any fluctuation in vital signs. If prior history of circulatory dysfunction post procedure, then one might utilize midodrine in addition to albumin and/or proceed with more conservative volume removal.

**Feedback when incorrect:**

You did not select the correct response.


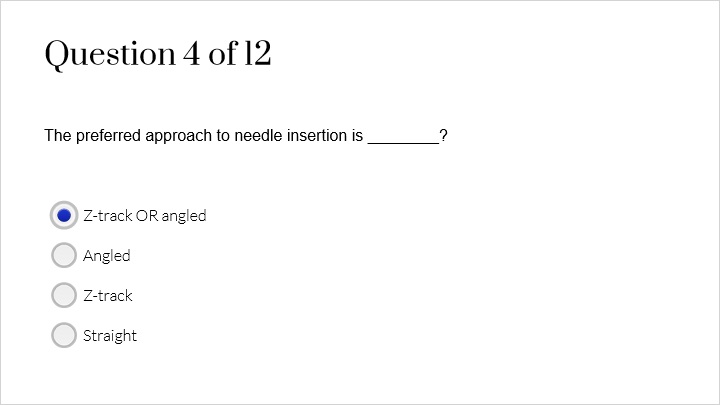


| Correct | Choice |
| --- | --- |
| X | Z-track OR angled |
|  | Angled |
|  | Z-track |
|  | Straight |

**Feedback when correct:**

Pursing an angled or z-track approach may help reduce complication of ascitic leak. In some instances, habitus and pocket size may preclude ability to safely perform these maneuvers however. Talking with professionals across the country, it is relatively rare for people to actually use the z-track technique.

**Feedback when incorrect:**

You did not select the correct response.


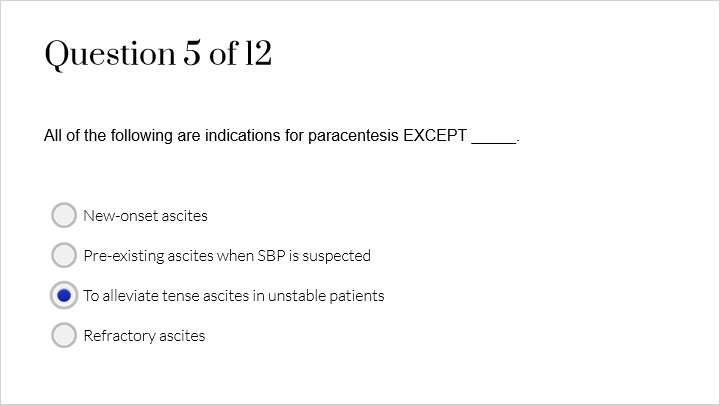


| Correct | Choice |
| --- | --- |
|  | New-onset ascites |
|  | Pre-existing ascites when SBP is suspected |
| X | To alleviate tense ascites in unstable patients |
|  | Refractory ascites |

**Feedback when correct:**

Assuring patient is stable prior to procedure is vital. Although diagnostic studies may inherently have lower risk, one can treat empirically until assured patient stable.

**Feedback when incorrect:**

You did not select the correct response.


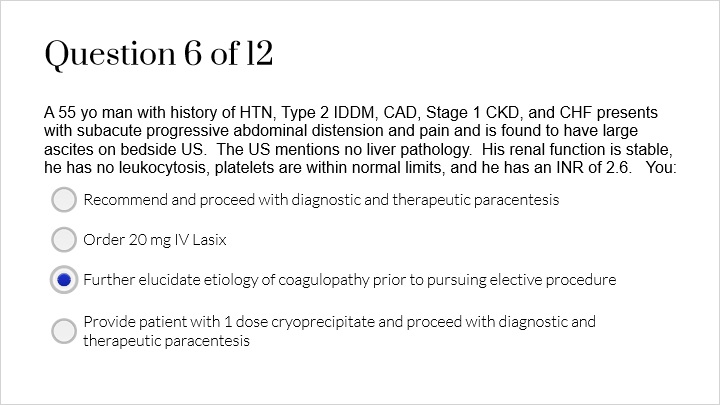


| Correct | Choice |
| --- | --- |
|  | Recommend and proceed with diagnostic and therapeutic paracentesis |
|  | Order 20 mg IV Lasix |
| X | Further elucidate etiology of coagulopathy prior to pursuing elective procedure |
|  | Provide patient with 1 dose cryoprecipitate and proceed with diagnostic and therapeutic paracentesis |

**Feedback when correct:**

In most instances, paracentesis is performed on patients with cirrhosis who typically have coagulopathies and thrombocytopenia. For those patients with no evidence of cirrhosis, it is important to assess bleeding risk and modify factors as needed.

**Feedback when incorrect:**

You did not select the correct response.


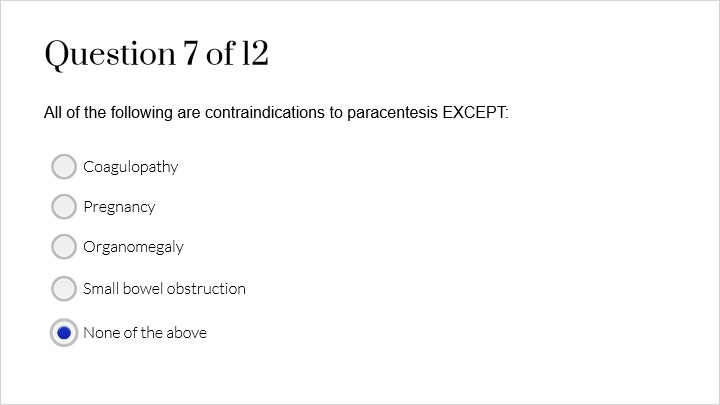


| Correct | Choice |
| --- | --- |
|  | Coagulopathy |
|  | Pregnancy |
|  | Organomegaly |
|  | Small bowel obstruction |
| X | None of the above |

**Feedback when correct:**

The above are all relative contraindications to paracentesis. Assessing patient with US to assure adequate pocket in patients with SBO, organomegaly, or pregnancy will determine if procedure able to be performed safely. Coagulopathy is not uncommon in patients with cirrhosis, and one should be aware of deviations from prior analysis in conjunction with other factors that may affecting bleeding risk.

**Feedback when incorrect:**

You did not select the correct response.


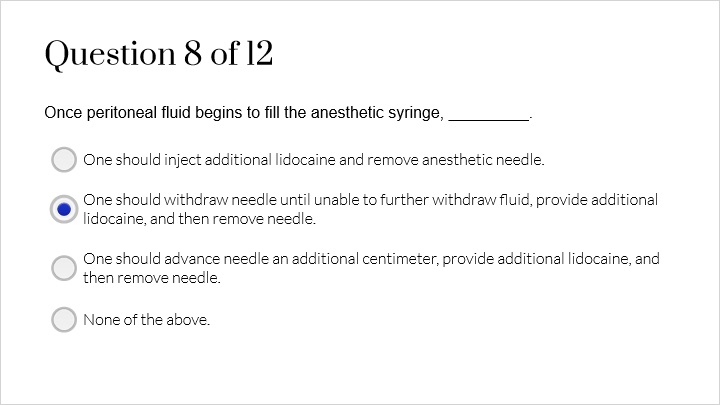


| Correct | Choice |
| --- | --- |
|  | One should inject additional lidocaine and remove anesthetic needle. |
| X | One should withdraw needle until unable to further withdraw fluid, provide additional lidocaine, and then remove needle. |
|  | One should advance needle an additional centimeter, provide additional lidocaine, and then remove needle. |
|  | None of the above. |

**Feedback when correct:**

Patients tend to be most sensitive at the skin insertion site and the peritoneum. Once fluid is aspirated withdraw needle slightly until unable to further withdrawal fluid and provide additional lidocaine, one can subsequently inject lidocaine with removal of the needle.

**Feedback when incorrect:**

You did not select the correct response.


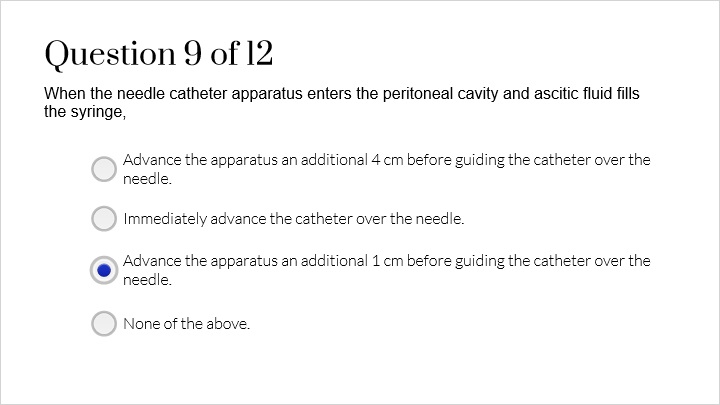


| Correct | Choice |
| --- | --- |
|  | Advance the apparatus an additional 4 cm before guiding the catheter over the needle. |
|  | Immediately advance the catheter over the needle. |
| X | Advance the apparatus an additional 1 cm before guiding the catheter over the needle. |
|  | None of the above. |

**Feedback when correct:**

Be mindful of depth and compressibility of pocket when advancing needle catheter apparatus. Once fluid is aspirated, advancing an additional centimeter is ideal to assure the catheter has transposed the peritoneum adequately prior to advancing catheter fully over the needle.

**Feedback when incorrect:**

You did not select the correct response.

### Incorrect (Slide Layer)


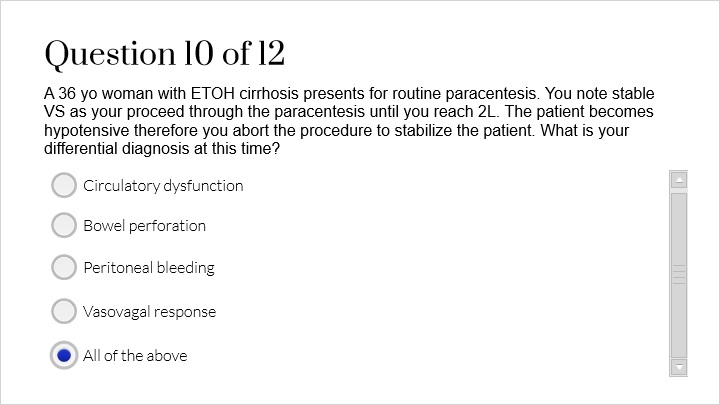


| Correct | Choice |
| --- | --- |
|  | Circulatory dysfunction |
|  | Bowel perforation |
|  | Peritoneal bleeding |
|  | Vasovagal response |
| X | All of the above |

**Feedback when correct:**

Circulatory dysfunction can occur at any time although we traditionally have higher concerns when removing > 5 L. One might expect vasovagal response earlier in the procedure especially in a patient naïve to paracentesis. Albumin may be of benefit in either situation.

Peritoneal bleeding is certainly a potential concern although would be of higher concern if there was a change in the fluid aspirated that might suggest aggressive bleeding. If persistent instability, ICU, interventional radiology, and surgical consults while stabilizing patient are warranted.

Bowel perforation should certainly be a concern and can be evaluated further with imaging (x-ray may potentially show free air given inherent to the procedure itself, recognize delays with oral contrasted CT scans, empiric antibiotic treatment).

**Feedback when incorrect:**

You did not select the correct response.


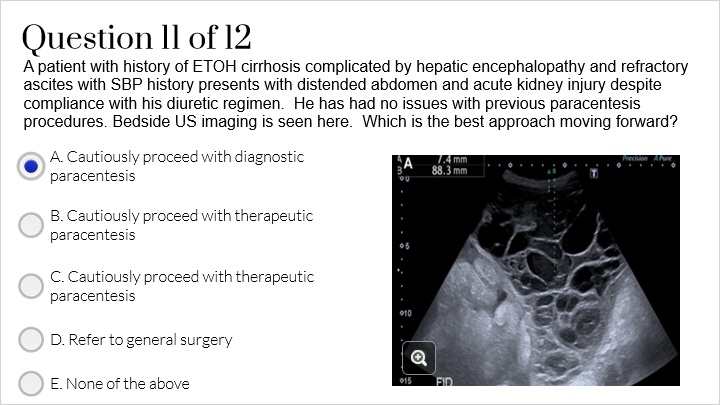


| Correct | Choice |
| --- | --- |
| X | A. Cautiously proceed with diagnostic paracentesis |
|  | B. Cautiously proceed with therapeutic paracentesis |
|  | C. Cautiously proceed with therapeutic paracentesis |
|  | D. Refer to general surgery |
|  | E. None of the above |

**Feedback when correct:**

This patient now presents with fairly complex fluid collection with significant loculations. Given his history, this is likely secondary to infection and should be further investigated. In this instance, therapeutic paracentesis is ineffective given the degree of loculations, but pursuing diagnostic study is still of value but caution should be taken to assure no vascularization is evident in the area targeted. Unfortunately, there are no studies that investigate the utility of more invasive interventions. No study demonstrates efficacy of prolonged antibiotics and this is felt to be poor given impairment of antibiotic diffusion. Few case reports note potential benefit of intra-abdominal fibrinolysis

**Feedback when incorrect:**

You did not select the correct response.


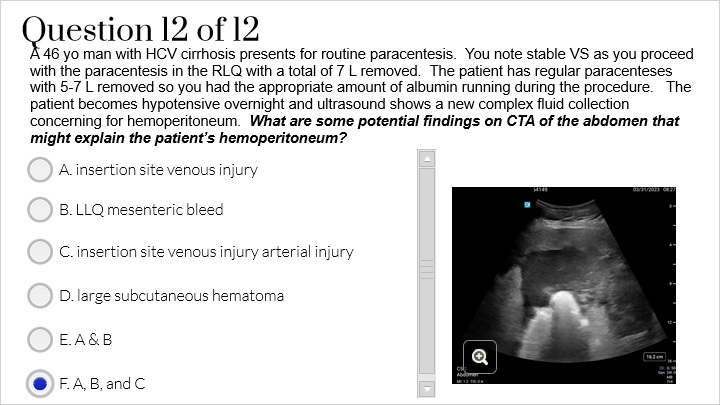


| Correct | Choice |
| --- | --- |
|  | A. insertion site venous injury |
|  | B. LLQ mesenteric bleed |
|  | C. insertion site venous injury arterial injury |
|  | D. large subcutaneous hematoma |
|  | E. A & B |
| X | F. A, B, and C |

**Feedback when correct:**

US can be of assistance to evaluate for hemoperitoneum and subcutaneous hematoma. In this case, there is no specific evidence of subcutaneous hematoma, but there is evidence of hemoperitoneum based on the new complex ascites evident. CTA is the next best test to further evaluate in order to assist interventional radiology in vessel targeting for embolization. This may identify a venous or arterial bleed along course of needle access but in rarer instance may isolate a mesenteric bleed either in proximity to procedure but for unknown reasons can occur outside the vicinity of the procedure.

**Feedback when incorrect:**

You did not select the correct response.


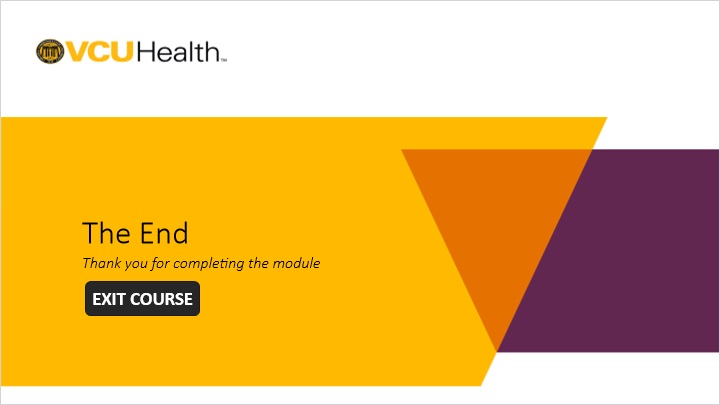

Supplement: Supplementary file 13 — Supplementary file13 (DOC 2.29 MB) [file 11606_2025_9677_MOESM13_ESM.doc]
