# Supplementary material for: Introducing a Comprehensive Framework for Competency-based Procedure Training
Source: J Gen Intern Med. 2025 Jul 8;40(15):3560–5. doi: 10.1007/s11606-025-09677-2 (PMC12612326; doi:10.1007/s11606-025-09677-2)
Supplement: Supplementary file 14 — Supplementary file14 (PDF 1.00 MB) [file 11606_2025_9677_MOESM14_ESM.pdf]

VCU Health

Knee Arthrocentesis

Learning Exchange Module

General Tenets of Procedures

Mastering procedure specific *knowledge*

Approaching each individual procedure and patient with the appropriate *attitude*

Developing specific procedure *skills*

VCU Health

2

Obtaining Informed Consent

5 Key Elements

1) Explanation of diagnosis or indication of proposed intervention
2) Description of intervention
3) Possible risks and benefits of proposed intervention
4) Possible alternatives and associated risks and benefits of alternative
5) Possible risks and benefits of not receiving proposed intervention

Be able to recognize situations where consent is deferred for emergent care or when surrogate decision maker is needed

VCU Health

3

Indications

Diagnostic
- Inflammatory
- Infectious
- hemarthrosis

Therapeutic
-corticosteroids

VCUHealth

4

Contraindications

Avoid overlying cellulitis
Suspected bacteremia unless septic arthritis suspected

Relative Contraindications:
coagulopathy

VCUHealth

5

Adverse Outcomes

Iatrogenic infection
Localized trauma
Pain
Reaccumulation of effusion

VCUHealth

6

Approaching Knee Arthrocentesis

Pre-procedure assessment
Procedure Specifics
- Understand anatomy
- Understand US
- Understand Equipment

Post-procedure course

Knee Arthrocentesis
Performance Checklist

Name		Date	
Training Program		Procedure/Site	
Training Year		Attending	

	Task (chronological Order)	Incompletely Performed	Completely Performed	Notes (Complete if not done at all or incompletely performed)
Pre-Procedure	1) Review patient's chart, labs, and imaging (as relevant)			
	2) Obtain informed consent			
	3) Position patient			
	4) Localize/mark needle insertion site by palpation or ultrasound			
	6) Wash hands and don personal protective equipment			
	7) Prepare site using chlorhexidine			
	8) Drape site using sterile technique			
	9) "time out": verify patient, procedure, and insertion site are correct			
	10) Utilize local anesthetic (1 % w/o epi) or topical spray			
Procedure	11) Insert needle			
	12) Stop advancement of needle once fluid is aspirated			
	13) Aspirate fluid			
	14) Withdraw needle			
Post-Procedure	18) Clean the area and apply dressing			
	19) Throw away sharps			
	20) Discard protective clothing			
	21) Wash hands			
	23) Document procedure and update nursing and primary team			

VCUHealth

Pre-procedure Assessment

Know your patient!
Conduct appropriate MSK exam
Medication review (anticoagulants, antiplatelets)
Lab review (coagulopathy, thrombocytopenia, uremia)
Prior imaging (plain films, US)
Prior procedure notes
Conduct bedside Ultrasound Survey

VCUHealth

Anatomy

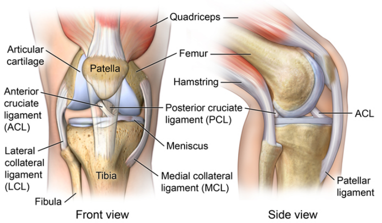

Knee anatomy

VCUHealth 9

Equipment

Sterile 25 gauge needle
Sterile 18 gauge needle
Sterile 30cc pr 60 cc syringe
Sterile 5cc syringe
1% Lidocaine without Epinephrine (or topical spray)
Sterile Gloves
Sterile Half-Drape/OR towels
Chloraprep (2)
Hairnet
Face Masks with Shield
Blood Culture Set (1)
Specimen Tubes
- purple top (Cell Count)
- specimen cup (GS,AFB)
- specimen cup (crystals)

Specimen Bags

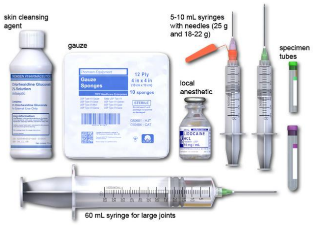

VCUHealth

10

Ultrasound Guided Procedure

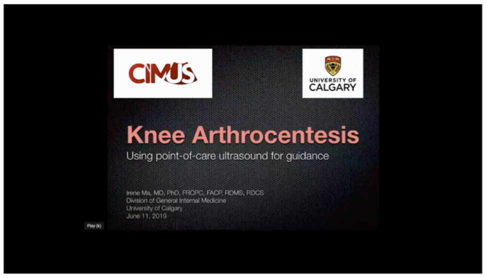

VCUHealth

Post-Procedure

- Remove PPE
- Dispose off sharps into appropriate sharps bin with sweep of patient bed to recover any sharps.
- Wash hands
- Document procedure and communicate with nursing and primary team

VCUHealth
