# Supplementary figures and images for: Introducing a Comprehensive Framework for Competency-based Procedure Training
Source: J Gen Intern Med. 2025 Jul 8;40(15):3560–5. doi: 10.1007/s11606-025-09677-2 (PMC12612326; doi:10.1007/s11606-025-09677-2)

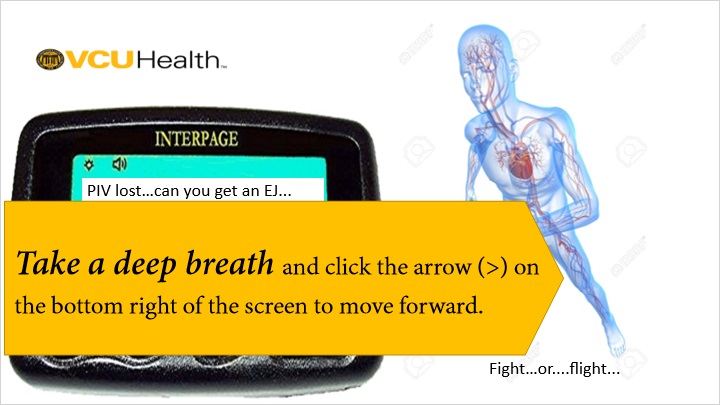


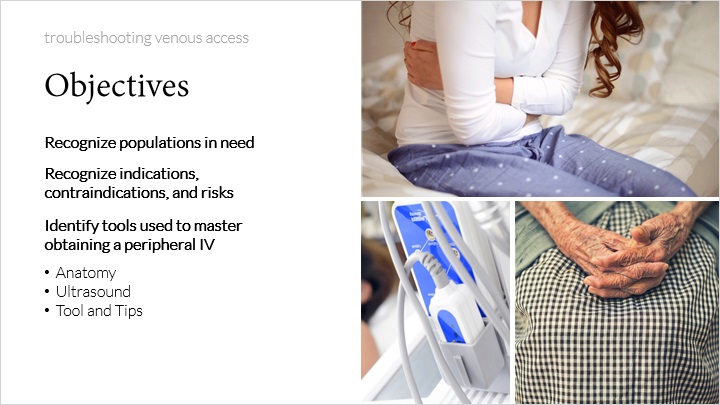


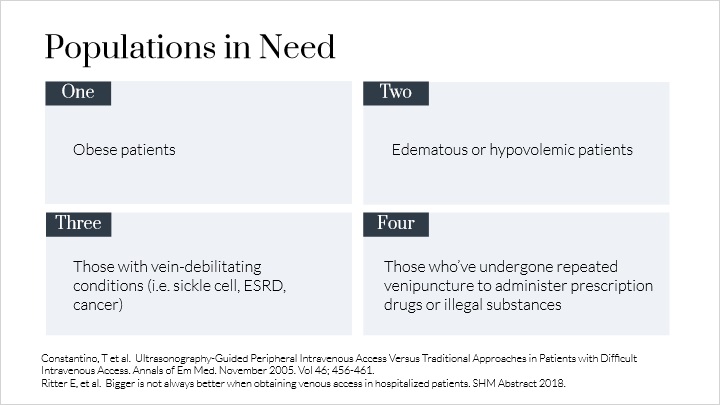


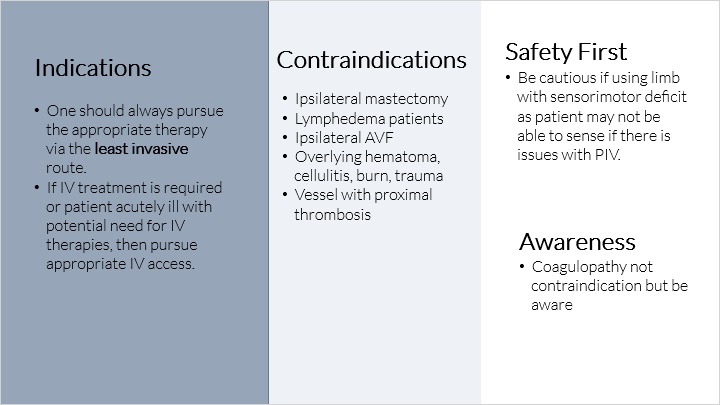


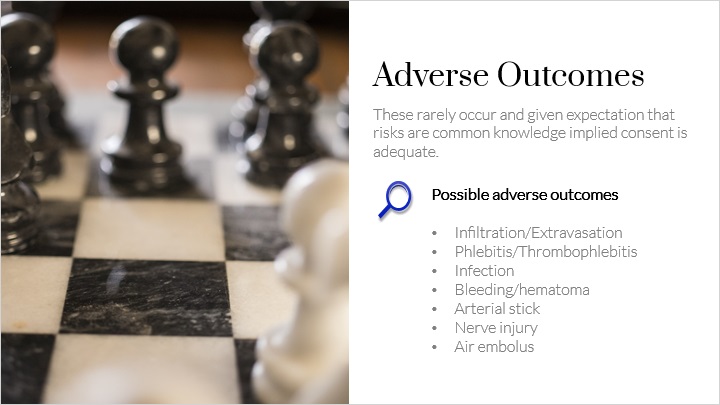


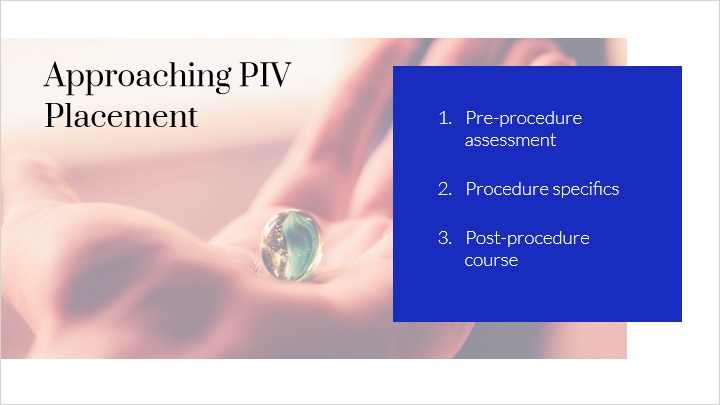


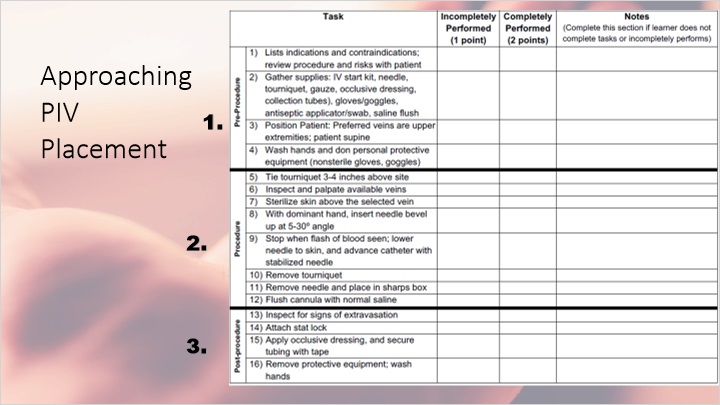


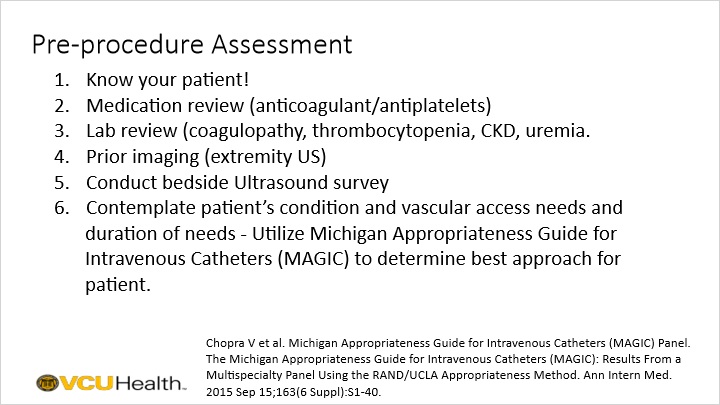


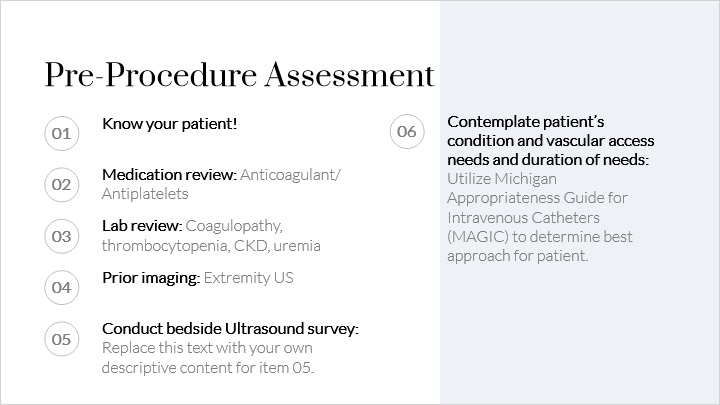


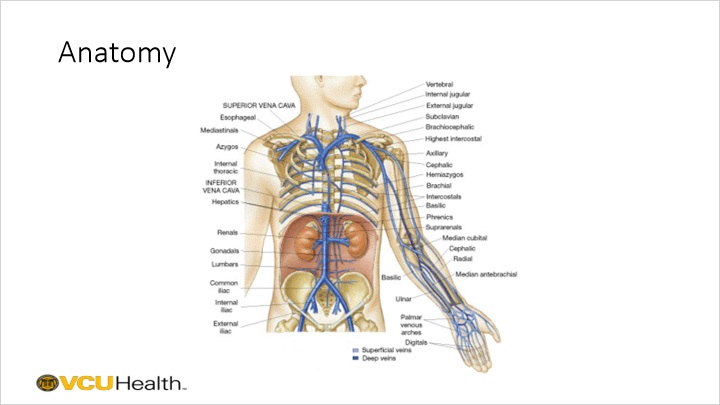


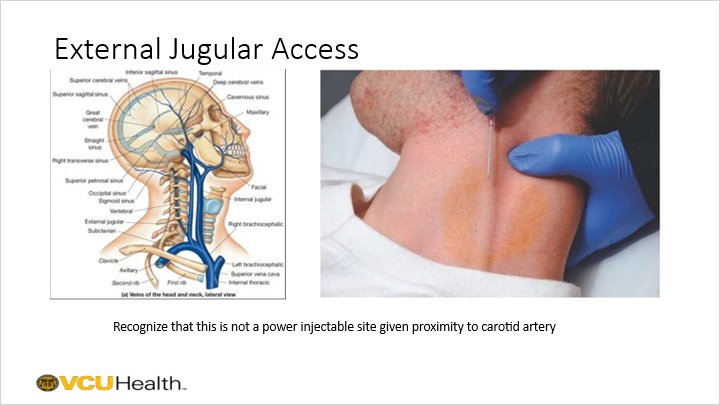


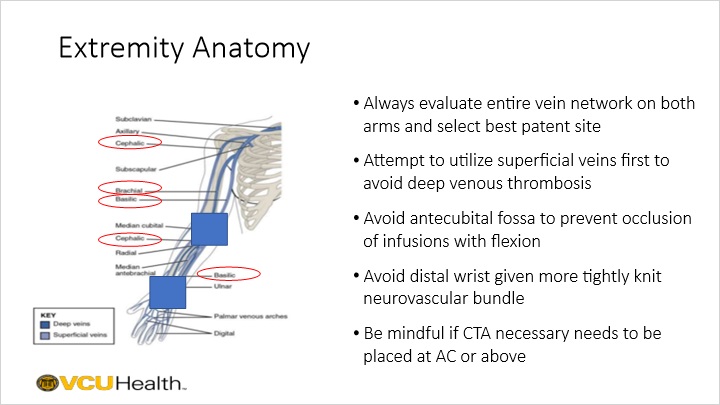


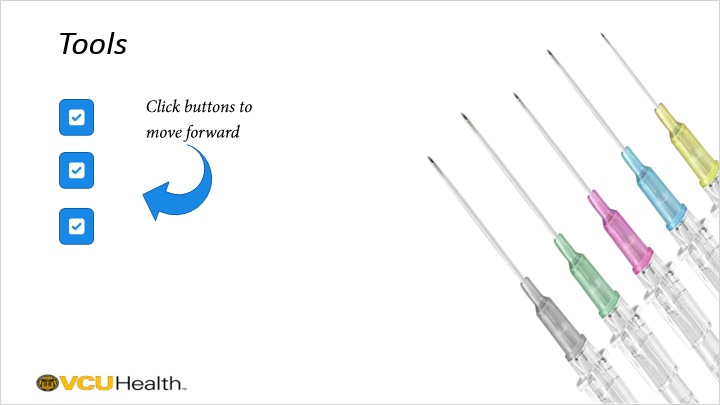


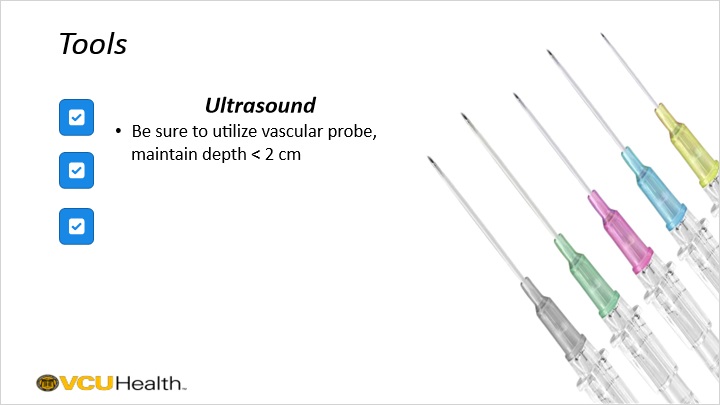


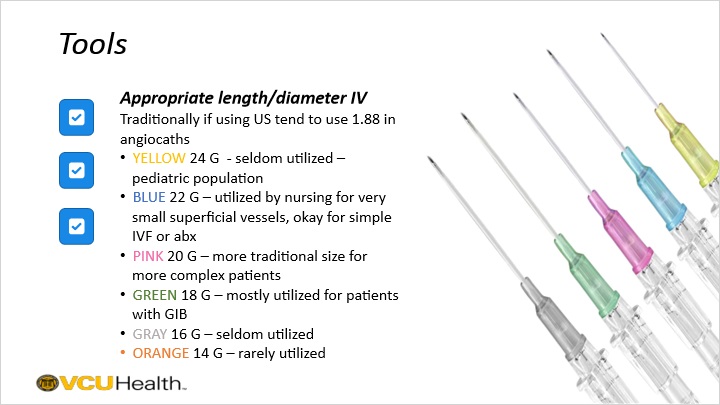


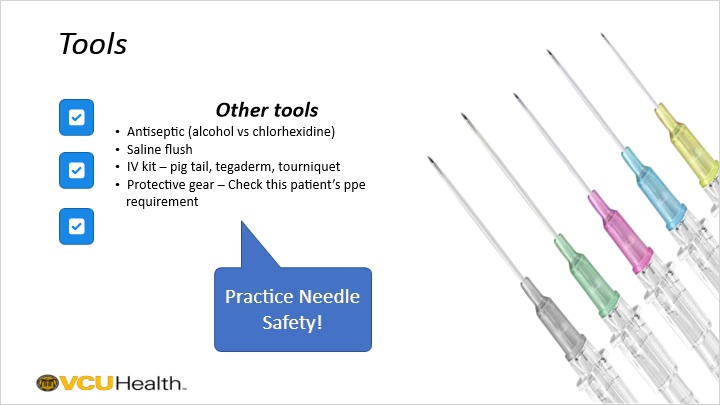


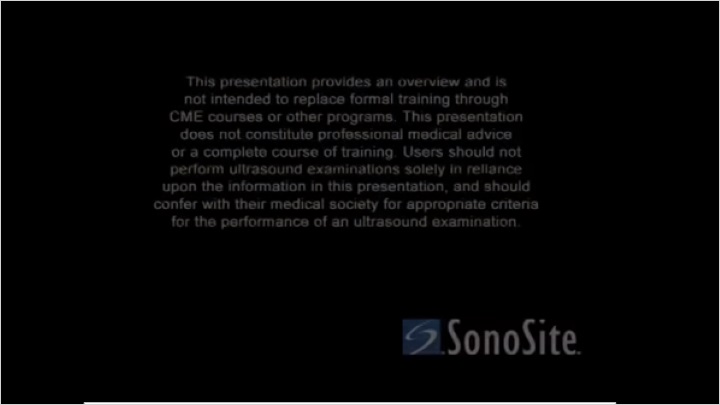


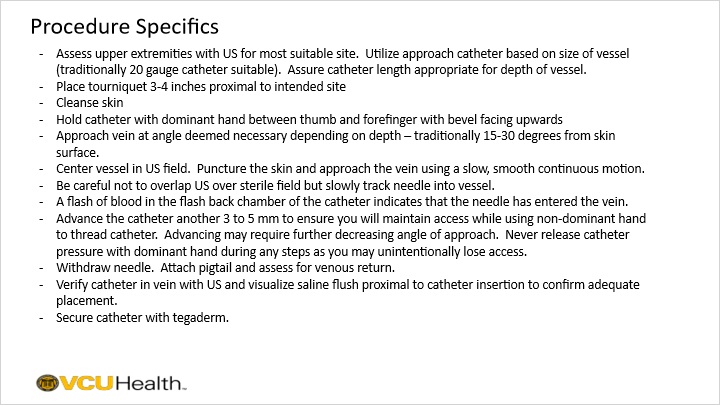


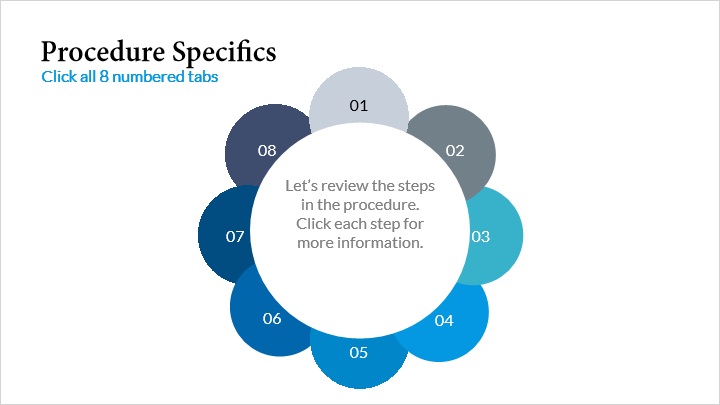


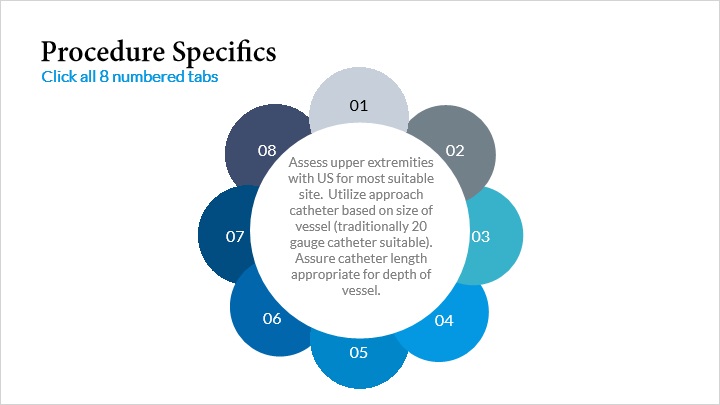


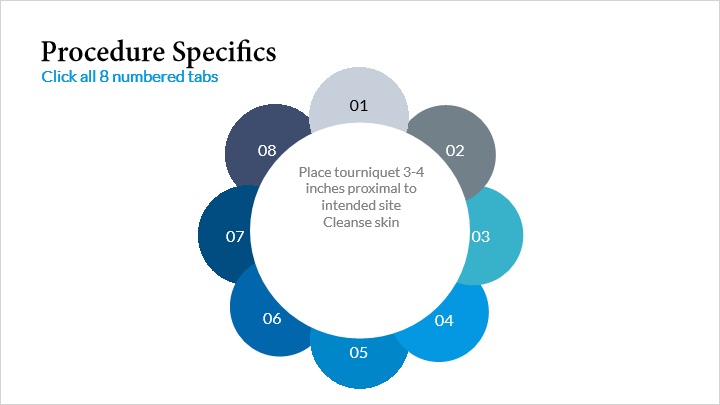


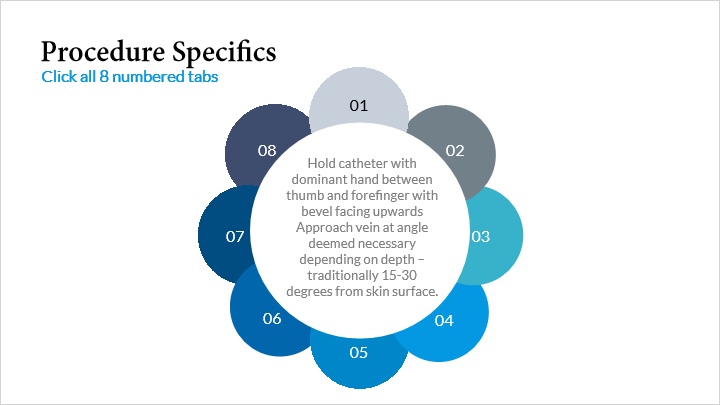


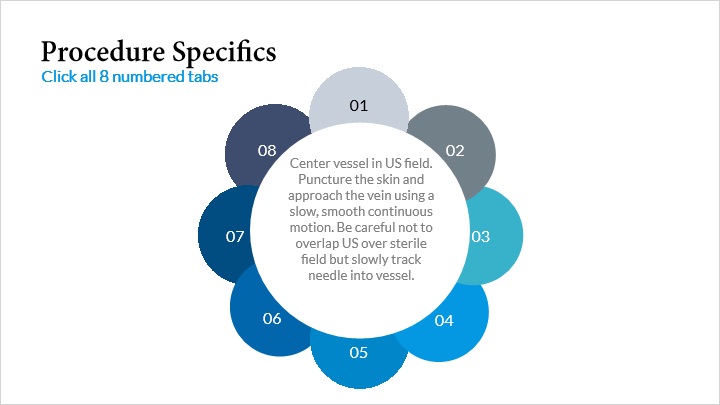


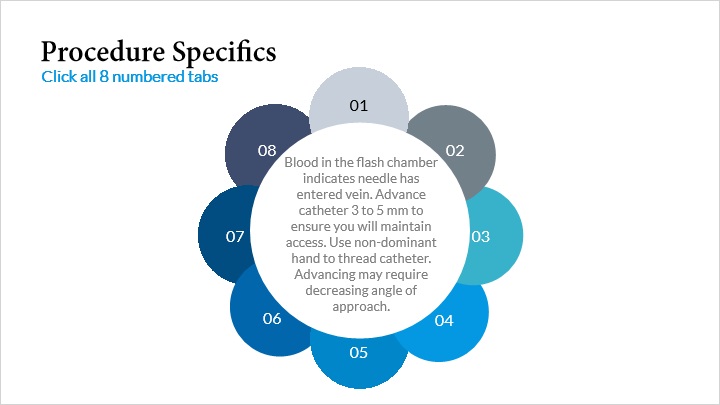


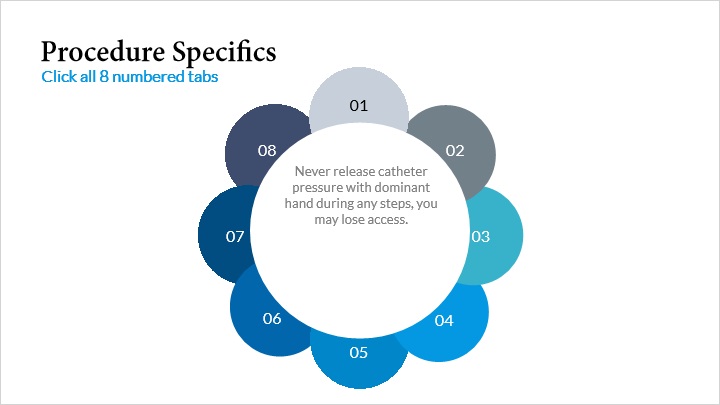


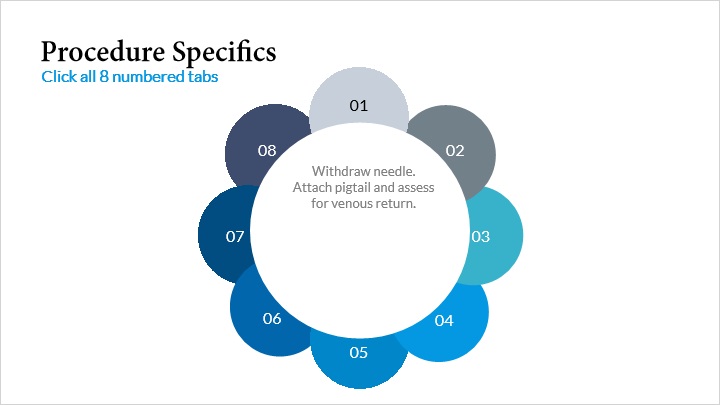


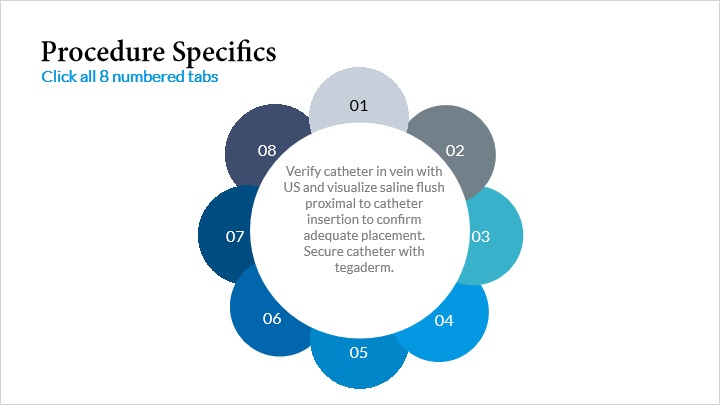


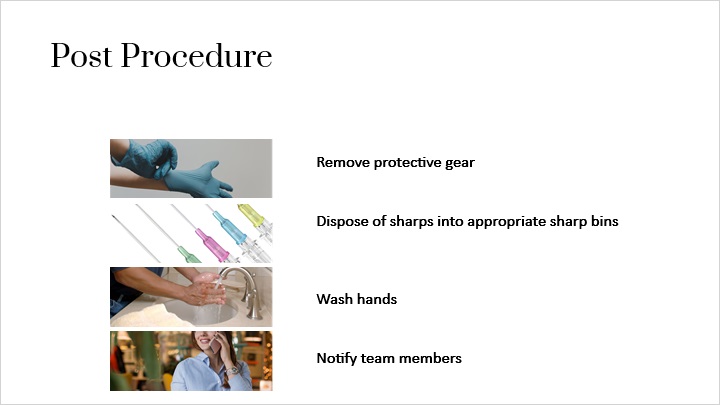


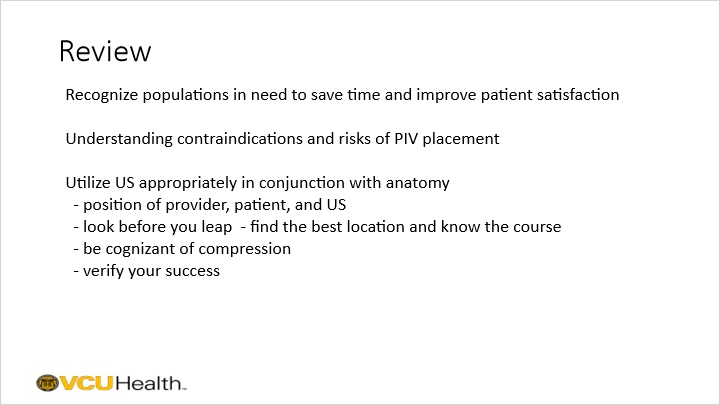


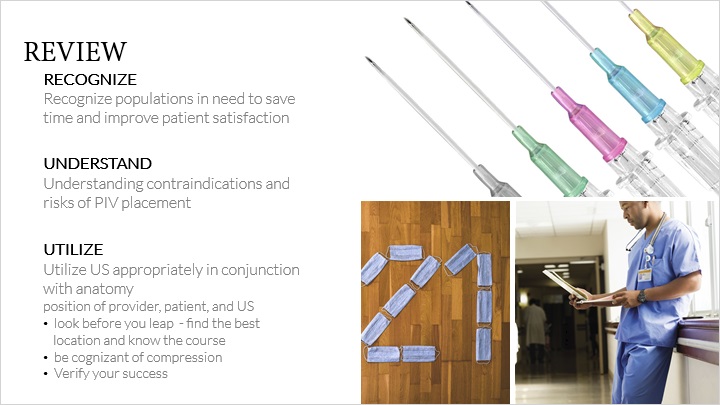


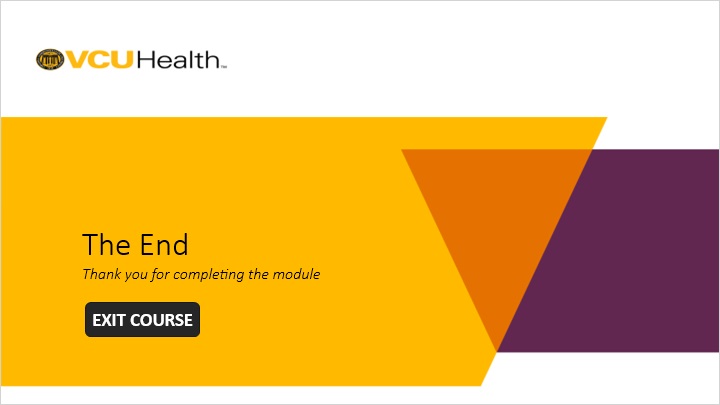

Supplement: Supplementary file 15 — Supplementary file15 (DOC 1.77 MB) [file 11606_2025_9677_MOESM15_ESM.doc]

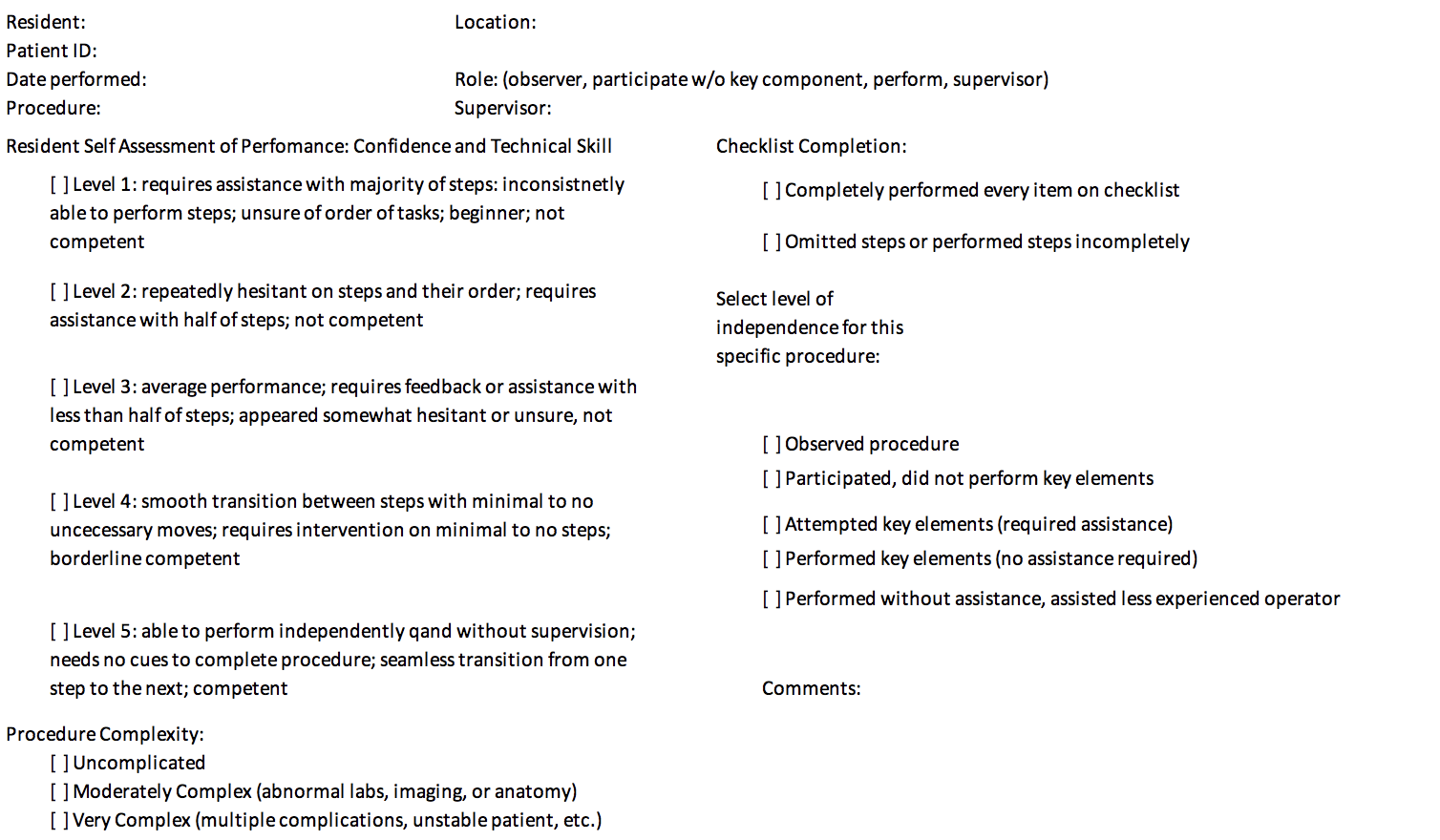

Supplement: Supplementary file 25 — Supplementary file25 (DOCX 185 KB) [file 11606_2025_9677_MOESM25_ESM.docx]

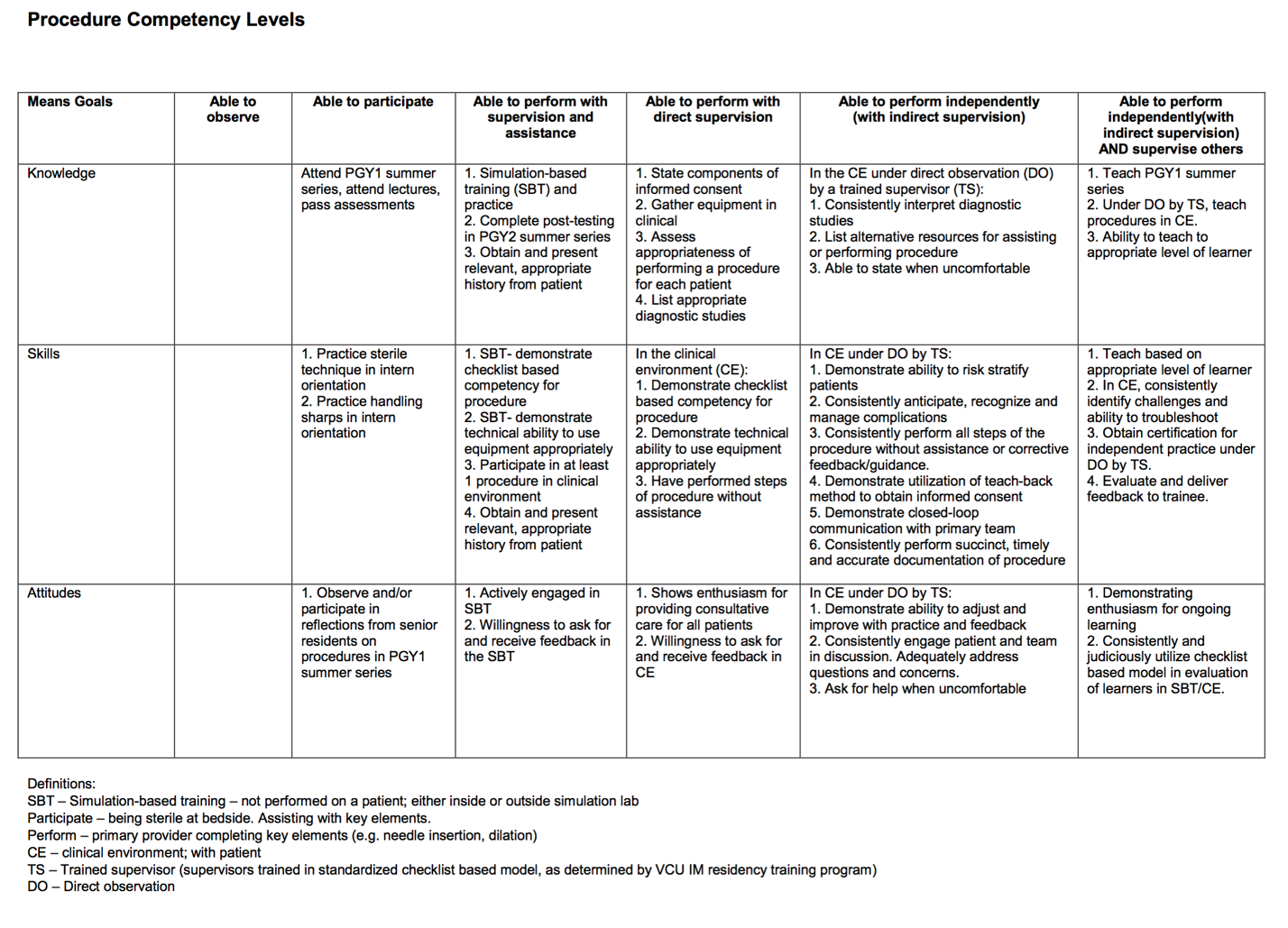


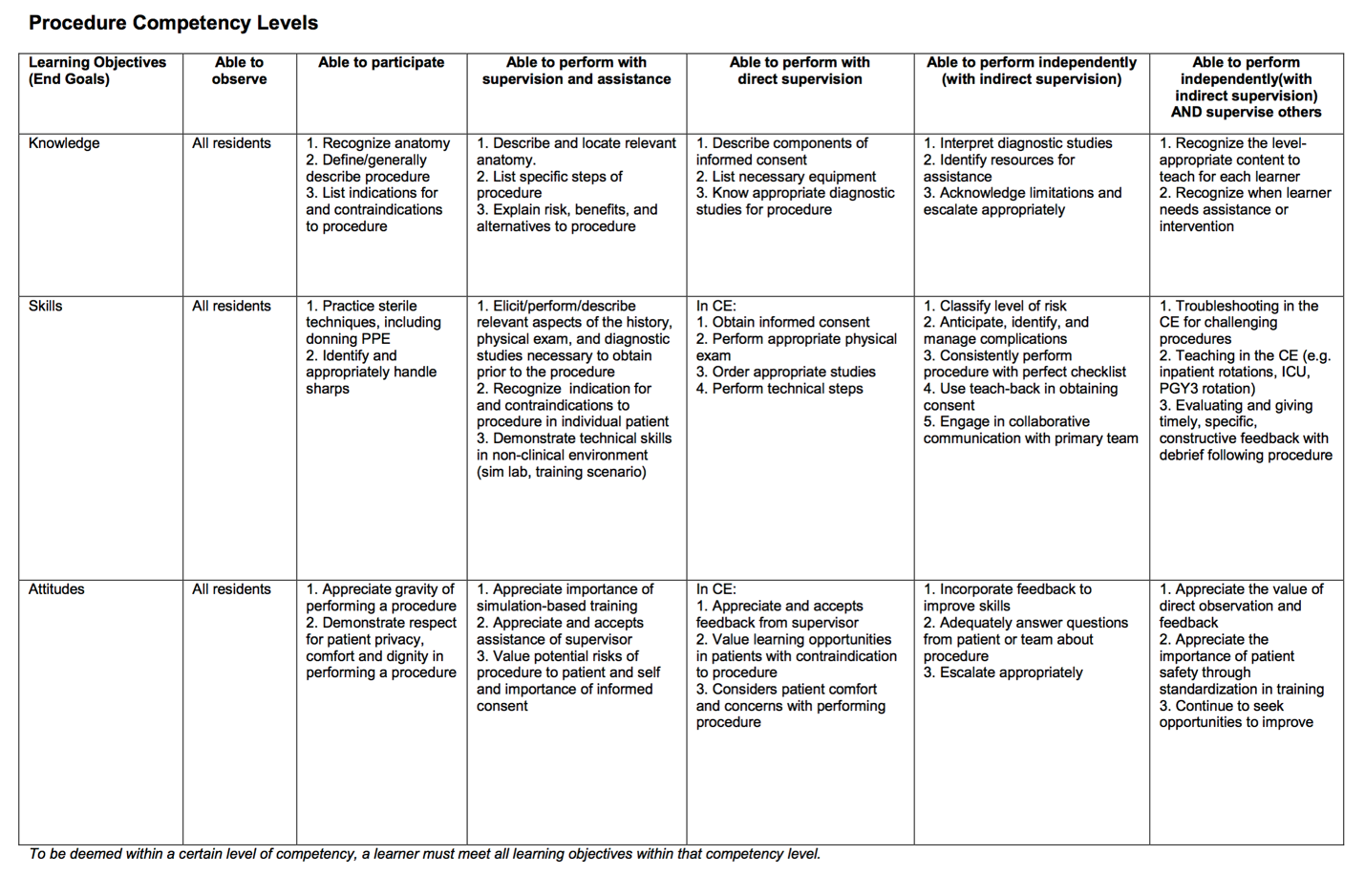

Supplement: Supplementary file 26 — Supplementary file26 (DOCX 1.37 MB) [file 11606_2025_9677_MOESM26_ESM.docx]
