# Supplementary material for: Introducing a Comprehensive Framework for Competency-based Procedure Training
Source: J Gen Intern Med. 2025 Jul 8;40(15):3560–5. doi: 10.1007/s11606-025-09677-2 (PMC12612326; doi:10.1007/s11606-025-09677-2)
Supplement: Supplementary file 16 — Supplementary file16 (PDF 3.45 MB) [file 11606_2025_9677_MOESM16_ESM.pdf]

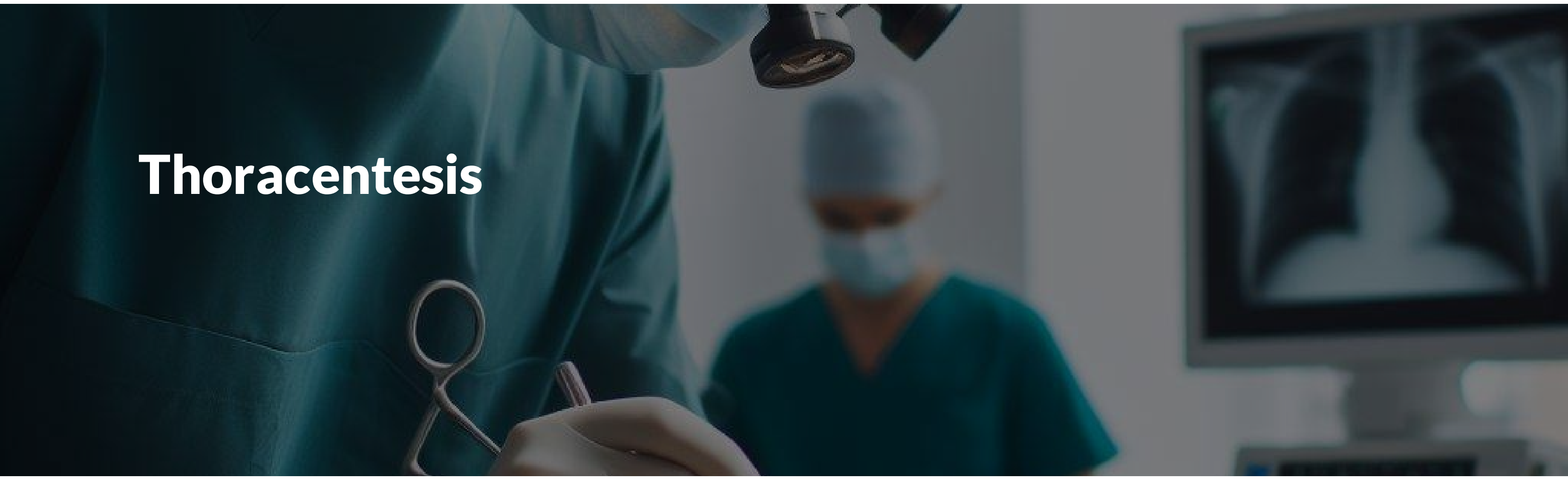

Thoracentesis

Introduction and Overview

Thoracentesis

Role of Ultrasound

Procedure

Test Your Knowledge

Introduction and Overview

General Tenets of Procedures

- Mastering procedure specific ***knowledge***
- Approaching each individual procedure and patient with the appropriate ***attitude***
- Developing specific procedure ***skills***

Obtaining Informed Consent

There are 5 key elements of obtaining informed consent.

1. Explanation of diagnosis or indication of proposed intervention
2. Description of intervention
3. Possible risks and benefits of proposed intervention
4. Possible alternatives and associated risks and benefits of alternative
5. Possible risks and benefits of not receiving proposed intervention

Be able to recognize situations where consent is deferred for emergent care or when surrogate decision maker is needed

Indications

Therapeutic

- To relieve symptoms in patient with dyspnea/hypoxia with large pleural effusion

Diagnostic

Transudative

- Congestive Heart Failure
- Cirrhosis
- Nephrotic Syndrome

Exudative

- Parapneumonic and empyema

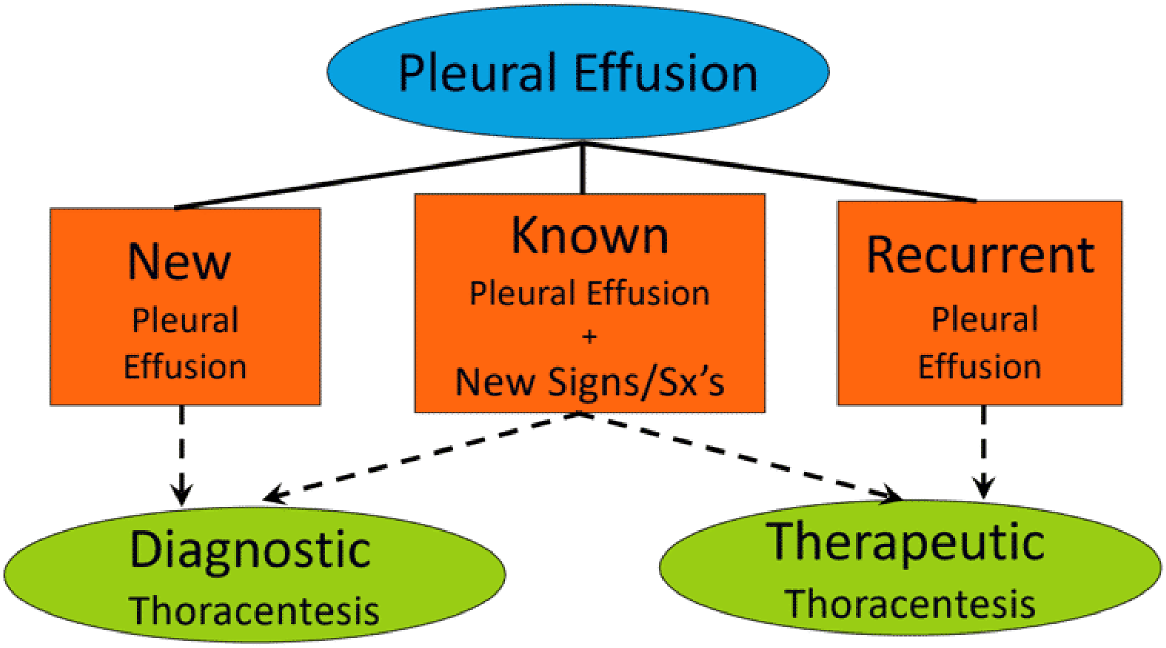

Contraindictions

Absolute

- Overlying cellulitis, shingles at insertion site

Relative

- Coagulopathies
- PLT <50,000
- Altered chest wall anatomy
- Mechanical or manual ventilation
- Pulmonary disease severe enough to make complications life threatening
- Uncooperative patient

Adverse Outcomes

- Pain or cough
- Bleeding - hemothorax vs hematoma (<1%)
- Infection (2%)
- Pneumothorax (~6%)
- Re-expansion pulmonary edema (<.25%)
- Liver or spleen puncture (<.05%)
- Diaphragm Injury (<.05%)
- Vasovagal episode (<.06%)
- Subcutaneous emphysema

Complications of thoracentesis: incidence, risk factors, and strategies for prevention

Click the 'GO TO ARTICLE' button to access this article by Cantey, E. P. et al.

GO TO ARTICLE

Pneumothorax often not severe (<1 % requiring intervention) but if results in increased resp effort, hypoxia, then chest tube insertion is likely necessary

Pneumothorax and hemothorax worsen mortality, morbidity, and extend LOS

CONTINUE

Thoracentesis

Approaching Thoracentesis

Pre-procedure assessment

Procedure Specifics

- Understand anatomy
- Understand US
- Understand Equipment

Post-procedure course

Task (chronological Order)		Incompletely Performed	Completely Performed	Notes (Complete if not done at all or incompletely performed)
Pre-Procedure	1) Review Patient's chart, labs, and imaging (as relevant)			
	2) Use POCUS to determine the size and nature of the effusion (Is an effusion present? Simple vs complex? Loculated?)			
	3) Obtain informed consent: verify patient, procedure, and site			
	4) Gather supplies: thoracentesis kit, etc			
	5) Position patient:<br>a) posterior approach – standard upright position with arms lifted to help move scapula out of the way<br>b) lateral approach – recumbent position with arms lifted above the head (if patient unable to sit up for any specific reason)			
	6) Use US to identify site<br>a) phased array probe – to assure adequate pocket to not injure lung and/or diaphragm<br>b) linear probe with color power doppler to identity any vessels in needle trajectory site			
	7) Wash hands and don personal protective equipment (sterile gown and sterile gloves, eye protection)			
	8) Prepare site using chlorhexidine			
	9) Drape site using sterile technique			
	10) "time out": verify patient, procedure, and insertion site are correct			
Procedure	11) Prepare and inject anesthetic in trajectory identified earlier			
	12) Prepare the kit: assemble the needle/catheter device, and tubing			
	13) Insert needle perpendicular to skin superior to inferior rib to avoid neuromuscular bundle			
	14) Slowly advance the needle with dominant hand and control depth with non-dominant hand closer to skin while constantly applying negative pressure to skin			
	15) Advance needle 5 mm after fluid is aspirated to assure catheter has entered pleural space			
	16) Stabilize the needle and advance catheter over the needle			
	17) Withdraw the needle			
	18) Using 60 cc syringe, aspirate fluid for diagnostic studies			
	19) Aspirate for therapeutic purpose – connect Y-shaped tubing to 60 cc syringe and manually withdraw fluid<br>a) stop removing fluid if 1) no more output, 2) if patient becomes symptomatic – severe coughing, increased SOB, chest pain, 3) if manometry > -20 cm H20			
	20) Stop suction prior to removing the catheter			
Post-Procedure	21) Clean the area, ensure no bleeding, and apply dressing			
	22) Throw away sharps			
	23) Discard protective clothing			
	24) Wash hands			
	25) Properly label specimens			
	26) Document procedure and update nursing and primary team			

Thoracentesis: State-of-the-Art in Procedural Safety, Patient Outcomes, and Physiologic Impact

Click the 'GO TO ARTICLE' button to access this article by DeBiasi EM, Puchalski J.

GO TO ARTICLE

Pre-procedure Assessment

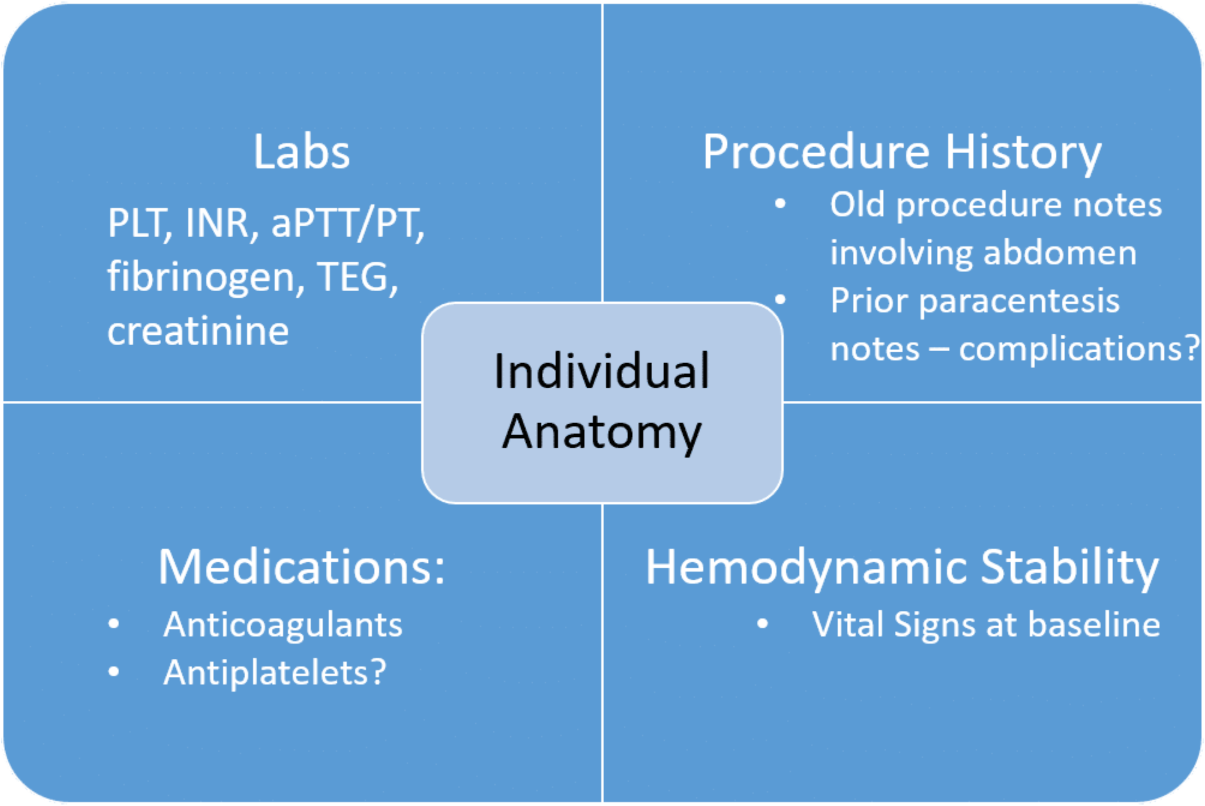

Coagulopathy in Thoracentesis

TABLE 1. Summary of Periprocedural Management of Coagulopathy for Paracentesis, Thoracentesis, and Lumbar Puncture, Stratified by Referenced Guidelines

		Paracentesis and thoracentesis	Lumbar puncture
		Correction threshold	
INR	Without chronic liver disease	≤2.0-3.0[2]<br><2.0[3]<br><1.5[5a]	≤2.0-3.0[2]<br>≤1.5[4]<br><1.5[3]
	With chronic liver disease	No threshold[2,6b]<br><2.0[3]	No threshold[2]<br>≤1.5[4]<br><1.5[3]
Platelets (per µL)		No threshold[6b]<br>>20,000[2,3]<br>>50,000[5a]	≥20,000[2]<br>>40,000[3,7c]<br>≥50,000[4]

[a]Specific to thoracentesis.
[b]Specific to paracentesis in the setting of chronic liver disease.
[c]If platelets are 20,000-40,000/µL, an additional risk-benefit discussion is encouraged.
Abbreviation: INR, international normalized ratio.

Consensus Guidelines are based on expert opinion and there are variations in recommendations.

Given this, take all variables and patient factors into account when assessing bleeding risk.

Clinical Progress Note: Consolidated Guidelines on Management of Coagulopathy and Antithrombotic Agents for Common Bedside Procedures Click the 'GO TO ARTICLE'

button to access this article by Blank, J.A., Peters, K.K., O'Donnell, M.A. and Mansoor, A.M.

GO TO ARTICLE

Medication Considerations

Click tables below to enlarge information.

TABLE 2. **Summary of Periprocedural Management of Antiplatelet and Anticoagulant Agents for Paracentesis, Thoracentesis, and Lumbar Puncture, Stratified by Referenced Guidelines**

	Paracentesis and thoracentesis		Lumbar puncture	
	Recommended hold time prior to procedure[a]	Recommended restart time after procedure[a,b]	Recommended hold time prior to procedure[a]	Recommended restart time after procedure[a,b]
Aspirin	Continue[2,5c]		Continue[2]<br>Continue (if low dose)[4,7]<br>5 d (if high dose)[4]	No delay[7]
Clopidogrel	Continue[2,5c]		Continue[2]<br>5 d[4]<br>5-7 d[8]<br>7 d[7,9]	After 6 h[7]<br>After 12-24 h[9]<br>Next day[4]
Prasugrel	Continue[2,5c]	Not applicable	Continue[2]<br>7 d[7]<br>7-10 d[8,9]	After 6 h[7]<br>After 24 h[9]
Ticagrelor	Continue[2,5c]		Continue[2]<br>5 d[9]<br>5-7 d[8]<br>7 d[7]	After 6 h[7]<br>After 24 h[9]
Dipyridamole	Continue[2]		Continue[2,9]<br>24 h[7,8]	After 6 h[7,8]
Abciximab	24 h[2]	Not available	24 h[2,4]<br>24-48 h[8]<br>48 h[7]<br>2-5 d[9]	After 8 h[4]<br>After 8-12 h[9]<br>After 24 h[7]
Tirofiban, eptifibatide	4-8 h[2]		4 h[4]<br>4-8 h[2,7,8]<br>8-24 h[9]	After 8-12 h[9]<br>After 24 h[7]
Warfarin	Continue[10]<br>Continue if no patient-related risk factors for bleeding[11de]<br>Continue with INR within patient's target range[3f]<br>Hold to target INR ≤3.0[2]<br>3-5 d to target INR <2.0[3g]<br>5 d to target INR <1.5[5c]	After 12-24 h[3]<br>After 24 h[11]	Hold to target INR ≤3.0[2]<br>3-5 d to target INR ≤1.5[4]<br>5 d[2,3,7] to target INR <1.5[3] or ≤1.4[7]<br>5 d to target a normal INR[8,9]	After 6 h[9]<br>After 12 h[4,7]<br>After 12-24 h[7]<br>After 24 h[11]
LMWH				
Prophylactic dosing	Continue[3f]<br>12 h[3g]	After 6 h[3]	6-12 h[3]<br>12 h[7-9]	After 4 h[7]<br>After 6-12 h[3]<br>After 12 h[8]<br>After 12-24 h[9]
Therapeutic dosing	One dose[5c]<br>24 h[3g]	After 6-12 h[3]	24 h[3,7-9]	After 4 h (after 24 h if traumatic)[7]<br>After 12-24 h[9]<br>After 24-72 h[3]
Either	Continue[2,10]	Not applicable	Continue[2]<br>Last dose or 12 h[4]	After 24 h[4]

	Paracentesis and thoracentesis	
	Recommended hold time prior to procedure[a]	Recommended restart time after procedure[a,b]
Unfractionated heparin infusion	Continue[2,10]<br>4 h[3]<br>4-6 h[5c]	After 6 h[3]
Fondaparinux		
Prophylactic dosing	Continue[3f]<br>36 h[3g]	After 6-12 h[3]
Therapeutic dosing	48 h[3]	After 6-12 h[3]
Either	Continue[2,10]<br>24 h[5c]	Not applicable<br>Not available
Argatroban	Continue[2,10]<br>4 h[3]<br>48 h[5c]	After 6 h[3]
Dabigatran	Continue[2,3f,10]<br>≥24 h[11d]<br>24-48 h[3g]<br>≥48 h[5c,11c]	After 6 h[3]<br>Next day[11d]<br>After 48-72 h[11c]
Rivaroxaban	Continue[2,3f,10]<br>≥24 h[5c,11d]<br>≥48 h[3g,11c]	After 6 h[3]<br>Next day[11d]<br>After 48-72 h[11c]
Apixaban	Continue[2,3f,10]<br>≥24 h[5c,11d]<br>24-48 h[3g]<br>≥48 h[11c]	After 6 h[3]<br>Next day[11d]<br>After 48-72 h[11c]

- (a) For patients with clinically significant renal dysfunction, please see individual references for periprocedural hold and restart times.
- (b) assuming post procedure hemostasis has been achieved.
- (c) Specific for thoracentesis.
- (d) specific for paracentesis.

- (e) Patients specific bleeding risk factors include, but are not limited to, hypertension, abnormal renal function, abnormal hepatic function, prior stroke, history of major bleeding(especially within the preceding 3 months), and bleeding history with a similar procedure.
- (f) For patients with high risk thrombosis, consider continuing anticoagulation.
- (g) For patients with low risk of thrombosis, consider either continuing anticoagulation or holding anticoagulation prior to the procedure for he duration listed.

Anatomy

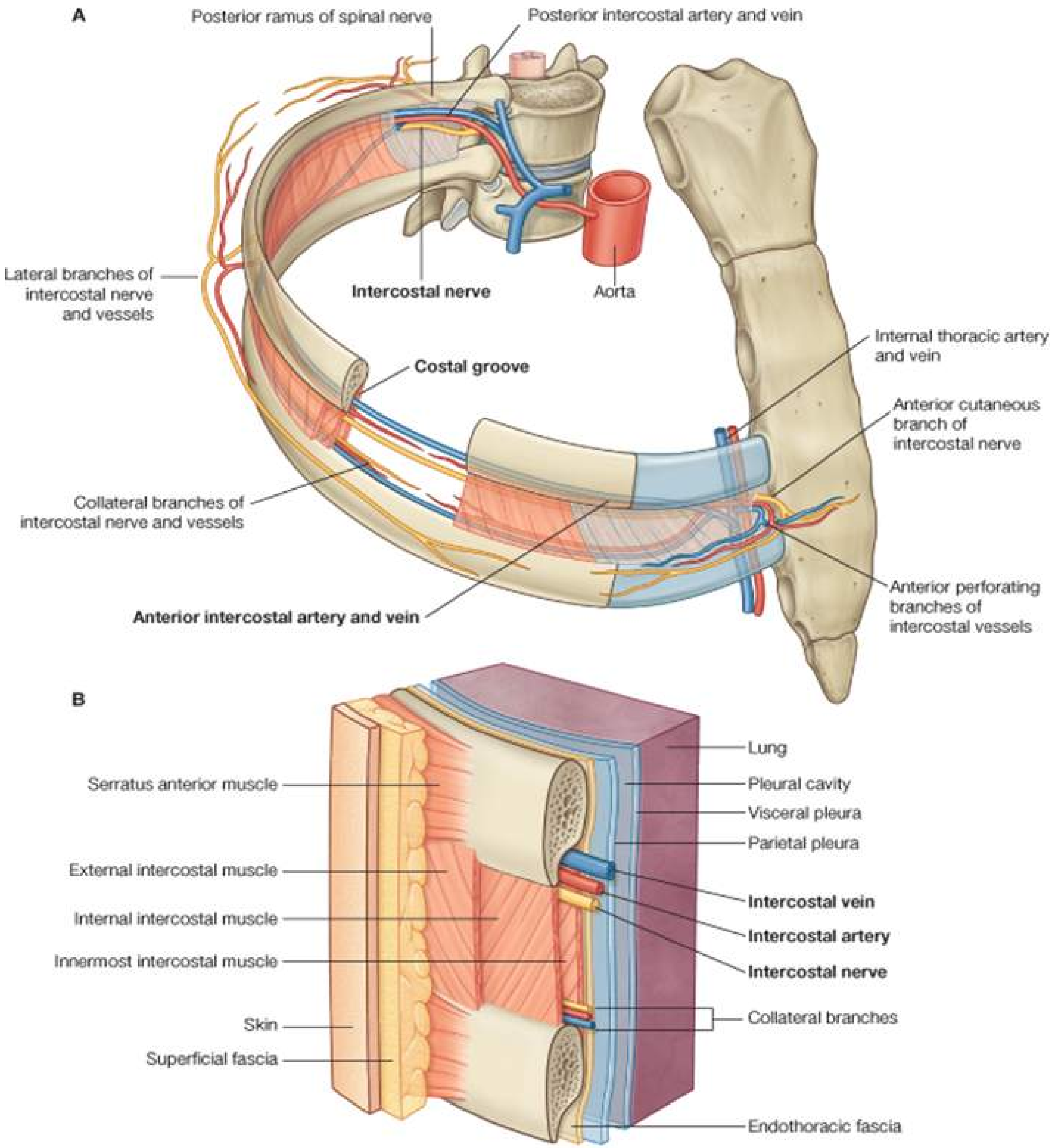

© Elsevier. Drake et al: Gray's Anatomy for Students - www.studentconsult.com

Thoracentesis

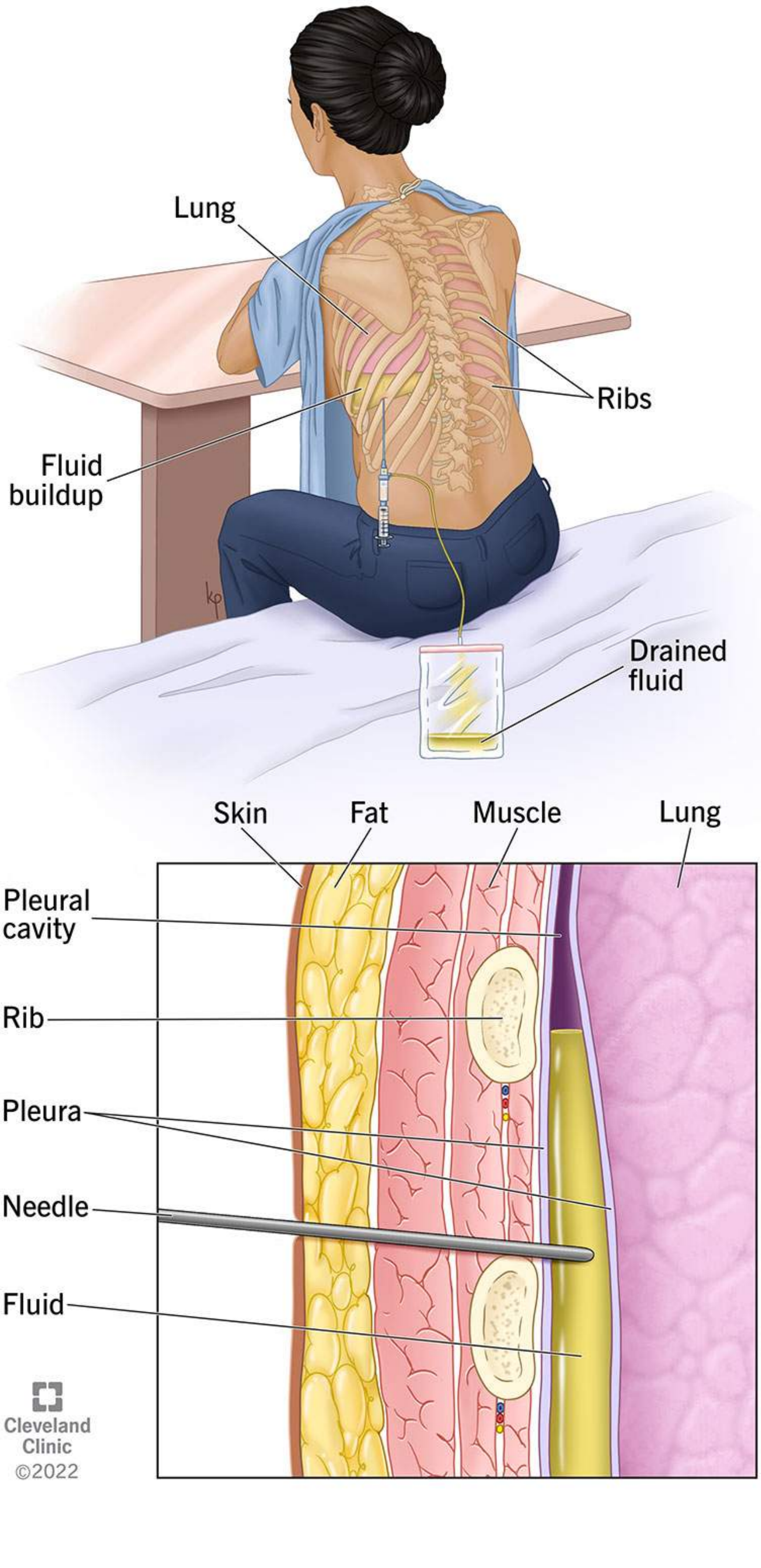

Cleveland Clinic
©2022

CONTINUE

Role of Ultrasound

Role of Ultrasound

- **Evaluate presence of fluid and can estimate volume**
- **Evaluate characteristics of fluid**
  - Simple fluid vs complex fluid
  - Loculations/septations
- **Evaluate thoracic wall**
  - Predict depth of fluid pocket
  - Evaluate for vessels
  - Evaluate for sliding lung

Two-probe Technique

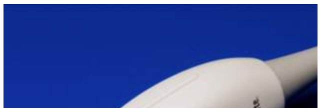

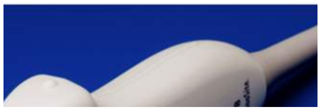

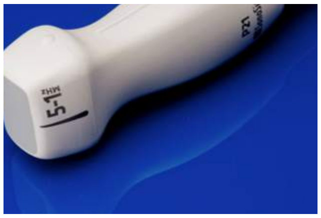

- Pleural Effusions

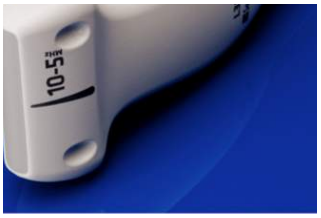

- Pleural Lesions
- Peripheral Lung Lesions

Role of Ultrasound: Classic Sonographic Signs

	Pleural Effusion PRESENT	Pleural Effusion ABSENT
**Curtain Sign**	–	+
**Mirror Image**	–	+
**Spine Sign**	+	–

CONTINUE

Curtain Sign

Fig. 1

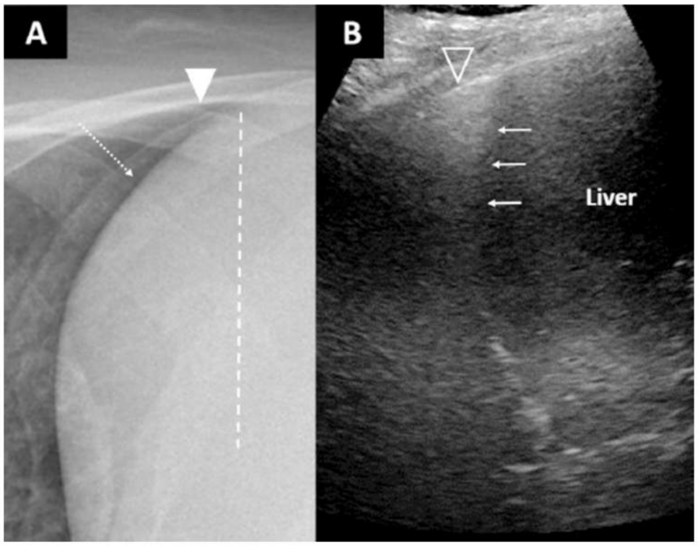

Formation of normal curtain sign. A. CXR illustration of the overlap of costophrenic recess (arrowhead) over the abdomen. In U/S, the air in the costophrenic recess will cast an "air curtain" (dotted line), hiding the lateral diaphragm (dotted arrow) B. Ultrasound appearance of the normal CS (thin arrows), which moves with respiration and cover the lateral diaphragm throughout all phases of the respiratory cycle. The open arrow head denotes the lowest limit of the pleural line at the level of the costophrenic recess.

The Curtain Sign in Lung Ultrasound

Click the 'GO TO ARTICLE' button to access this article by Lee FCY.

GO TO ARTICLE

Mirror Image

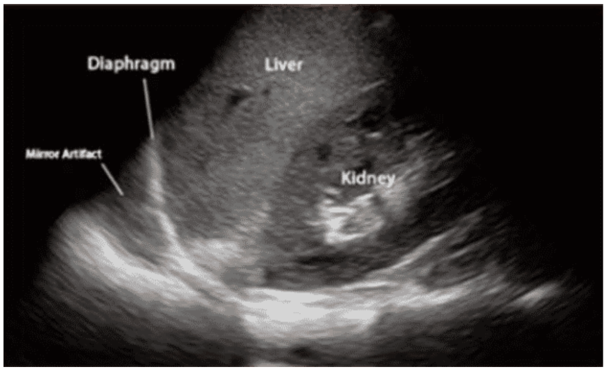

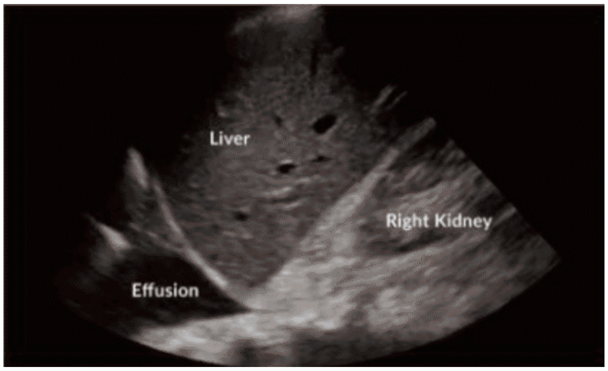

Mirror artifact occurs when there is a strong difference in tissue velocity. When reflective echoes bounce back and forth, the structure is flipped. Mirror artifacts are friendly artifacts that let us exclude pleural effusion because of reflection of the liver's image through the diaphragm.

Spine Sign

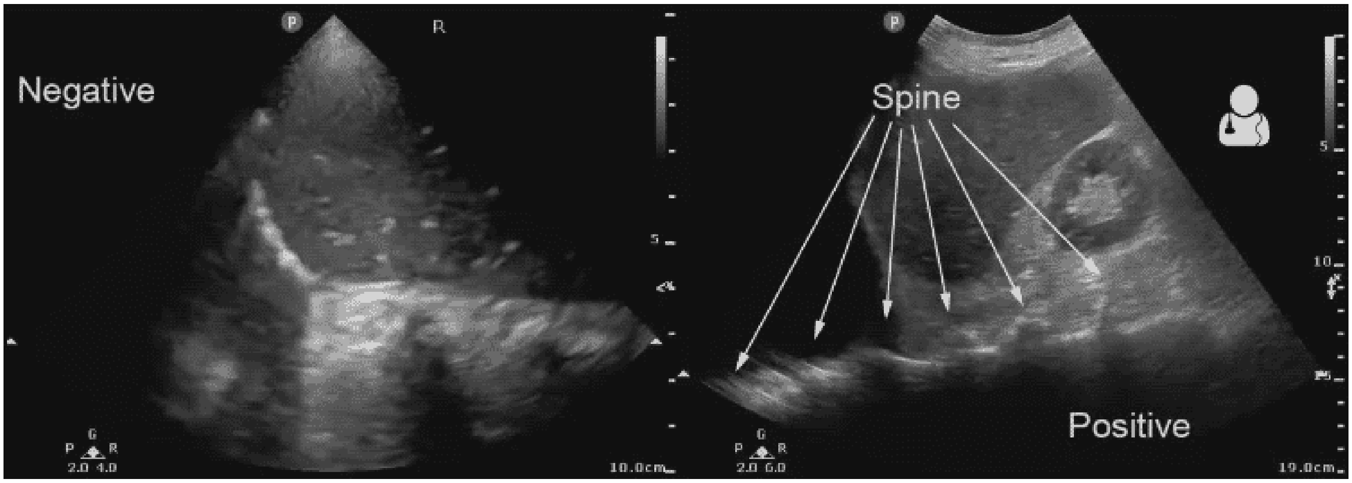

In patients without a pleural effusion the spine is obscured by air in the lung, so the spine is cut off at the diaphragm. In patients with a pleural effusion the spine is visible beyond the diaphragm.

Lung Ultrasonographic Signs in Pulmonary Disease – A Video Review.

Click the 'GO TO ARTICLE' button to access this article by Dugar S, Fox S, Koratala A, Moghekar A, Mehta AC

GO TO ARTICLE

Role of Ultrasound: Volume

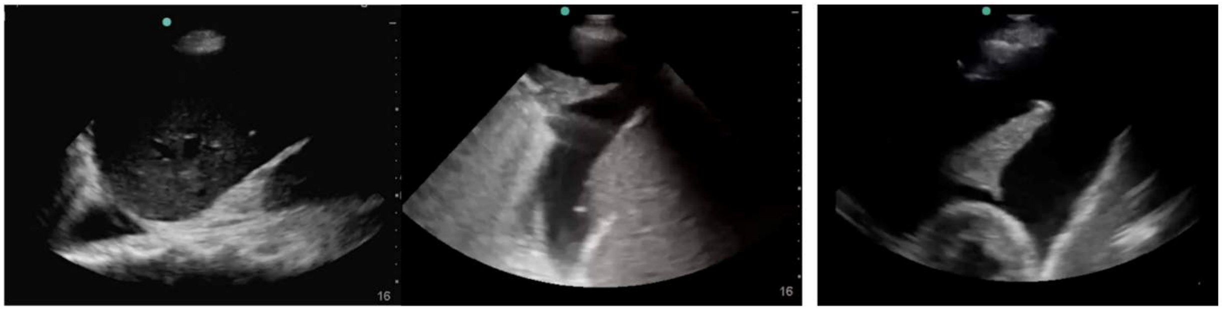

Small | Moderate | Large

Role of Ultrasound: Character

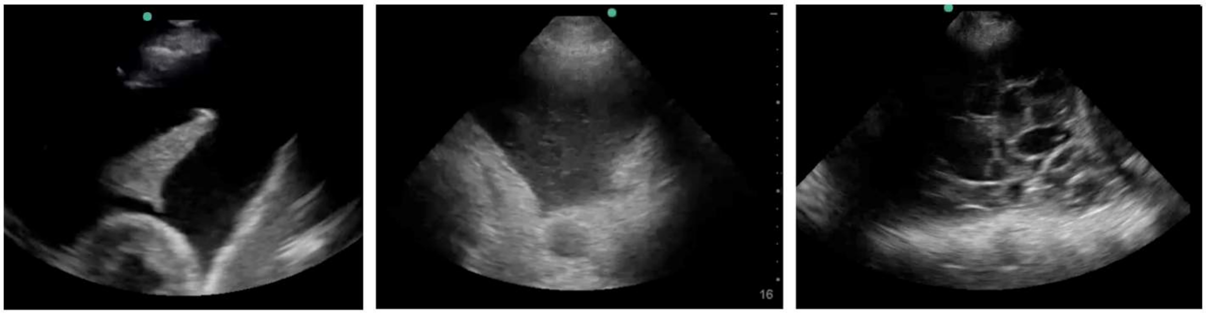

Simple | Complex | Complex Septated

Ultrasound is better than CT at assessing effusions.

Exudative findings:

- Plankton ”sign” – floating debris
- Increased echogenicity
- Fibrinous stranding and septations

CONTINUE

Procedure

Procedure: Positioning

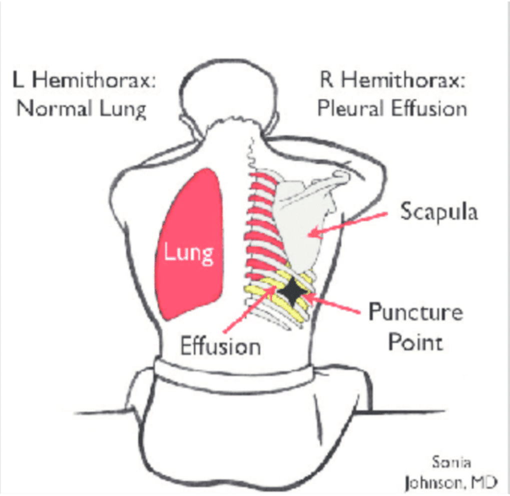

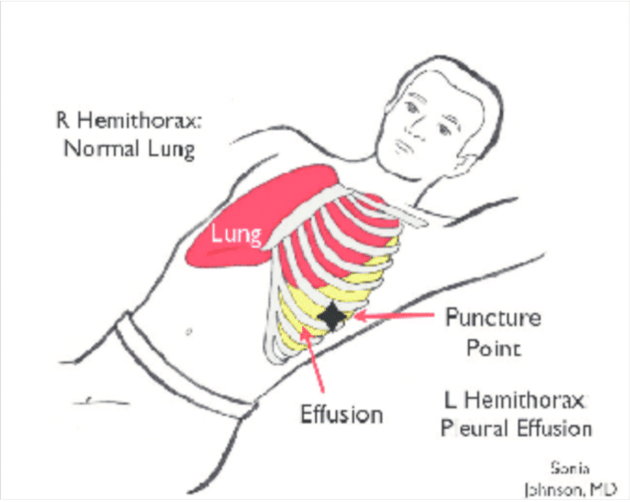

Semi upright – proceed with operator comfort

Procedure: Site Selection

Step 1

Procedure: Site Selection

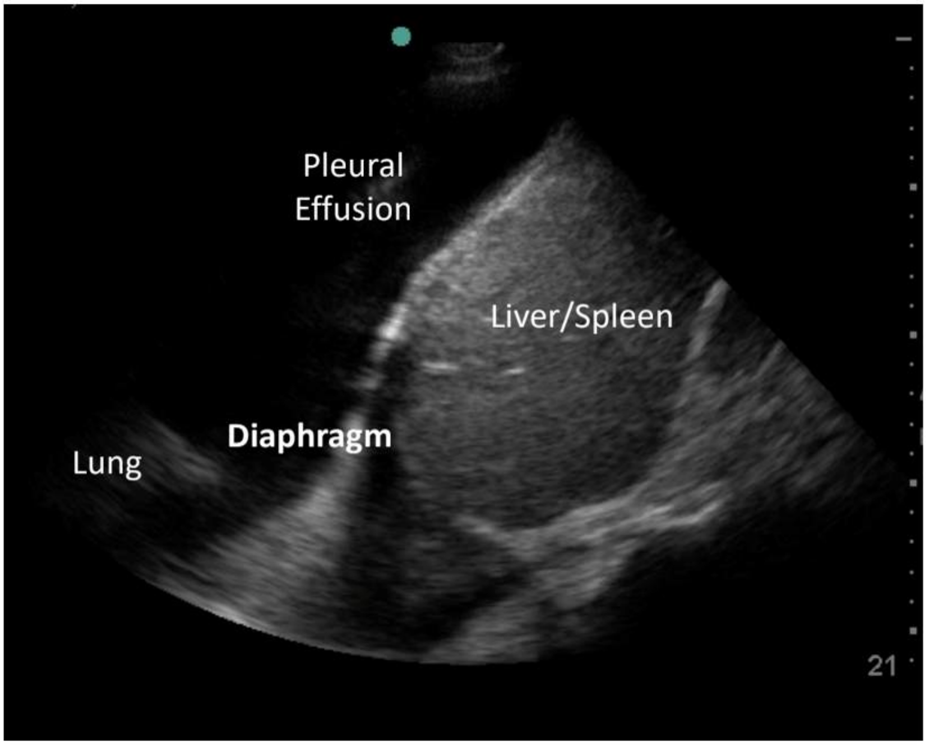

1. Identify Critical structures (lung, diaphragm, liver/spleen)

Step 2

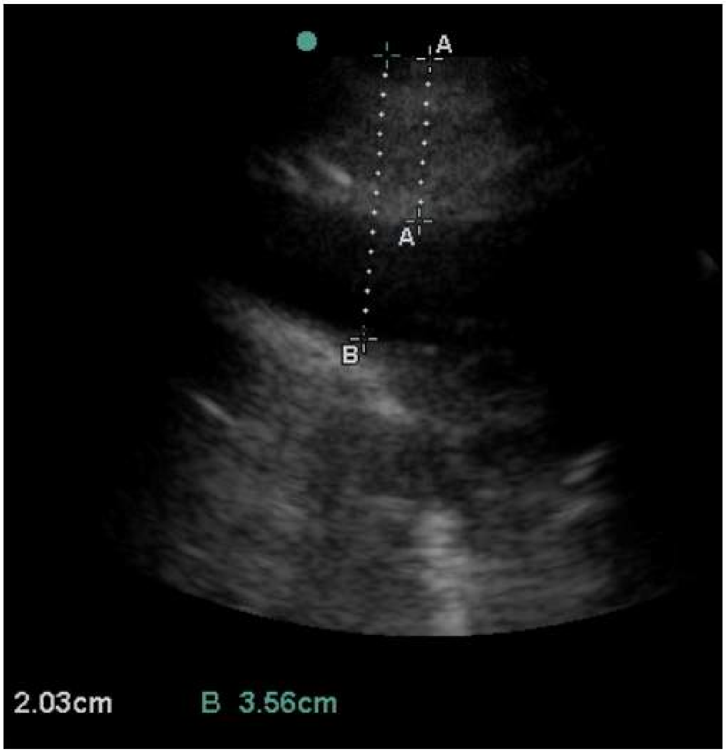

2. Find most accessible fluid collection

- Largest fluid collection with thinnest chest wall (> than 2 cm in each of three dimensions).

Step 3

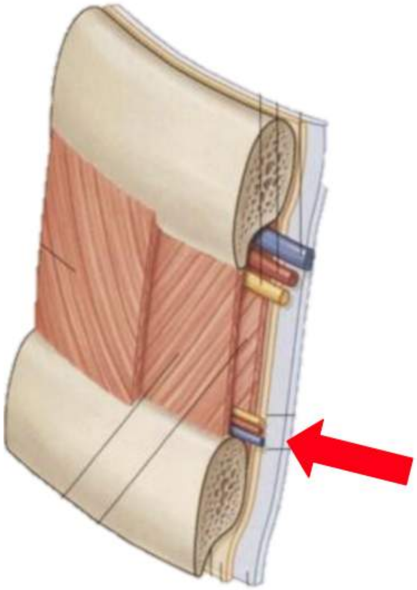

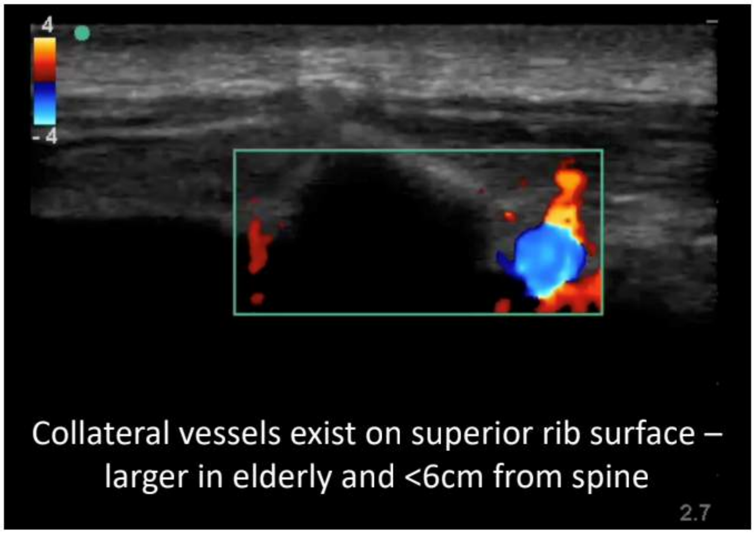

3. Measure depth of subcutaneous tissue and rule out blood vessels along trajectory

Procedure: Site Selection

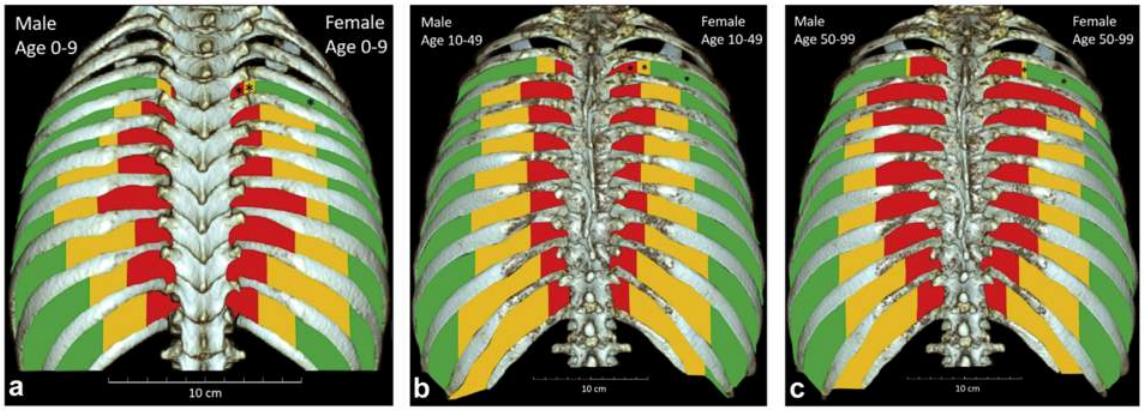

Figure 2. The theoretical high-risk, moderate-risk, and low-risk zones for thoracic interventions hugging the lower rib (red, yellow, and green, respectively), stratified by intercostal space level, sex, and age range. (a) 0–9 years, (b) 10–49 years, and (c) 50–99 years. Not enough intercostal arteries were visible in the third intercostal space in women for calculating the cutoff points; extrapolations are indicated with an asterisk.

Seated - > 10 cm from spine ideal to avoid vessels

Misura, Tihana & Drakopoulos, Dionysios & Mitrakovic, Milena & Loennfors, Tarja & Primetis, Elias & Hoppe, Hanno & Obmann, Verena & Huber, Adrian & Ebner, Lukas & Christe, Andreas. (2022). Avoiding the Intercostal Arteries in Percutaneous Thoracic Interventions. Journal of Vascular and Interventional Radiology. 33. 416-419.e2. 10.1016/j.jvir.2021.12.026.

Click here to access this journal article.

Procedure

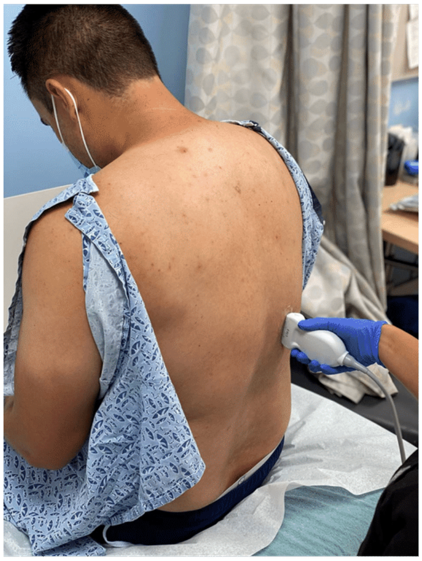

Task (chronological Order)		Incompletely Performed	Completely Performed	Notes (Complete if not done at all or incompletely performed)
Pre-Procedure	1) Review Patient's chart, labs, and imaging (as relevant)			
	2) Use POCUS to determine the size and nature of the effusion<br>(Is an effusion present? Simple vs complex? Loculated?)			
	3) Obtain informed consent: verify patient, procedure, and site			
	4) Gather supplies: thoracentesis kit, etc			
	5) Position patient:<br>a) posterior approach – standard upright position with arms lifted to help move scapula out of the way<br>b) lateral approach – recumbent position with arms lifted above the head (if patient unable to sit up for any specific reason)			
	6) Use US to identify site<br>a) phased array probe – to assure adequate pocket to not injure lung and/or diaphragm<br>b) linear probe with color power doppler to identity any vessels in needle trajectory site			
	7) Wash hands and don personal protective equipment (sterile gown and sterile gloves, eye protection)			
	8) Prepare site using chlorhexidine			
	9) Drape site using sterile technique			
	10) "time out": verify patient, procedure, and insertion site are correct			
Procedure	11) Prepare and inject anesthetic in trajectory identified earlier			
	12) Prepare the kit: assemble the needle/catheter device, and tubing			
	13) Insert needle perpendicular to skin superior to inferior rib to avoid neuromuscular bundle			
	14) Slowly advance the needle with dominant hand and control depth with non-dominant hand closer to skin while constantly applying negative pressure to skin			
	15) Advance needle 5 mm after fluid is aspirated to assure catheter has entered pleural space			
	16) Stabilize the needle and advance catheter over the needle			
	17) Withdraw the needle			
	18) Using 60 cc syringe, aspirate fluid for diagnostic studies			
	19) Aspirate for therapeutic purpose – connect Y-shaped tubing to 60 cc syringe and manually withdraw fluid<br>a) stop removing fluid if 1) no more output, 2) if patient becomes symptomatic – severe coughing, increased SOB, chest pain, 3) if manometry > -20 cm H20			
	20) Stop suction prior to removing the catheter			
Post-Procedure	21) Clean the area, ensure no bleeding, and apply dressing			
	22) Throw away sharps			
	23) Discard protective clothing			
	24) Wash hands			
	25) Properly label specimens			
	26) Document procedure and update nursing and primary team			

Procedure: Equipment

- Straight Needle
    - 16-gauge
    - 22-gauge (diagnostic only)
    - Spinal needle
- Valved needle-catheter systems
    - Turkel™ safety needle
    - Safe-T-Centesis™
- Non-valved needle-catheter systems
    - Centeze® catheter
    - Yueh needle

**No large, randomized trials comparing needle types*

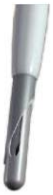

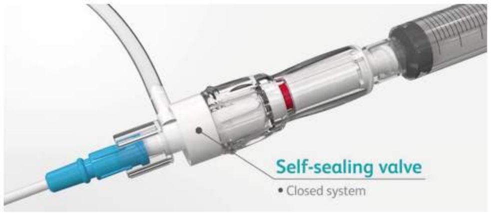

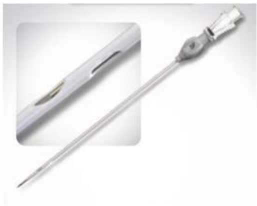

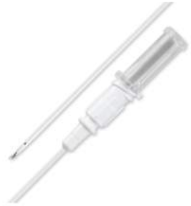

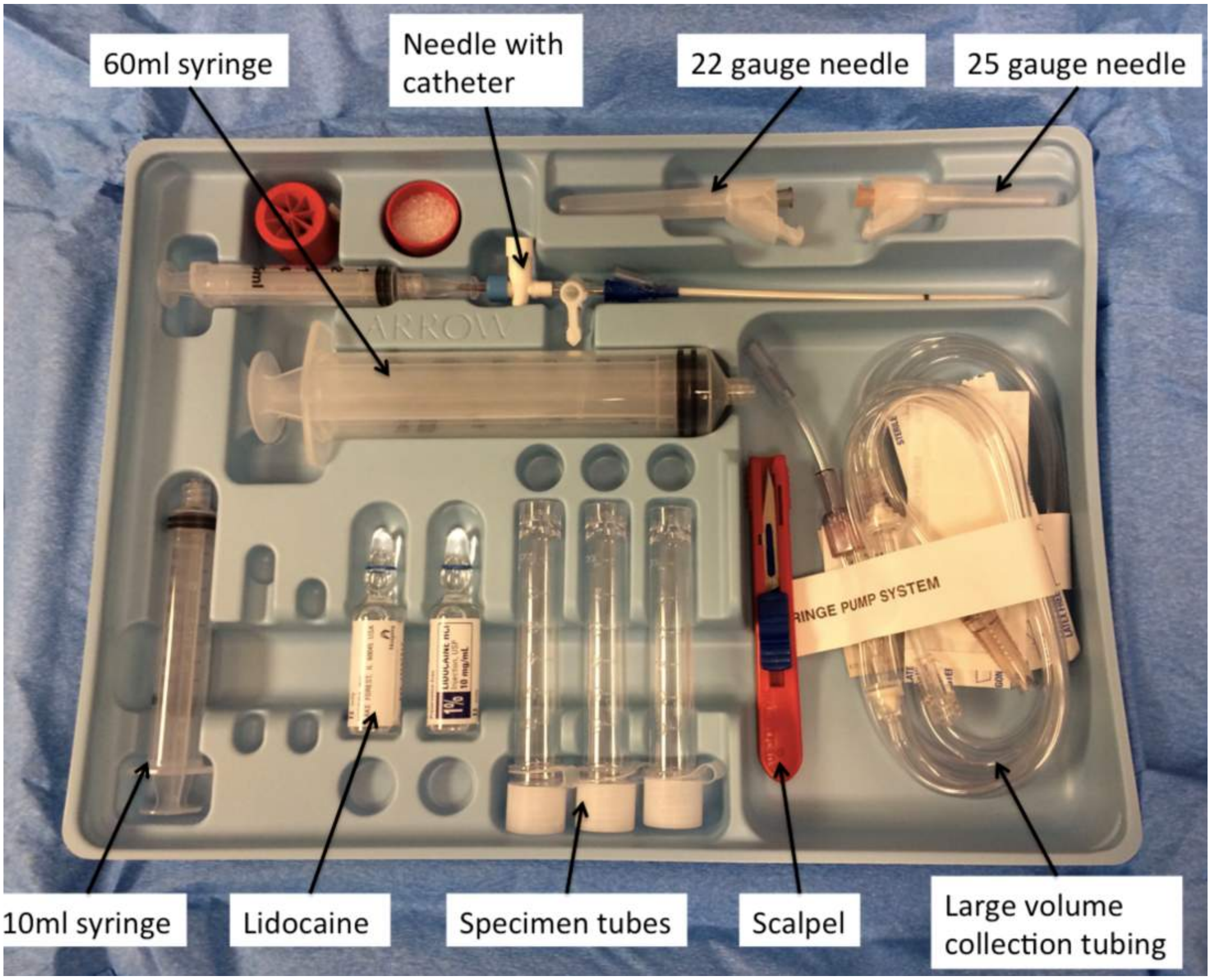

Procedure Video

Thoracentesis

UT Health
San Antonio
Hospital Medicine

This program is an asset of The University of Texas Health Science Center at San Antonio.

POST-PROCEDURE	INTRA-OP AND POST-OP CHALLENGES	CONTROVERSIES

- Remove PPE
- Dispose of sharps into appropriate sharps bin with sweep of patient bed to recover any sharps.
- Verify no pneumothorax with imaging
- Wash hands
- Document procedure and communicate with nursing and primary team

POST-PROCEDURE | INTRA-OP AND POST-OP CHALLENGES | CONTROVERSIES

Symptomatic pneumothorax – pigtail/chest tube placement

Hemothorax – hold offending agents, correct coagulopathy or thrombocytopenia if present, and contact IR vs CT surgery

Splenic/liver laceration – hold offending agents, correct coagulopathy or thrombocytopenia if present and contact IR vs general surgery

Diaphragmatic injury – CT surgery consultation

POST-PROCEDURE | INTRA-OP AND POST-OP CHALLENGES | CONTROVERSIES

Bleeding Risk

- Mixed studies regarding thoracentesis bleeding risk on anticoagulation, on antiplatelets or with coagulopathy

Drainage Method

- Most proceed with volume removal with hand pump with some studies showing this having the lowest risk of pneumothorax and REPE
- Most do not pursue gravity given most time consuming method
- Typical hesitancy with vacutainers vs wall suction as potential increase risk of pneumothorax or REPE

Post Procedure Pneumothorax Prevention

- Pleural manometry can help measure pleural elastance and it is recommended if utilized to stop draining fluid if pleural pressure drops below -20 cm H20

- Mixed studies showing potential increased risk of pneumothorax or REPE if > 1500 ml removed

Recap

- [ ] Know Indications and Contraindications
- [ ] Understand risks to procedure and obtaining informed consent
- [ ] Understand equipment, anatomy, and US and optimize positioning to increase success
- [ ] Understand post procedure evaluation and troubleshooting complications

CONTINUE TO QUIZ

Test Your Knowledge

Please answer the 9 multiple choice questions that follow. You must submit each answer before moving on to the next question. A passing score of 80% or better is required to complete the course.

Question

01/09

A 68 yo with ESRD, dCHF, and hypertension presents with subacute progressive dyspnea on exertion and now at rest who is found to have a pleural effusion. 1.4 L is removed without difficulty. After the procedure, the patient notes increased work of breathing and is hypoxic to 80%. HR remains stable in 90s and BP in 120s/80s. What are potential etiologies of this patient's presentation?

- A) Pneumothorax
- B) Hemothorax
- C) Re-expansion pulmonary edema
- A and B
- A, B, and C

Question

02/09

When removing fluid during thoracentesis, what is an indicator that you should terminate the procedure?

- ○ cough
- ○ manometry of + 10 cm H20
- ○ 1.2 L maximum fluid removal
- ○ significant chest pain

Question

03/09

Ultrasound evaluation of a pleural effusion can help assess for all but _____.

- ○ size of effusion
- ○ complexity of effusion
- ○ depth of subcutaneous tissues
- ○ vessels along expected needle approach if fluid is an empyema

Question

04/09

A 45 yo man with hx of class III obesity, CAD s/p PCI five years ago, afib on Xarelto, HTN, COPD, and tobacco abuse presents to clinic for evaluation of a small pleural effusion incidentally found on abd/pelvic CT when he was hospitalized for abdominal pain. The patient is currently taking 25 mg metoprolol succinate, 81 mg aspirin, and 5 mg Eliquis bid. The patient's abdominal pain improved during a short hospitalization and outpatient referral was made to pulmonology to evaluate his small right sided effusion. You desire to perform diagnostic thoracentesis and should recommend the patient:

- To proceed with the thoracentesis.
- Hold aspirin and Xarelto and proceed with thoracentesis in 8 days.
- Hold Xarelto and proceed with the thoracentesis in 24 to 48 hours.
- None of the above.

Question

05/09

Which is a strict contraindication to a therapeutic thoracentesis?

- Overlying skin infection
- Mechanically ventilated patient
- INR > 2
- Suspected lung entrapment
- None of the above

Question

06/09

What is the minimum distance between the parietal pleura and adjacent structures in all 3 dimensions that is safe for thoracentesis?

- ○ 1 cm
- ○ 2 cm
- ○ 3 cm
- ○ 5 cm

Question

07/09

Which of the following techniques is most likely to reduce the risk of re-expansion pulmonary edema while performing a thoracentesis?

- ○ Using vacutainer for fluid removal
- ○ Discontinue procedure if patient coughs
- ○ Discontinue procedure if increased resistance is felt when removing fluid
- ○ Discontinue procedure once 1.2 L is removed

Question

08/09

Which technique is most likely to reduce the risk of causing intercostal bleeding while performing a thoracentesis?

- ○ selecting a posterior insertion site with accessible fluid within 6 cm of midline of spine
- ○ use of color power doppler and color doppler to identity costal vessels at insertion site
- ○ fist contacting the rib and then advancing catheter cranially into pleural effusion
- ○ assuring PLT level is > 100 x109/L and INR < 1.5

Question

09/09

A 39 yo with severe COPD on 2 L NC, HTN, hyperlipidemia presents with acute on chronic hypoxic respiratory failure found to have a new small to moderate right sided pleural effusion.

VS: 37.4, 90, 128/90, 98% on 5 L NC, BMI of 17.

There are no contraindications to thoracentesis, therefore the patient is consented, and you aim to proceed with the procedure.

What places this patient at increased risk for pneumothorax?

- ○ Moderate to large sized pleural effusion
- ○ Right sided effusion
- ○ BMI < 18
- ○ Anticipated removal of > 1.5 L
- ○ None of the above
