# Supplementary material for: Introducing a Comprehensive Framework for Competency-based Procedure Training
Source: J Gen Intern Med. 2025 Jul 8;40(15):3560–5. doi: 10.1007/s11606-025-09677-2 (PMC12612326; doi:10.1007/s11606-025-09677-2)

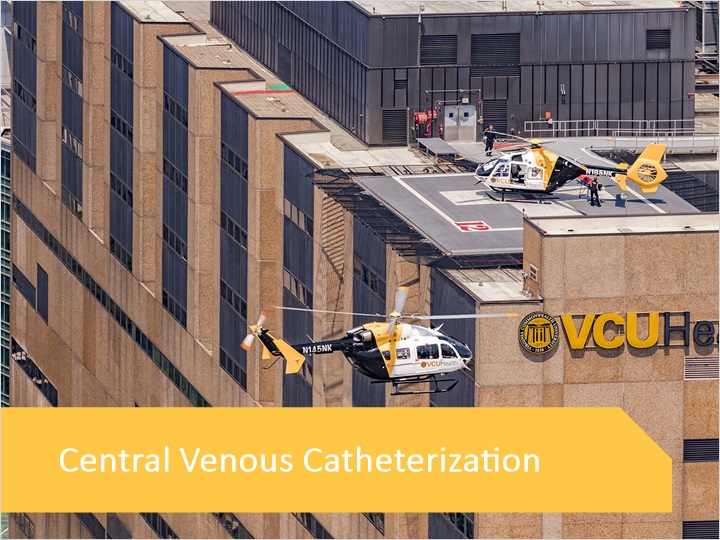


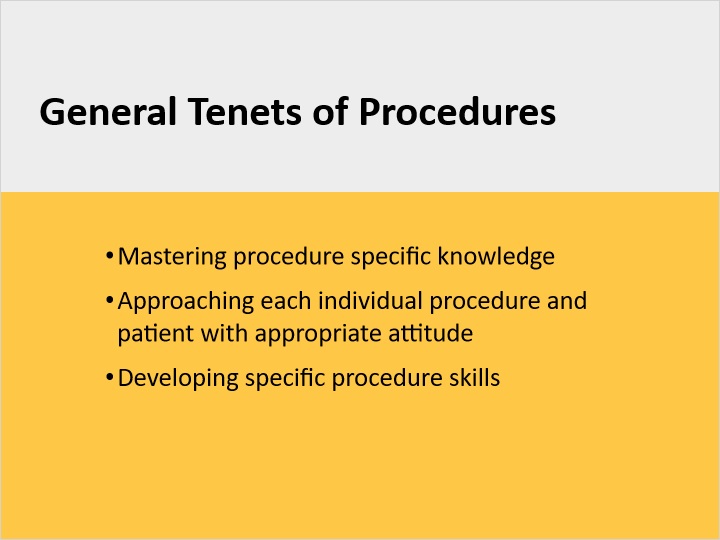


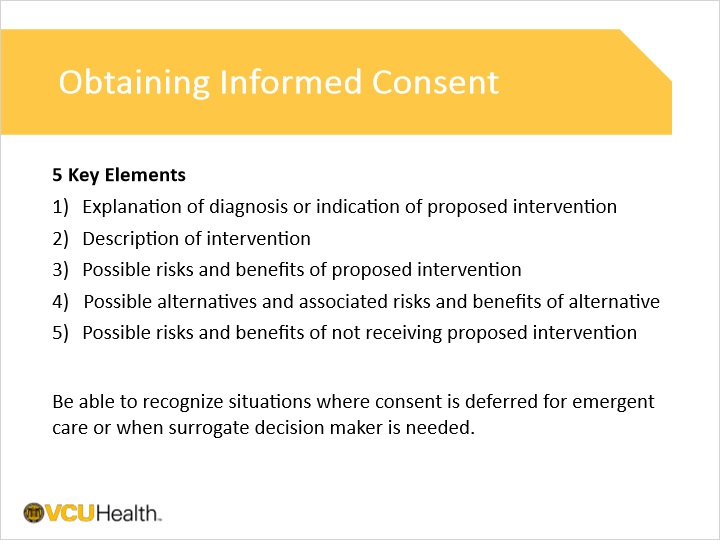


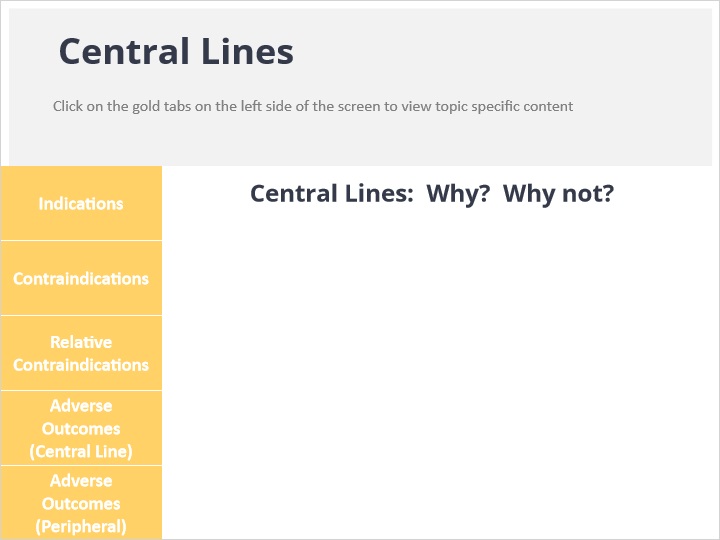


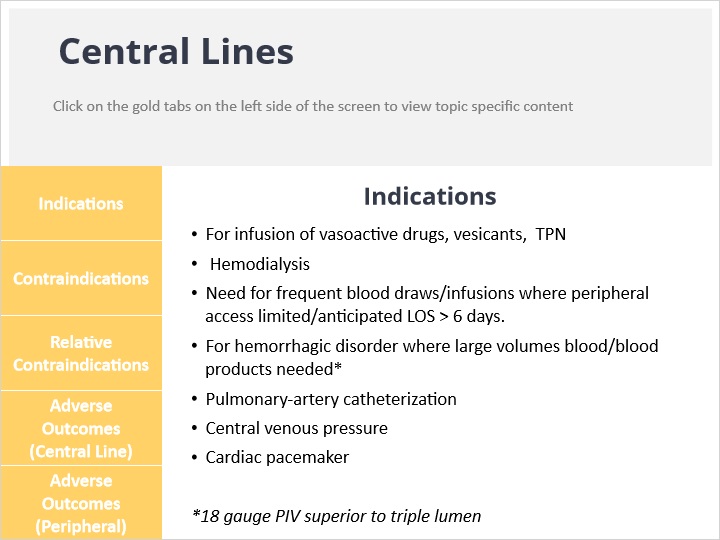


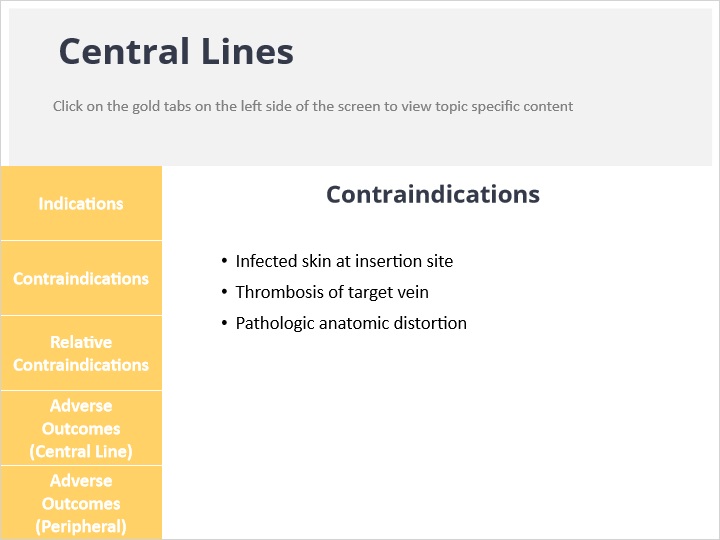


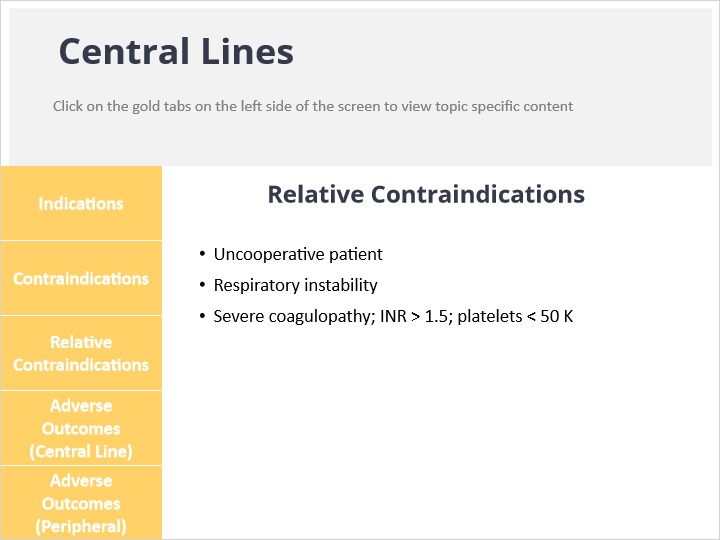


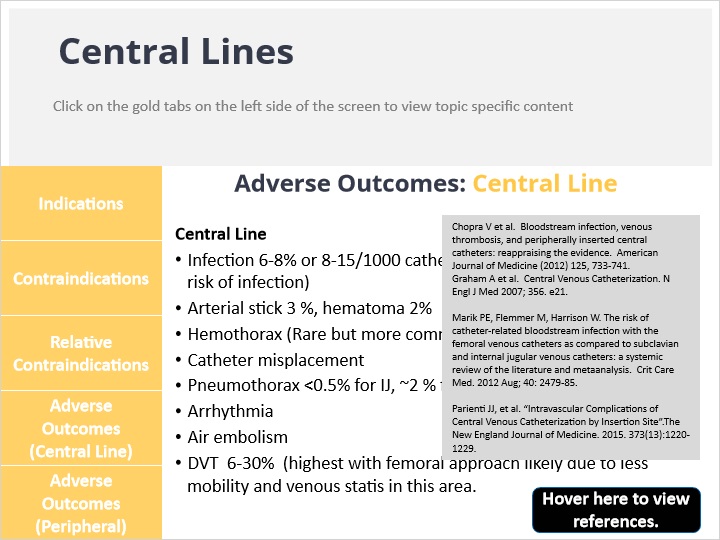


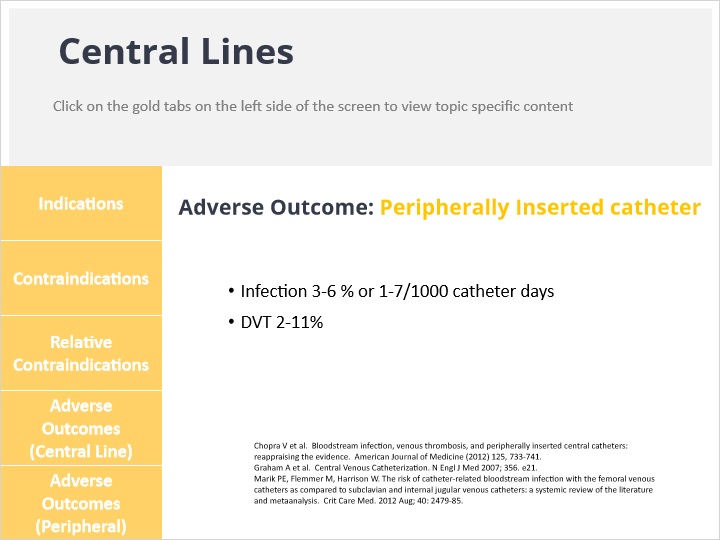


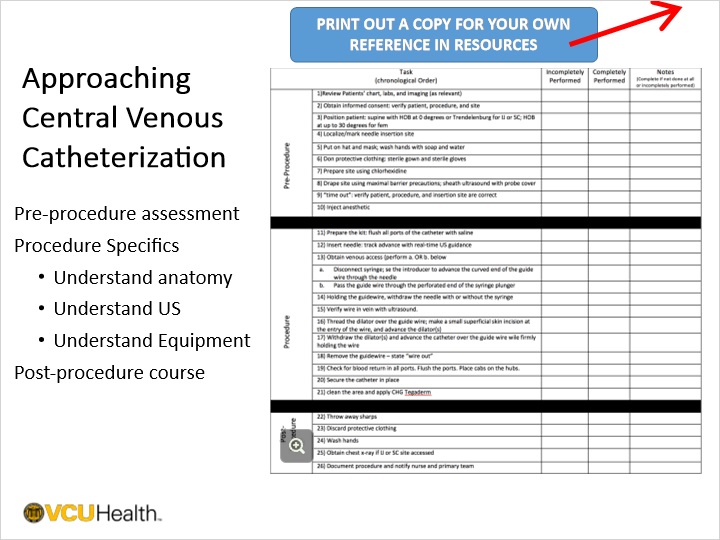


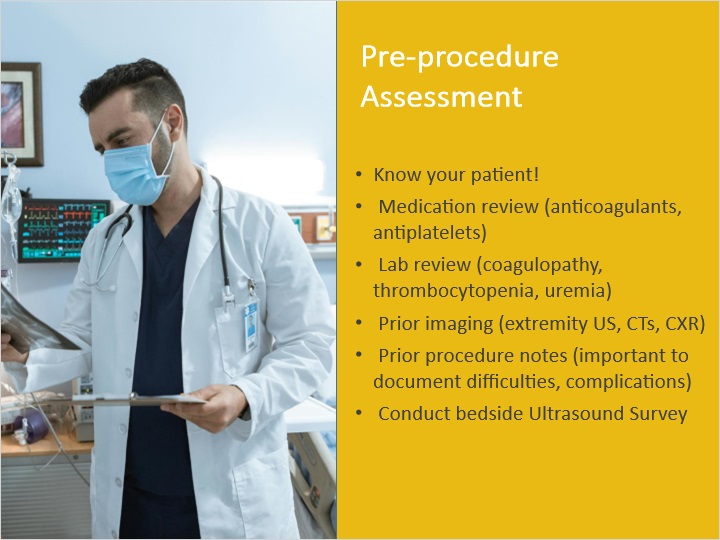


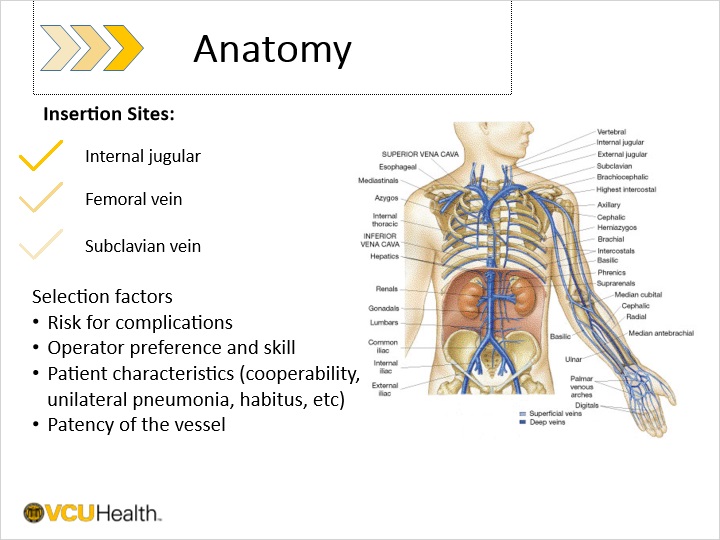


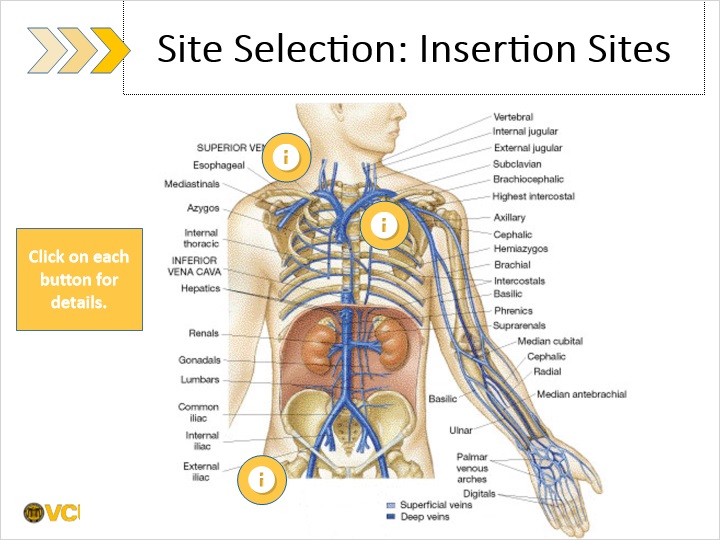


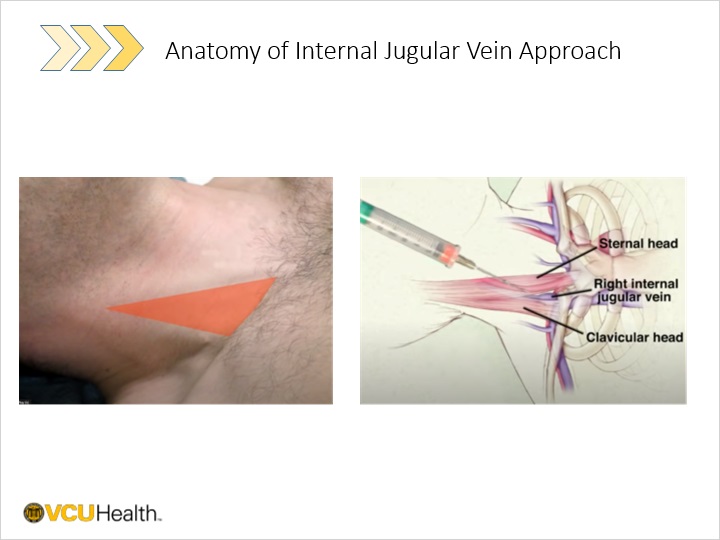


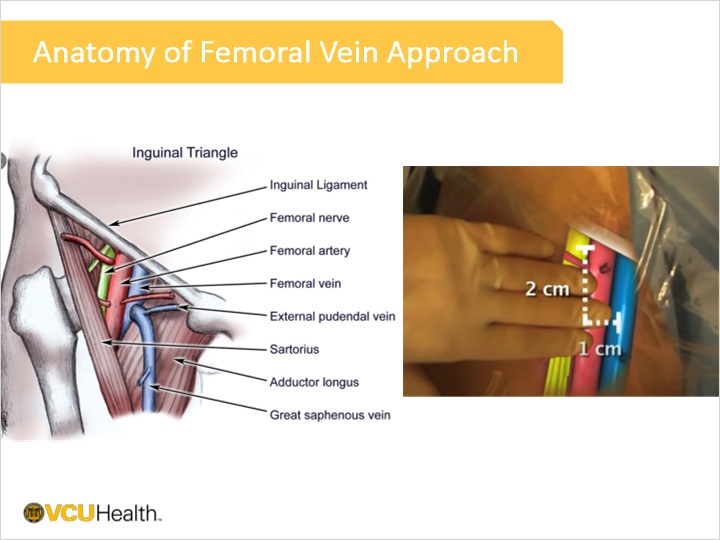


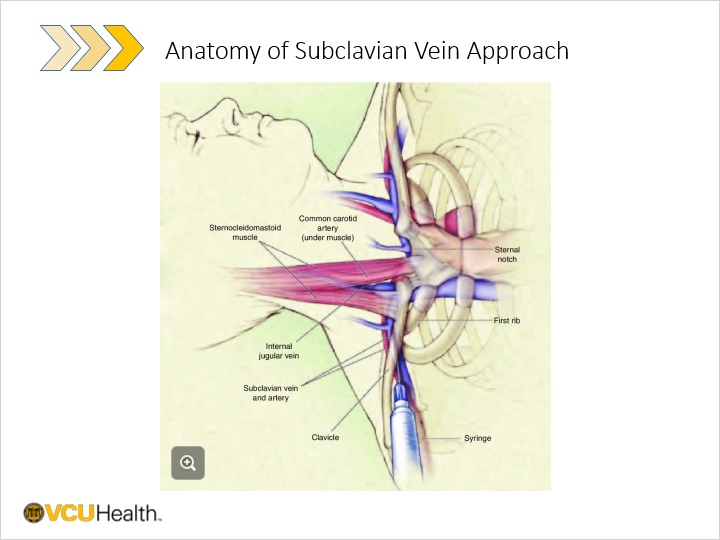


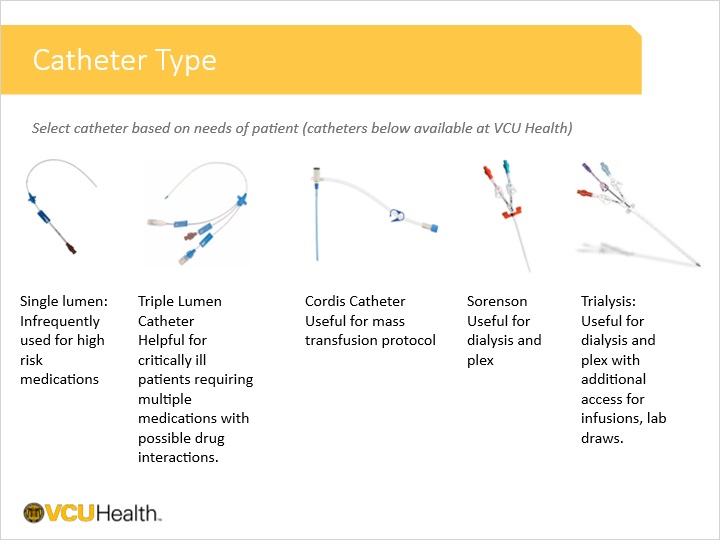


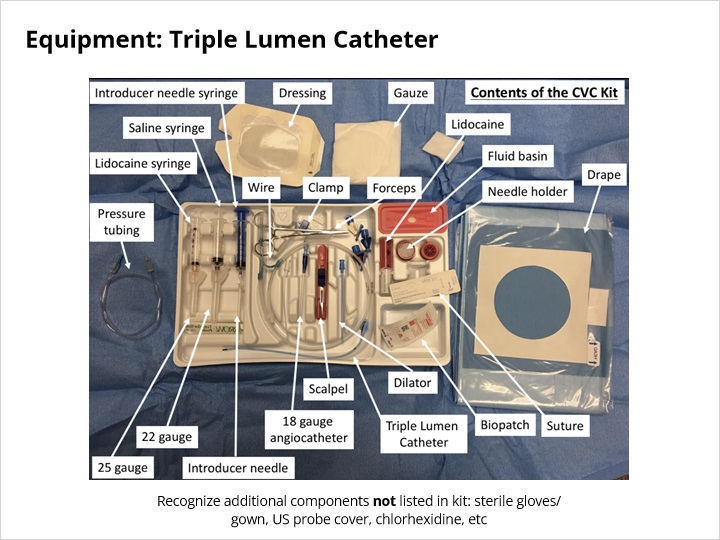


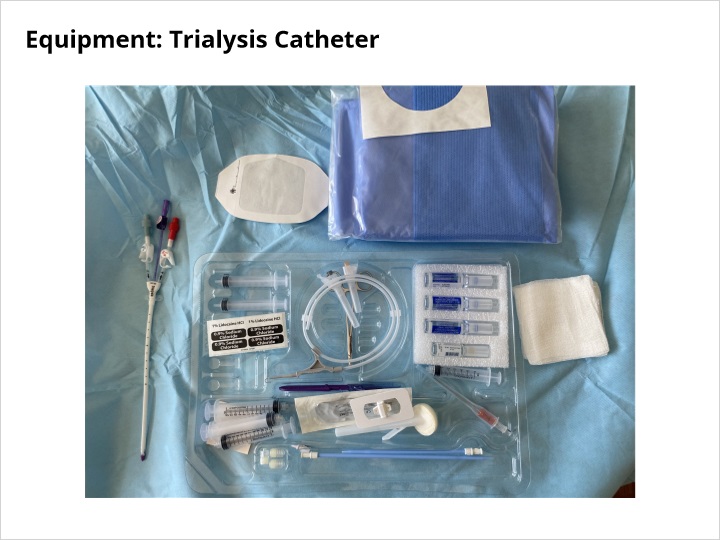


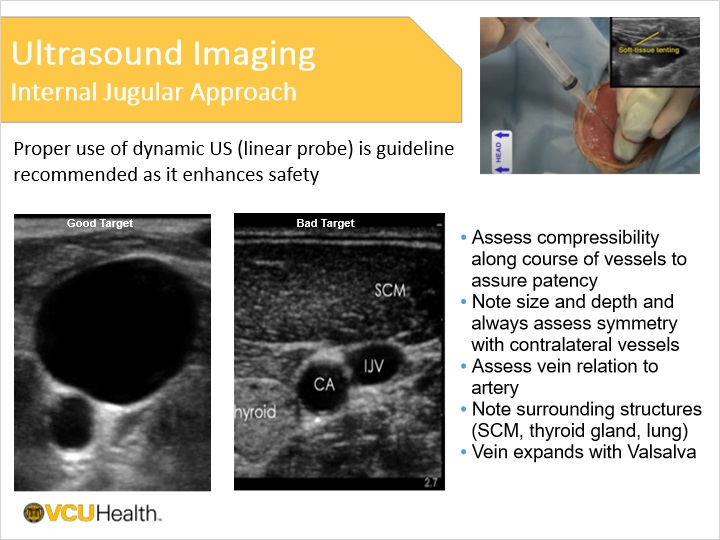


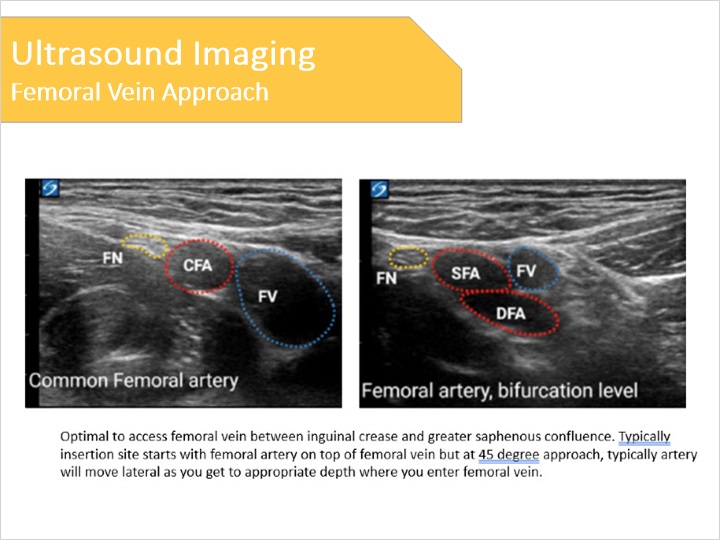


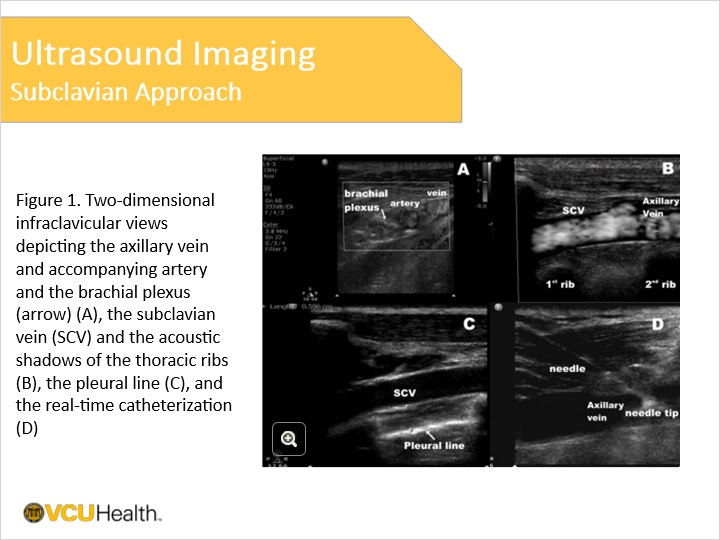


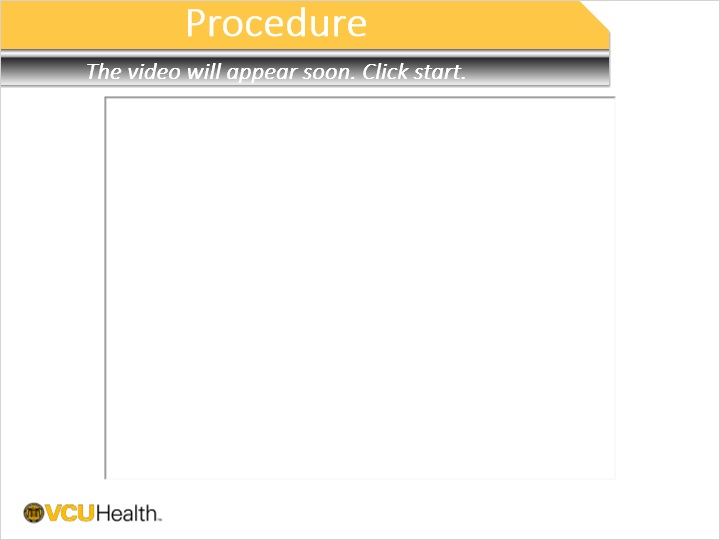


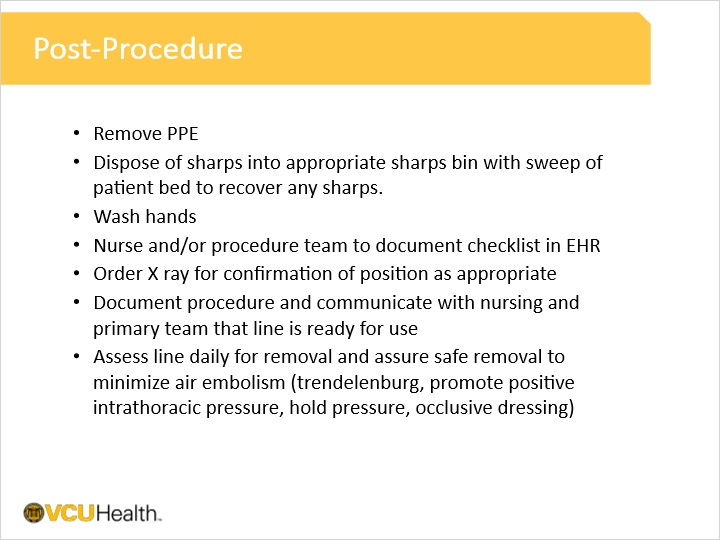


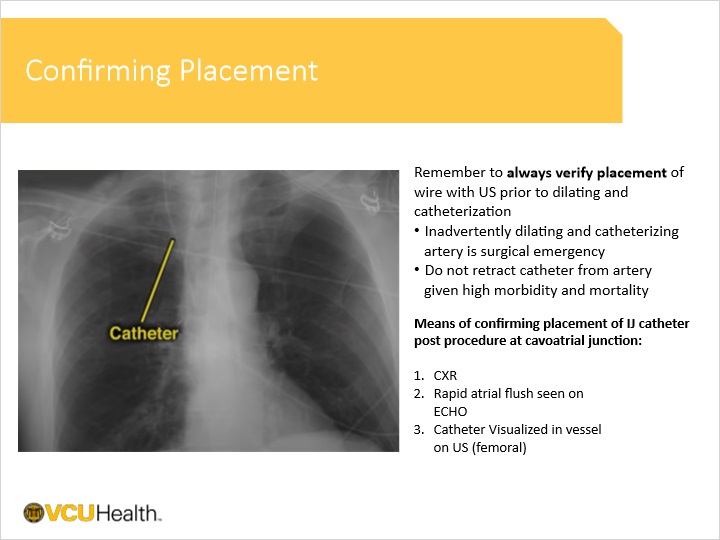


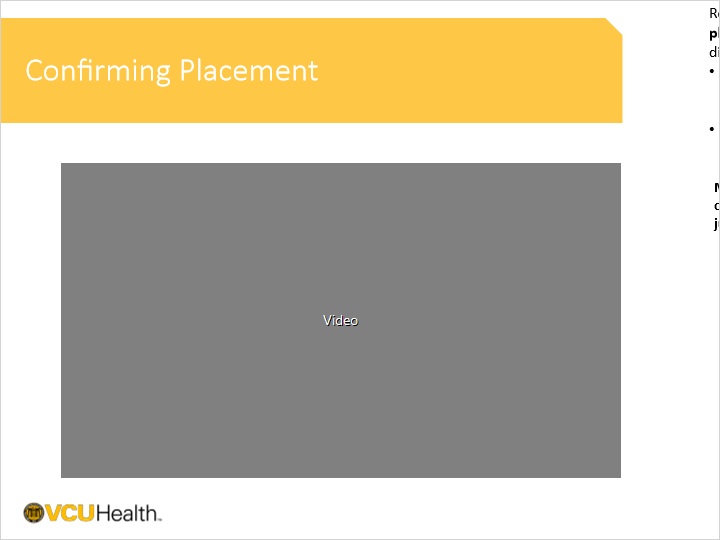


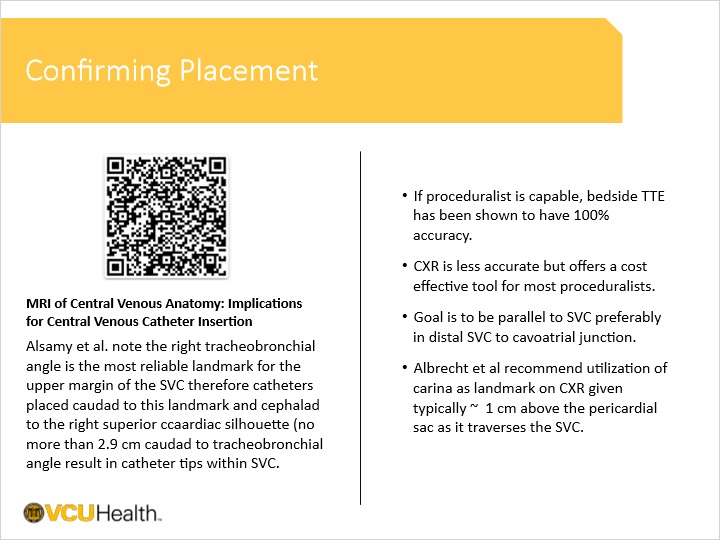


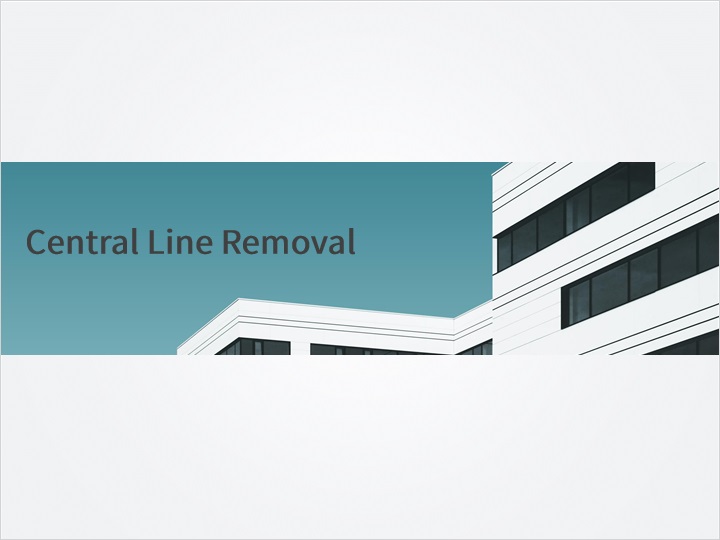


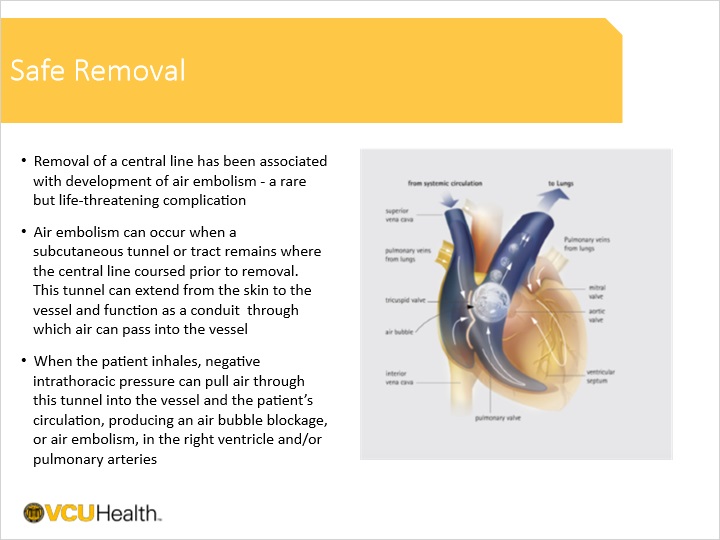


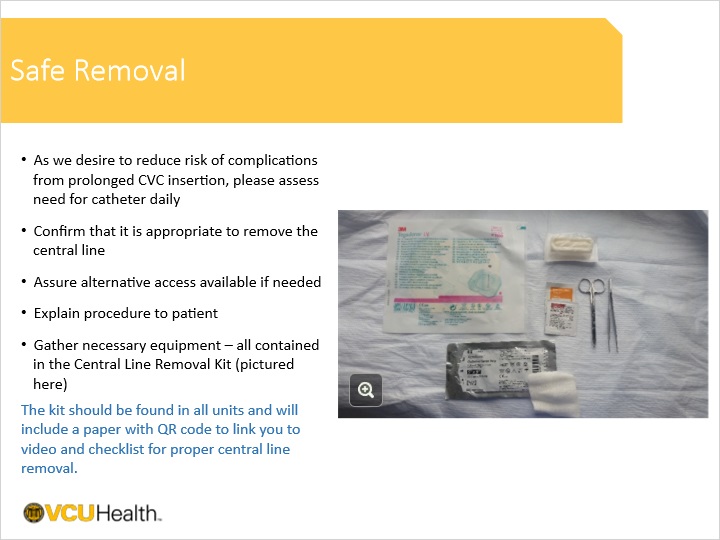


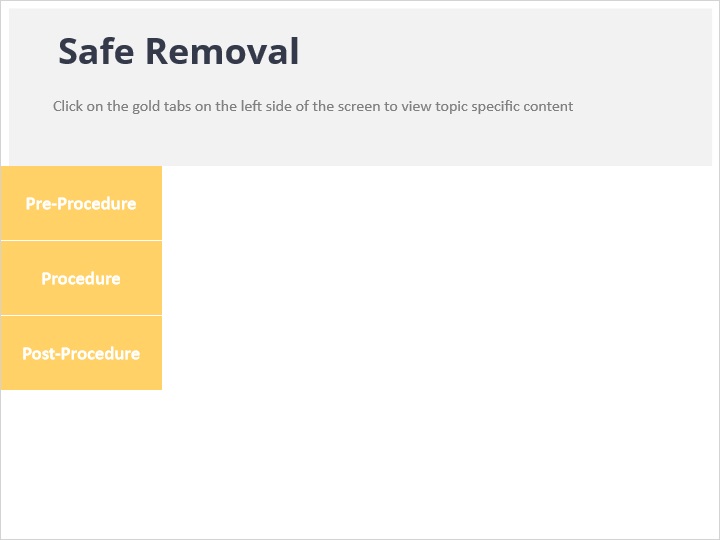


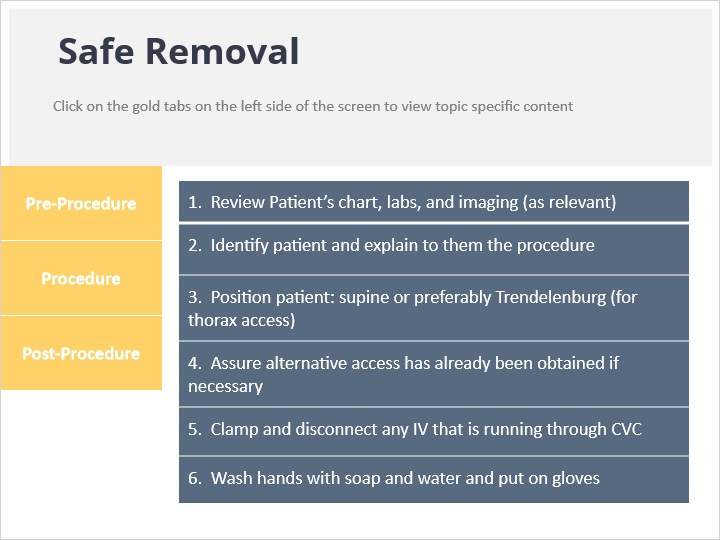


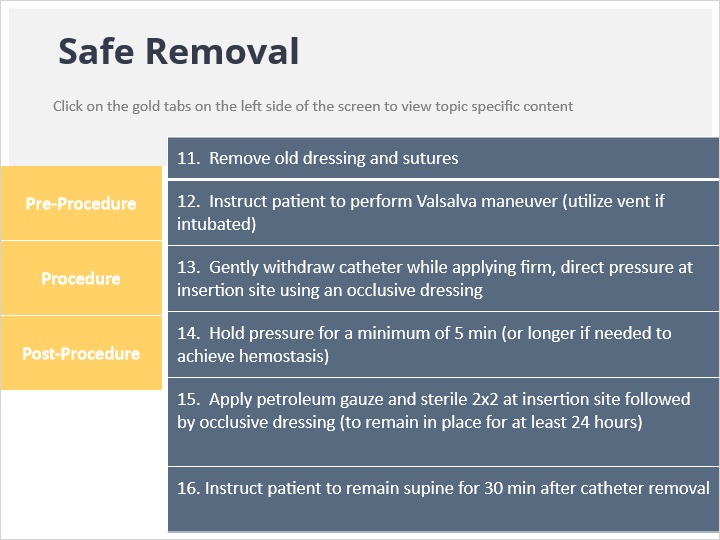


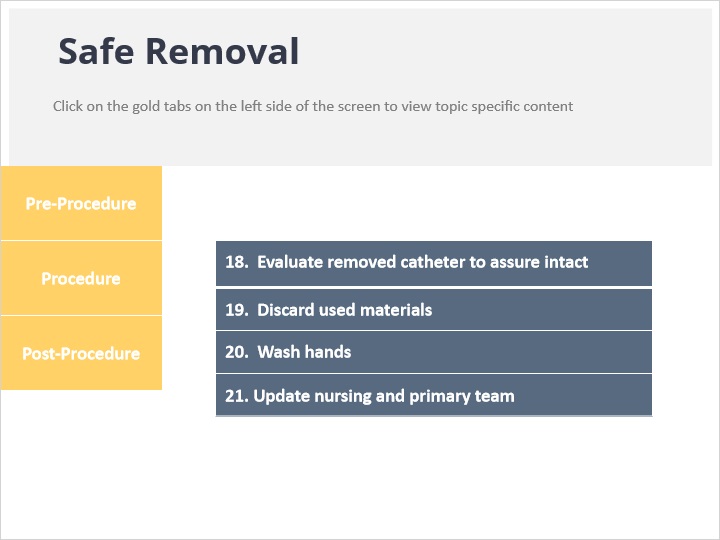


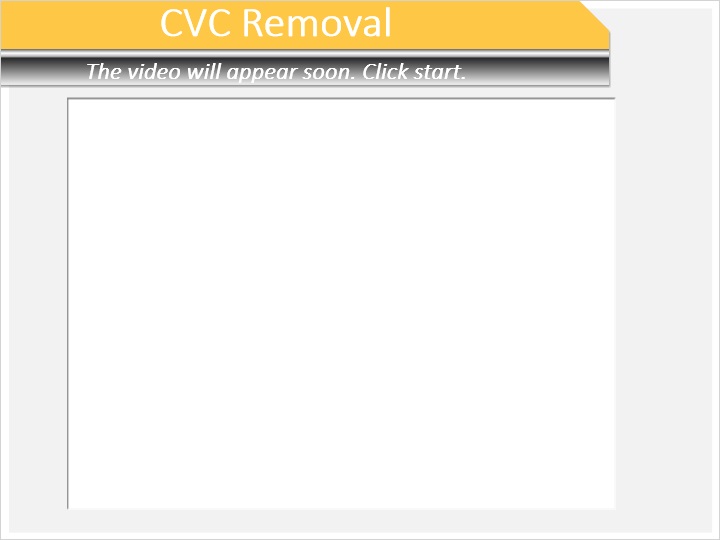


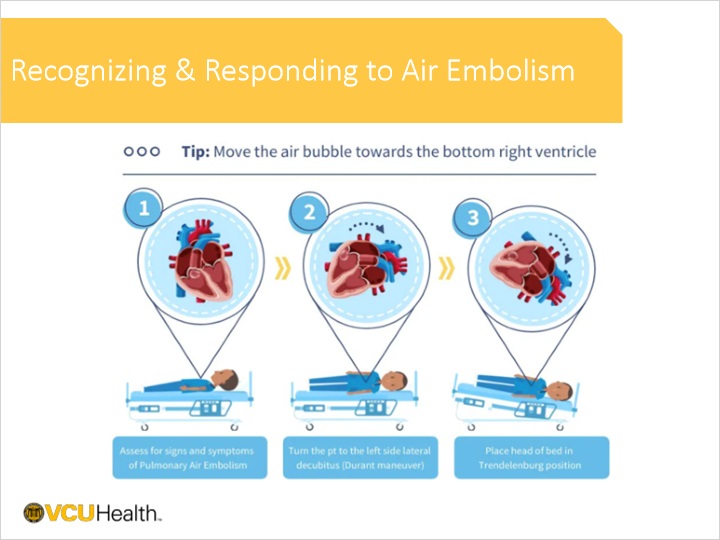


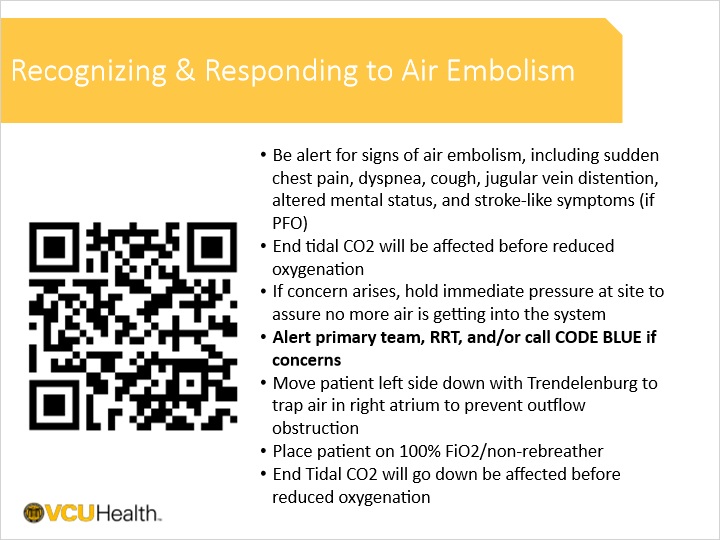


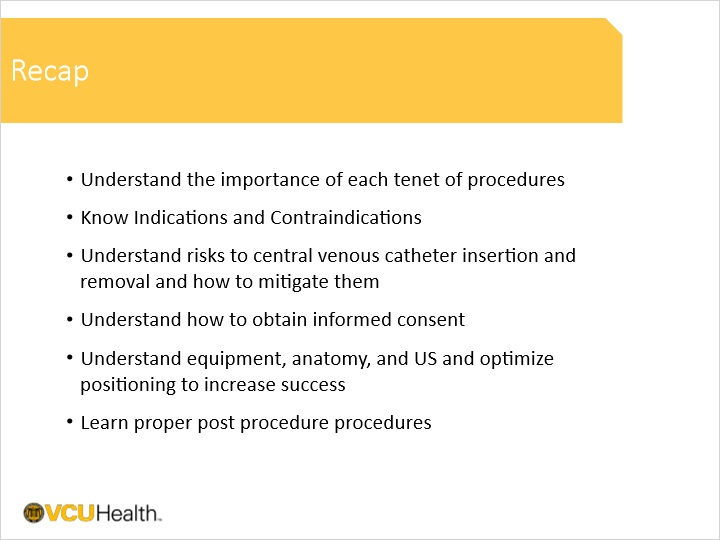


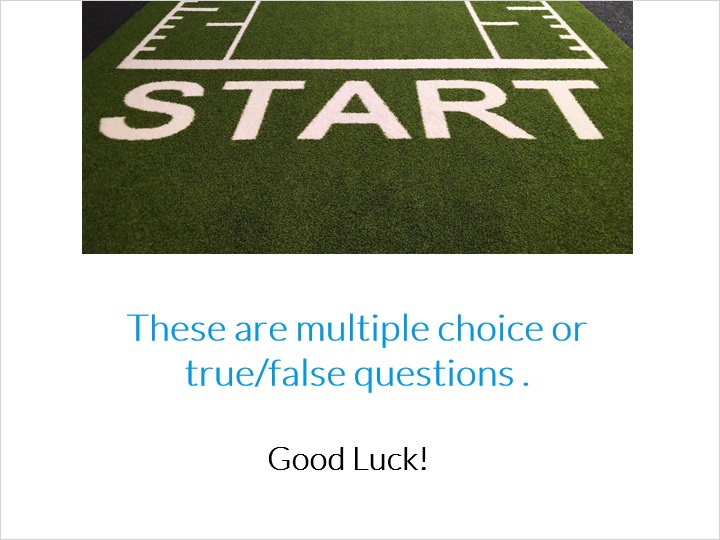


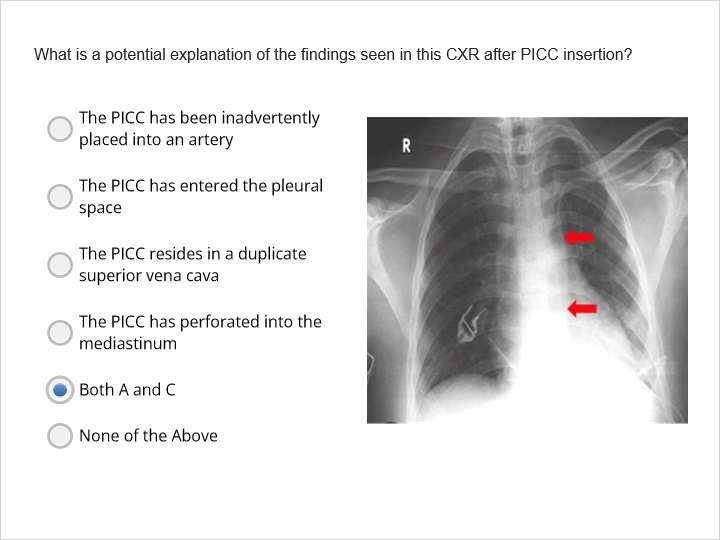


| Correct | Choice |
| --- | --- |
|  | The PICC has been inadvertently placed into an artery |
|  | The PICC has entered the pleural space |
|  | The PICC resides in a duplicate superior vena cava |
|  | The PICC has perforated into the mediastinum |
| X | Both A and C |
|  | None of the Above |

**Feedback when correct:**

That's right! You selected the correct response. This placement can certainly be consistent with arterial cannulation, however was actually placed in a duplicate SVC. Only 0.3% of the population has this anatomical variation. In patients with a duplicate superior vena cava, 82% of patients have normal right-sided SVC with persistent left SVC. In 90% of cases, the left supervisor vena cava drains into the coronary sinus followed by the right atrium, and thus, the patient is typically asymptomatic and does not require any treatment. In these cases, placement of a central venous catheter can cause irritation of the coronary sinus and result in hypotension, arrythmia, myocardial ischemia, and cardiac arrest. In the remaining 8 % to 10 % of cases, a persistent left SVC may drain into the left atrium, forming a right-to-left shunt which can give risk to systemic air or particulate emboli from catheter use and places the patient at an increased risk of developing right-sided heart failure. Therefore, it is imperative to know the drainage of the duplicate supervisor vena cava prior to use, and likely best to not leave this line in its current position.

**Feedback when incorrect:**

You did not select the correct response.

### Try Again (Slide Layer)


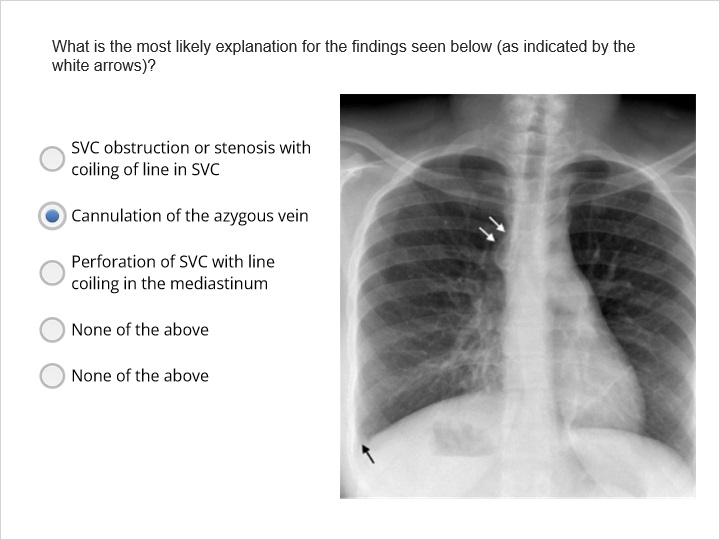


| Correct | Choice |
| --- | --- |
|  | SVC obstruction or stenosis with coiling of line in SVC |
| X | Cannulation of the azygous vein |
|  | Perforation of SVC with line coiling in the mediastinum |
|  | None of the above |
|  | None of the above |

**Feedback when correct:**

That's right! You selected the correct response. Azygous cannulation can occur given its location especially with cannulation from left internal jugular or subclavian. This line should be exchanged with assured proper placement to avoid mediastinal perforation or thrombosis.

**Feedback when incorrect:**

You did not select the correct response.


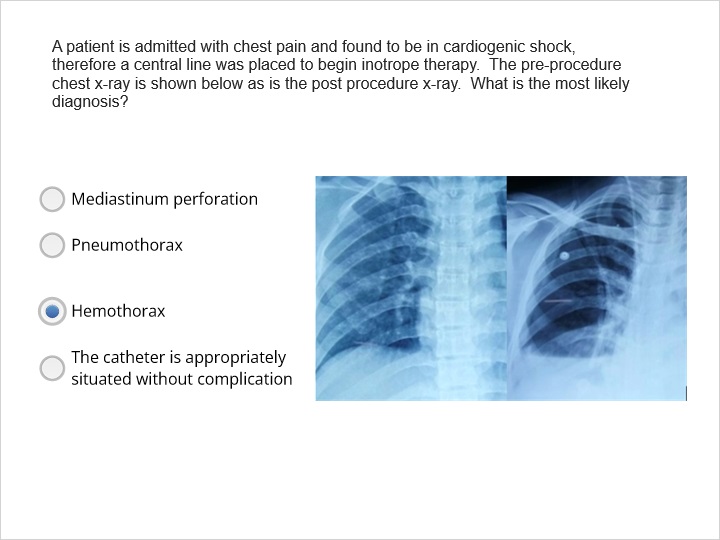


| Correct | Choice |
| --- | --- |
|  | Mediastinum perforation |
|  | Pneumothorax |
| X | Hemothorax |
|  | The catheter is appropriately situated without complication |

**Feedback when correct:**

That's right! You selected the correct response. There is a subtle development of a right sided effusion. Be careful not to only evaluate for proper line placement but also to assure no new pulmonary or mediastinal pathology. In this case, there is a new temporally related effusion which is concerning for hemothorax.

**Feedback when incorrect:**

You did not select the correct response.


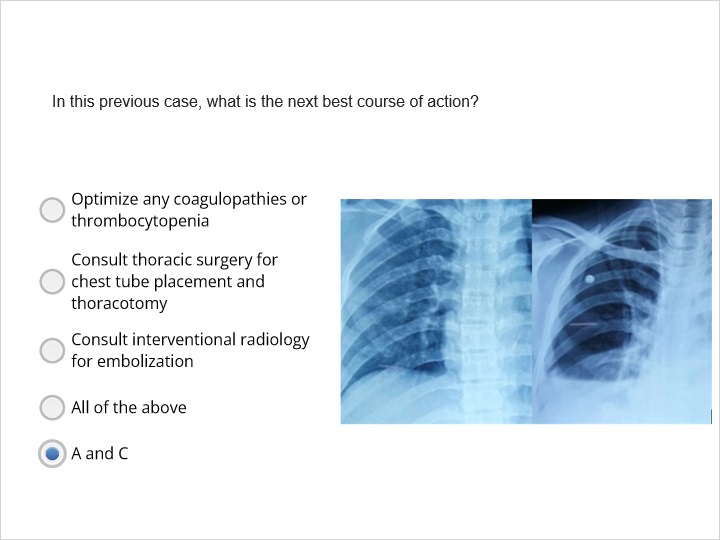


| Correct | Choice |
| --- | --- |
|  | Optimize any coagulopathies or thrombocytopenia |
|  | Consult thoracic surgery for chest tube placement and thoracotomy |
|  | Consult interventional radiology for embolization |
|  | All of the above |
| X | A and C |

**Feedback when correct:**

That's right! You selected the correct response. In this case, you may consider each of the above simultaneously based on patient specific factors. Correcting factors can affect bleeding will be important but stabilizing patient with chest tube and discussing with surgical approach versus intravascular approach will be vital.

**Feedback when incorrect:**

You did not select the correct response.


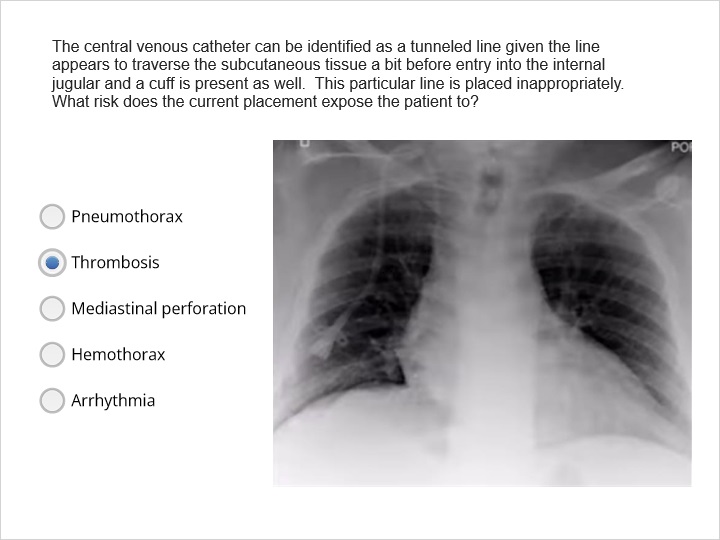


| Correct | Choice |
| --- | --- |
|  | Pneumothorax |
| X | Thrombosis |
|  | Mediastinal perforation |
|  | Hemothorax |
|  | Arrhythmia |

**Feedback when correct:**

That's right! You selected the correct response. Central venous catheters placed in the superior vena cava have been found to have a higher risk of thrombosis.

**Feedback when incorrect:**

You did not select the correct response.


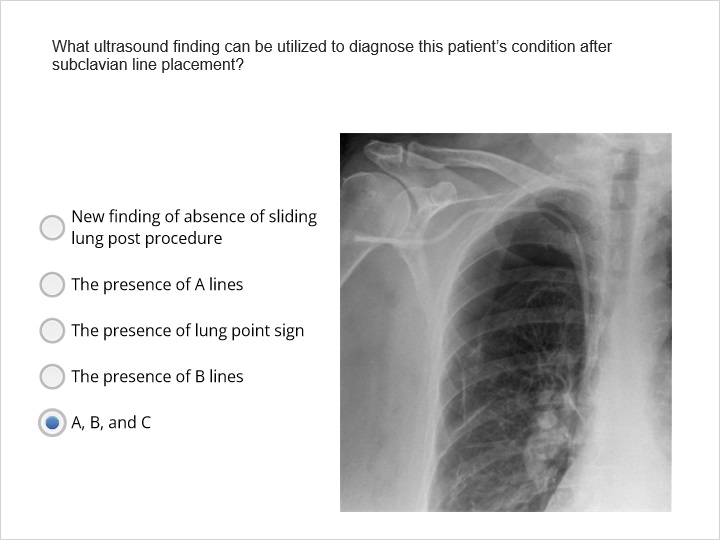


| Correct | Choice |
| --- | --- |
|  | New finding of absence of sliding lung post procedure |
|  | The presence of A lines |
|  | The presence of lung point sign |
|  | The presence of B lines |
| X | A, B, and C |

**Feedback when correct:**

That's right! You selected the correct response. Absence of sliding lung post procedure when previously visible is highly specific and sensitive for diagnosing a pneumothorax with higher sensitivity and specificity than CXR. If one has not evaluated pre-procedure and no lung sliding is evident post procedure, one must recognize that other physiology may be contributing to this finding: ARDS, pulmonary fibrosis, large consolidations, pleural adhesions, atelectasis, right mainstem intubation, phrenic nerve paralysis. If A lines are present and there is no evidence of lung sliding, then there is a higher sensitivity and specific for pneumothorax. B lines tend to be absent if pneumothorax in pneumothorax so if visible, they essentially rule out pneumothorax given their high negative predictive value. Lung point sign shows an edge of the pneumothorax and has a higher sensitivity compared to absence of lung sliding alone.

**Feedback when incorrect:**

You did not select the correct response.


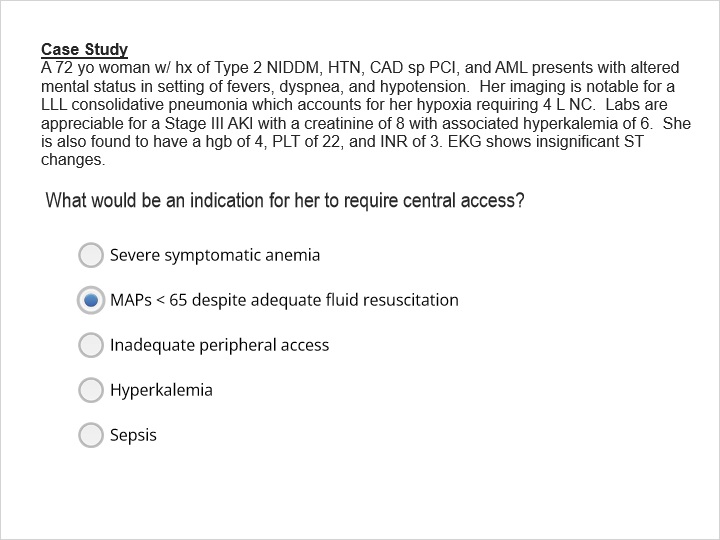


| Correct | Choice |
| --- | --- |
|  | Severe symptomatic anemia |
| X | MAPs < 65 despite adequate fluid resuscitation |
|  | Inadequate peripheral access |
|  | Hyperkalemia |
|  | Sepsis |

**Feedback when correct:**

That's right! You selected the correct response. MAPS < 65 despite appropriate fluid rescuscitation is the most appropriate response. There is literature to support low dose pressors through peripheral access but in most cases this patient will be receiving a central venous catheter. If patient was having EKG changes with medically refractory hyperkalemia this would warrant HD line placement. Inadequate peripheral access may lead to the need for a central line, but US should be utilized to assess for peripheral access beforehand if patient has no other indication for central access with a promising clinical trajectory.

**Feedback when incorrect:**

You did not select the correct response.


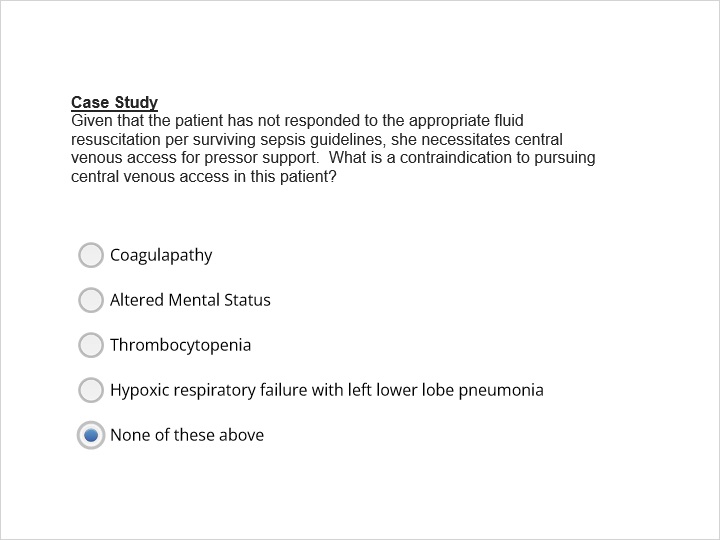


| Correct | Choice |
| --- | --- |
|  | Coagulapathy |
|  | Altered Mental Status |
|  | Thrombocytopenia |
|  | Hypoxic respiratory failure with left lower lobe pneumonia |
| X | None of these above |

**Feedback when correct:**

That's right! You selected the correct response. Coagulopathies and thrombocytopenia are relative contraindications and as this line is becoming more urgent would not preclude or preclude or delay central line from being placed. One should always be cautious in a patient with altered mental status for patient and operator safety. One might need to cautiously consider some sedation to assure proper and safe placement. Hypoxic respiratory failure is a condition to consider when considering location of access. One might avoid a subclavian approach in a patient on maximum vent settings or attempt to place line on most affected lung (IJ or subclavian) if no other access opportunities.

**Feedback when incorrect:**

You did not select the correct response.

## 1.40 Case Study


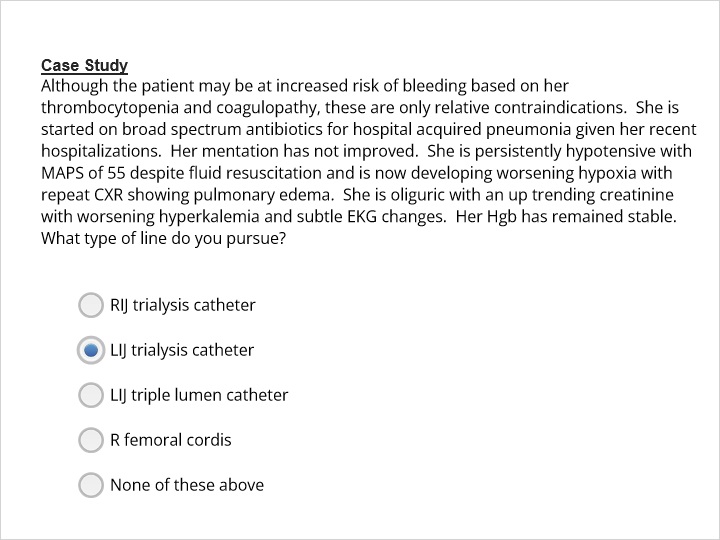


| Correct | Choice |
| --- | --- |
|  | RIJ trialysis catheter |
| X | LIJ trialysis catheter |
|  | LIJ triple lumen catheter |
|  | R femoral cordis |
|  | None of these above |

**Feedback when correct:**

That's right! You selected the correct response. A trialysis catheter is desired given need for continuous replacement therapy and the left side is preferred given patient has a known large LLL consolidation. It is ideal to avoid potential pneumothorax on a patient with increasing oxygen requirements on the better functioning lung. There are some that feel L sided approach may affect hemodialysis so this can be discussed further with your local nephrologist.

**Feedback when incorrect:**

You did not select the correct response.


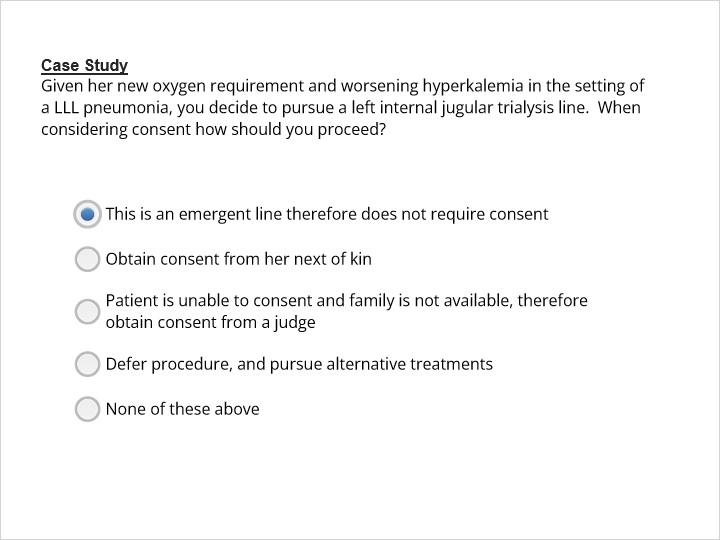


| Correct | Choice |
| --- | --- |
| X | This is an emergent line therefore does not require consent |
|  | Obtain consent from her next of kin |
|  | Patient is unable to consent and family is not available, therefore obtain consent from a judge |
|  | Defer procedure, and pursue alternative treatments |
|  | None of these above |

**Feedback when correct:**

That's right! You selected the correct response. The patient is hypotensive and with worsening respiratory status in need of dialysis therefore classifies as being acceptable to place emergently especially if unable to gain consent from next of kin.

**Feedback when incorrect:**

You did not select the correct response.


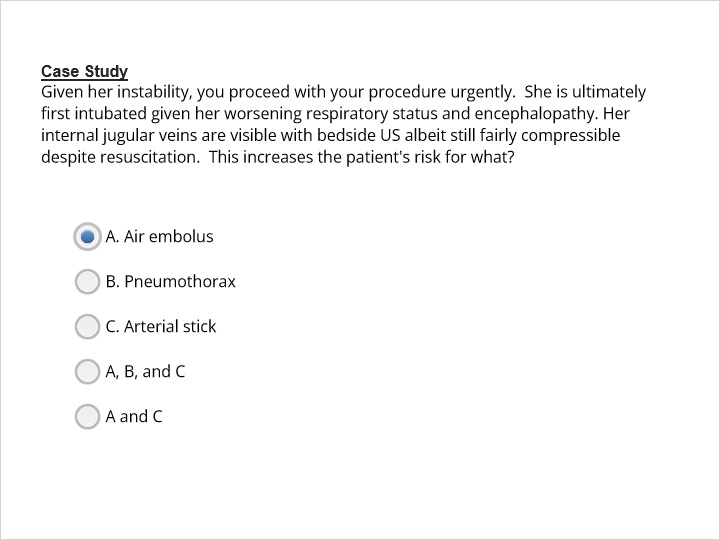


| Correct | Choice |
| --- | --- |
| X | A. Air embolus |
|  | B. Pneumothorax |
|  | C. Arterial stick |
|  | A, B, and C |
|  | A and C |

**Feedback when correct:**

That's right! You selected the correct response. There is an increased risk of air embolus in this case given patient is hypovolemic and therefore max Trendelenburg and Valsalva maneuvers should be utilized. There should not be an increased risk of arterial stick unless the vein is positioned on top of the artery which should ideally be avoided if the internal jugular is completely collapsible. There should be no increased risk of pneumothorax if one is tracking needle appropriately with US.

**Feedback when incorrect:**

You did not select the correct response.


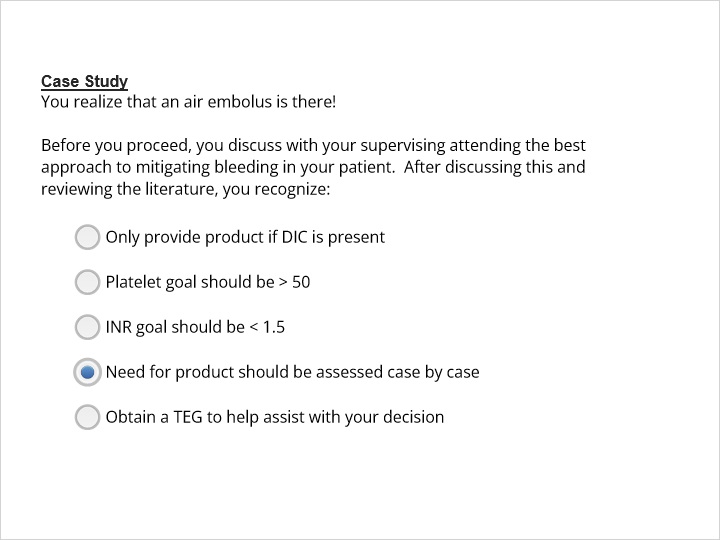


| Correct | Choice |
| --- | --- |
|  | Only provide product if DIC is present |
|  | Platelet goal should be > 50 |
|  | INR goal should be < 1.5 |
| X | Need for product should be assessed case by case |
|  | Obtain a TEG to help assist with your decision |

**Feedback when correct:**

That's right! You selected the correct response. In emergent cases there may not be an opportunity to mitigate bleeding risk especially if there are access issues. There is literature to suggest higher bleeding risk with PLT < 40, but it is not an absolute contraindication to pursuing the procedure. The literature has not supported the regular correction of coagulopathies. Ultimately, it is best to evaluate each case based on their anatomy and associated parameters.

**Feedback when incorrect:**

You did not select the correct response.


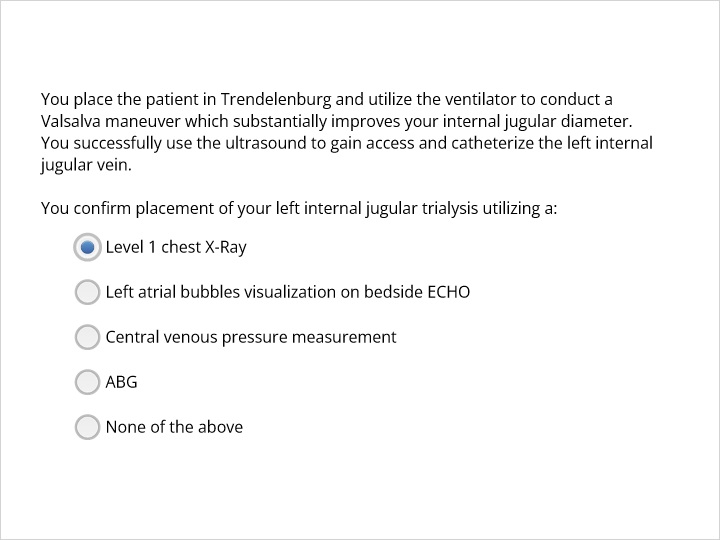


| Correct | Choice |
| --- | --- |
| X | Level 1 chest X-Ray |
|  | Left atrial bubbles visualization on bedside ECHO |
|  | Central venous pressure measurement |
|  | ABG |
|  | None of the above |

**Feedback when correct:**

That's right! You selected the correct response. CXR is an acceptable approach to verifying line placement and can also assist in assessing for line associated pneumothorax. TTE may assist with visualization of line and right atrial bubbles also can be utilized to assess placement. ABG can be of use if there is concern for arterial cannulation.

**Feedback when incorrect:**

You did not select the correct response.


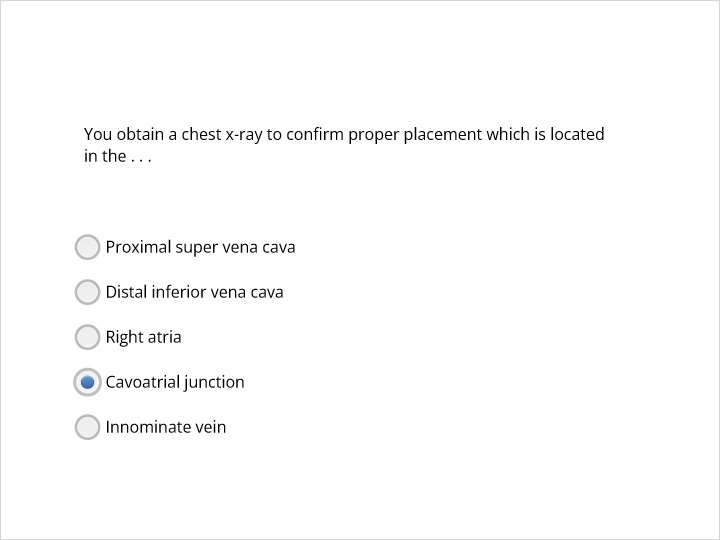


| Correct | Choice |
| --- | --- |
|  | Proximal super vena cava |
|  | Distal inferior vena cava |
|  | Right atria |
| X | Cavoatrial junction |
|  | Innominate vein |

**Feedback when correct:**

That's right! You selected the correct response. Proper placement is at the cavo-atrial junction.

**Feedback when incorrect:**

You did not select the correct response.


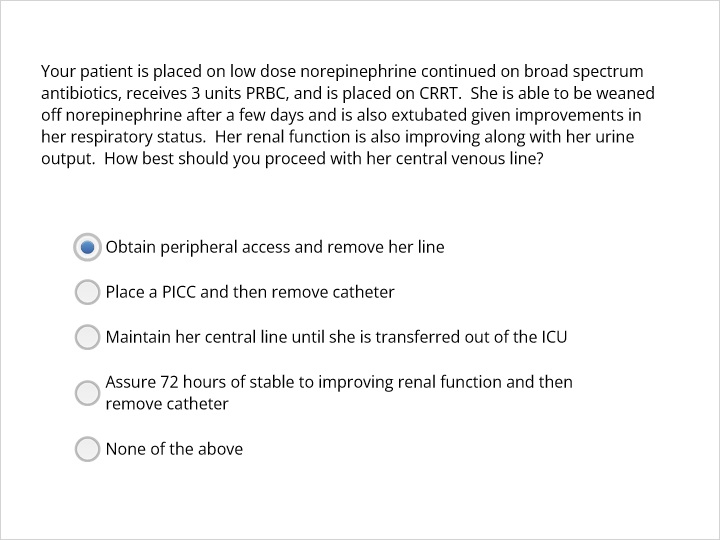


| Correct | Choice |
| --- | --- |
| X | Obtain peripheral access and remove her line |
|  | Place a PICC and then remove catheter |
|  | Maintain her central line until she is transferred out of the ICU |
|  | Assure 72 hours of stable to improving renal function and then remove catheter |
|  | None of the above |

**Feedback when correct:**

That's right! You selected the correct response. This patient has been weaned off norepinephrine with continued hemodynamic stability and has had marked improvements in renal function and respiratory function to no longer necessitate central access. Peripheral access should be obtained and the central catheter removed to reduce infection and thrombosis risk.

**Feedback when incorrect:**

You did not select the correct response.


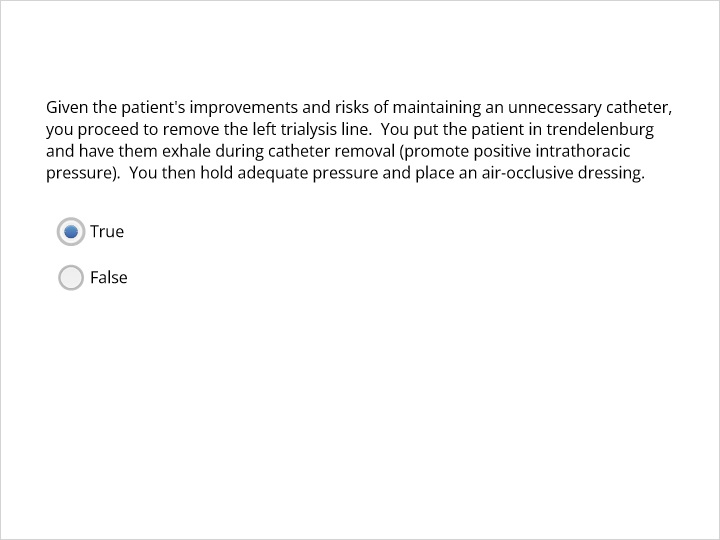


| Correct | Choice |
| --- | --- |
| X | True |
|  | False |

**Feedback when correct:**

That's right! You selected the correct response. This is proper technique for removal of a central line in the thorax to reduce the risk of air embolism.

**Feedback when incorrect:**

You did not select the correct response.

## 1.48 A patient is intubated and sedated in the ICU for hypoxia respiratory failure and septic shock secondary to aspiration pneumonia. The patient has had improved hemodynamics no longer requiring pressor support for the last 48 hours, however has developed recurrent fevers and found to have a GPC bacteremia. Peripheral access is adequate, therefore the right sided internal jugular central line is removed with appropriate technique. The patient becomes hypotensive post line removal and the end tidal CO2 level has increased.

## What is the next best approach in the management of this patient?

*(Multiple Choice, 10 points, 2 attempts permitted)*


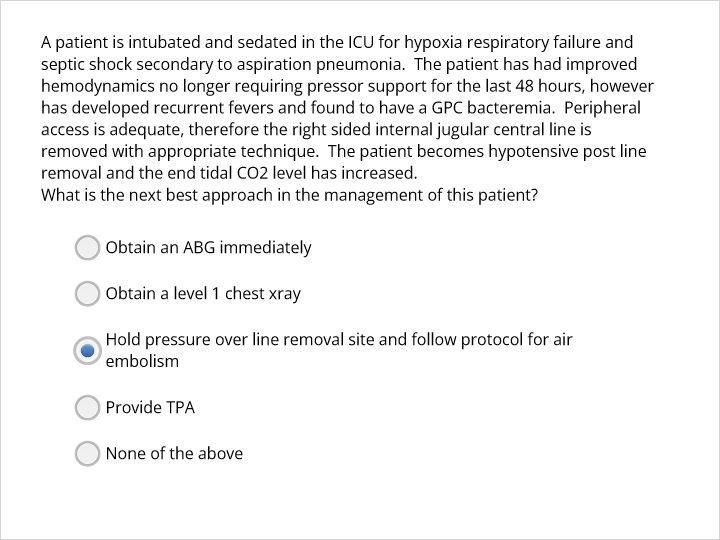


| Correct | Choice |
| --- | --- |
|  | Obtain an ABG immediately |
|  | Obtain a level 1 chest xray |
| X | Hold pressure over line removal site and follow protocol for air embolism |
|  | Provide TPA |
|  | None of the above |

**Feedback when correct:**

That's right! You selected the correct response. Ultimately this patient is having typical findings seen with an air embolism and considering the temporal relationship of line removal, this is the most likely diagnosis. Therefore a code versus rapid response should be called, immediate pressure should be held over insertion site, the patient should be placed in the left lateral decubitus position, placed in Trendelenburg, and FIO2 increased to 100%.

**Feedback when incorrect:**


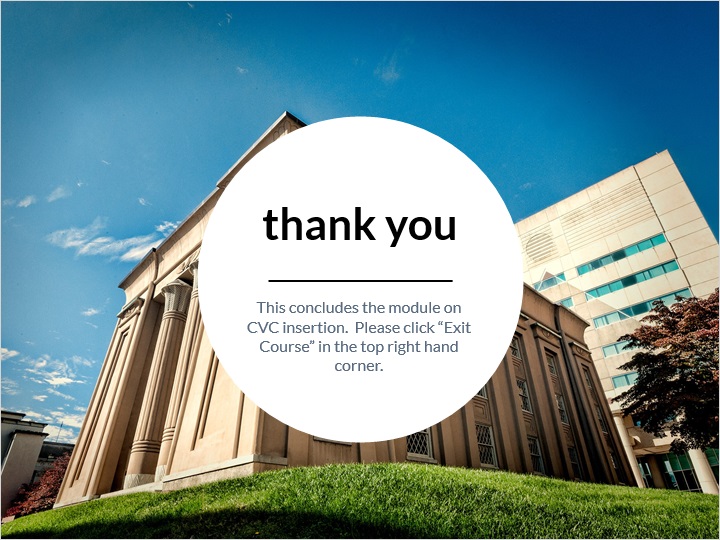

Supplement: Supplementary file 17 — Supplementary file17 (DOC 4.56 MB) [file 11606_2025_9677_MOESM17_ESM.doc]
