# Supplementary material for: Introducing a Comprehensive Framework for Competency-based Procedure Training
Source: J Gen Intern Med. 2025 Jul 8;40(15):3560–5. doi: 10.1007/s11606-025-09677-2 (PMC12612326; doi:10.1007/s11606-025-09677-2)
Supplement: Supplementary file 18 — Supplementary file18 (DOC 2.32 MB) [file 11606_2025_9677_MOESM18_ESM.doc]

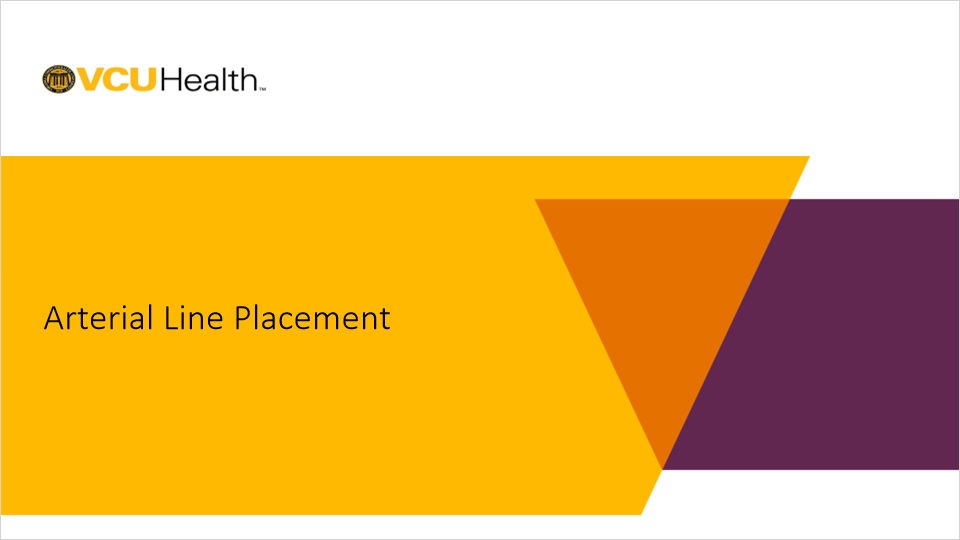


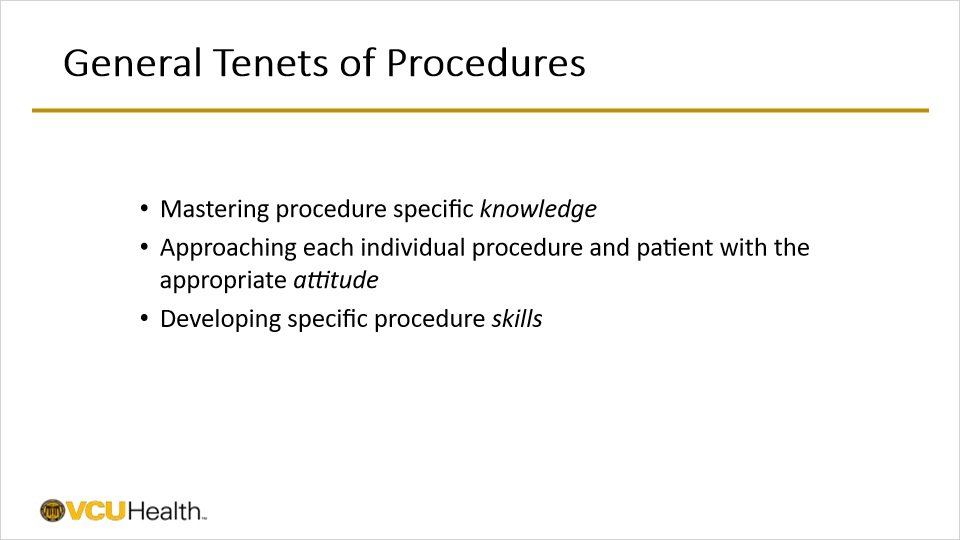


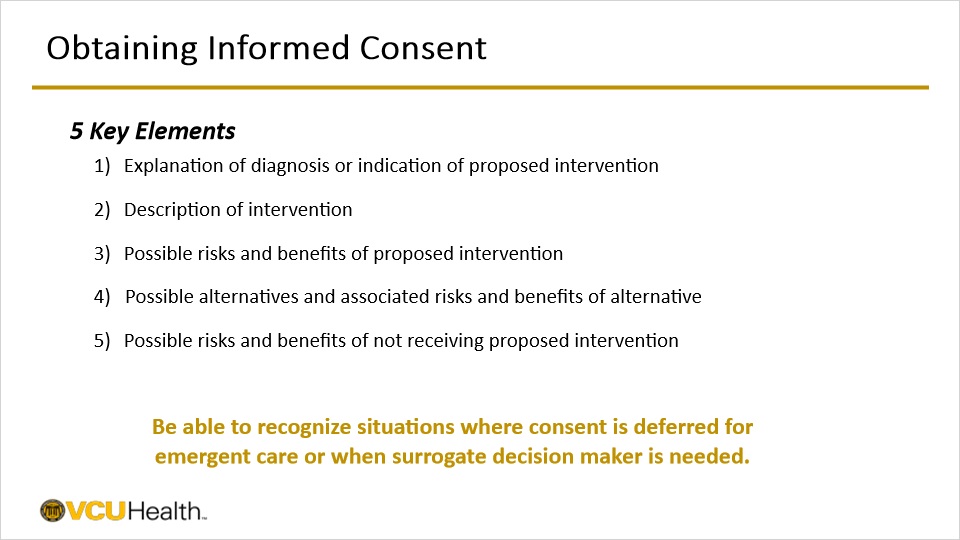


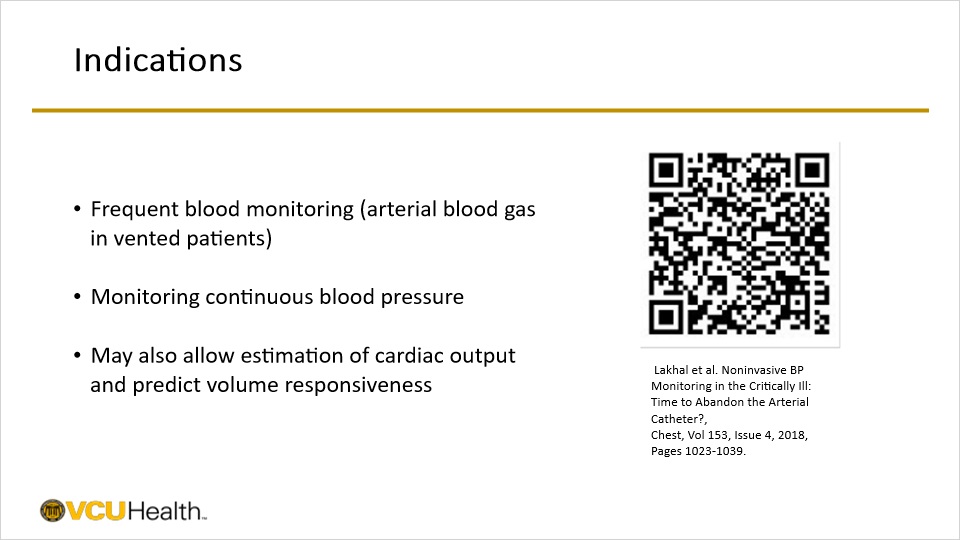


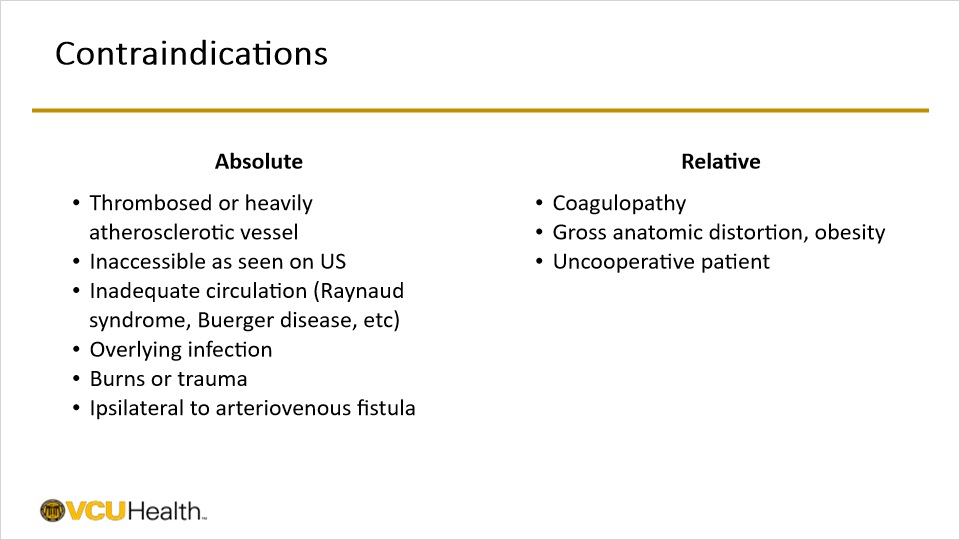


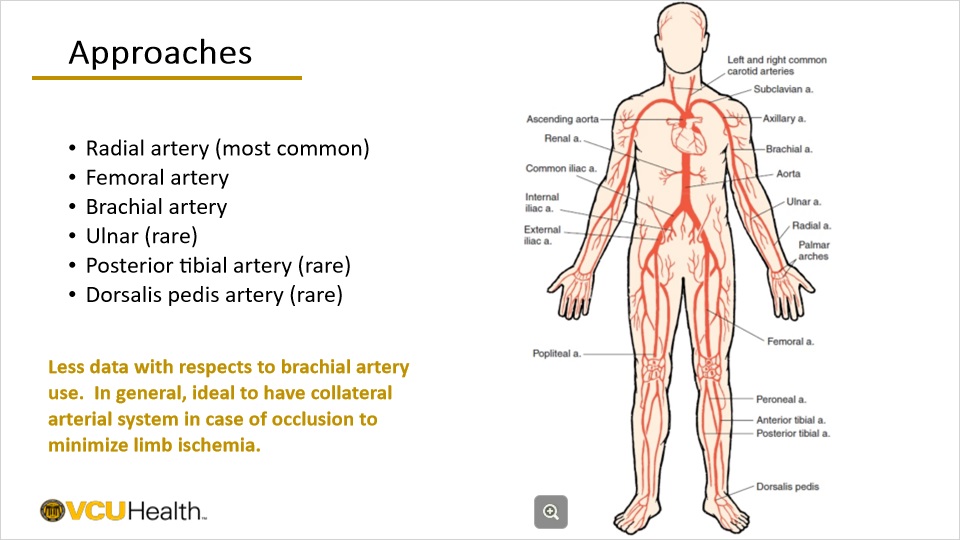


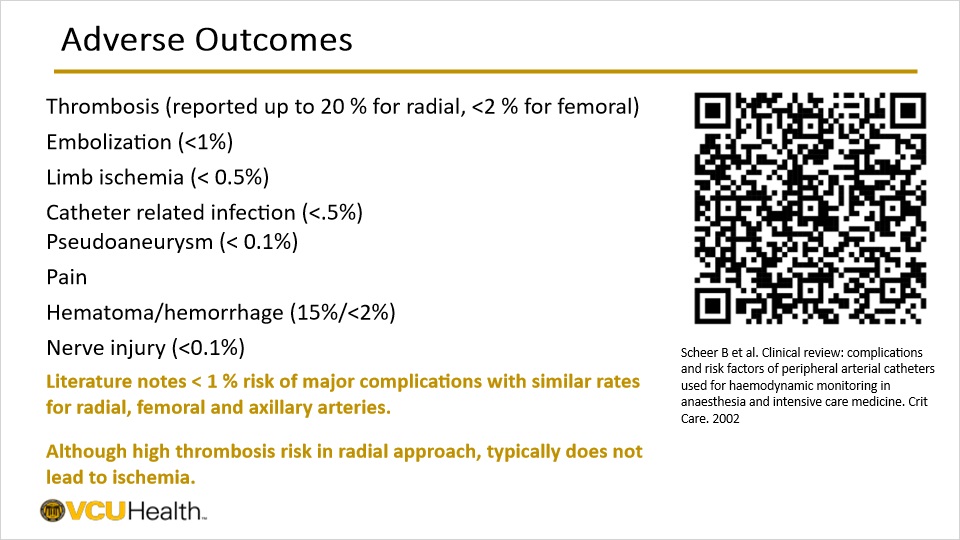


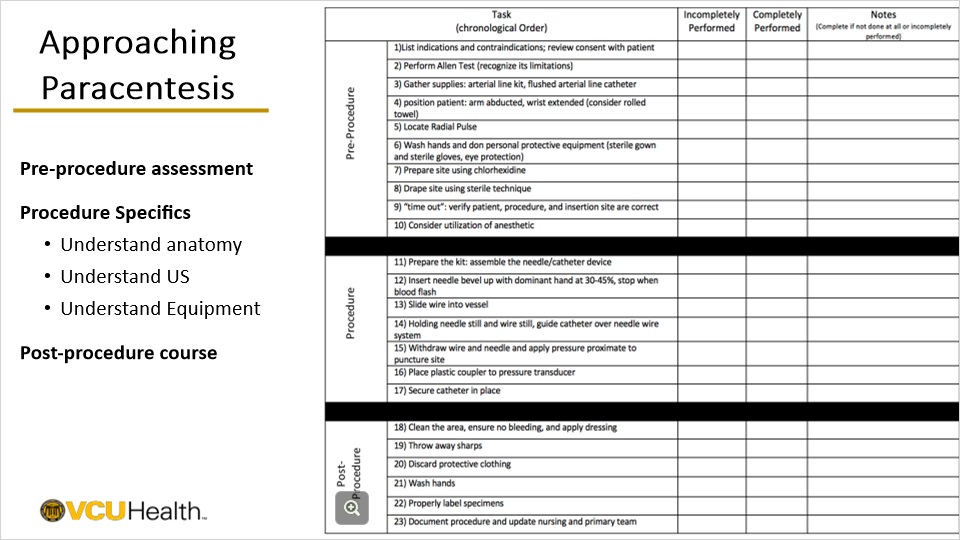


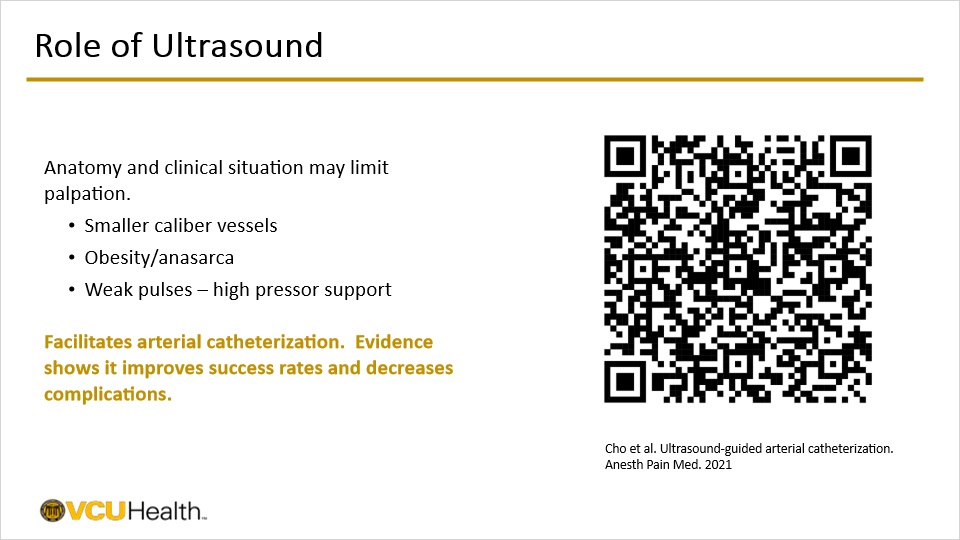


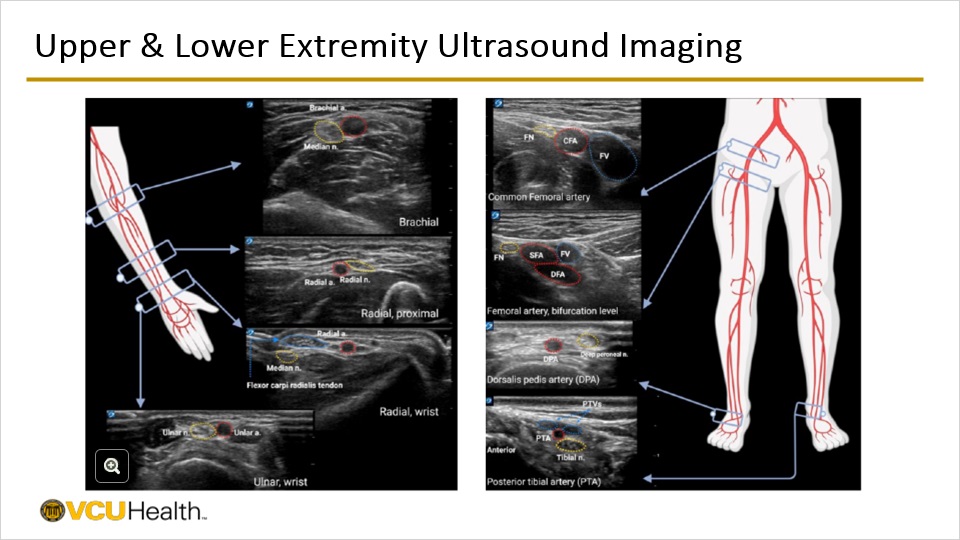


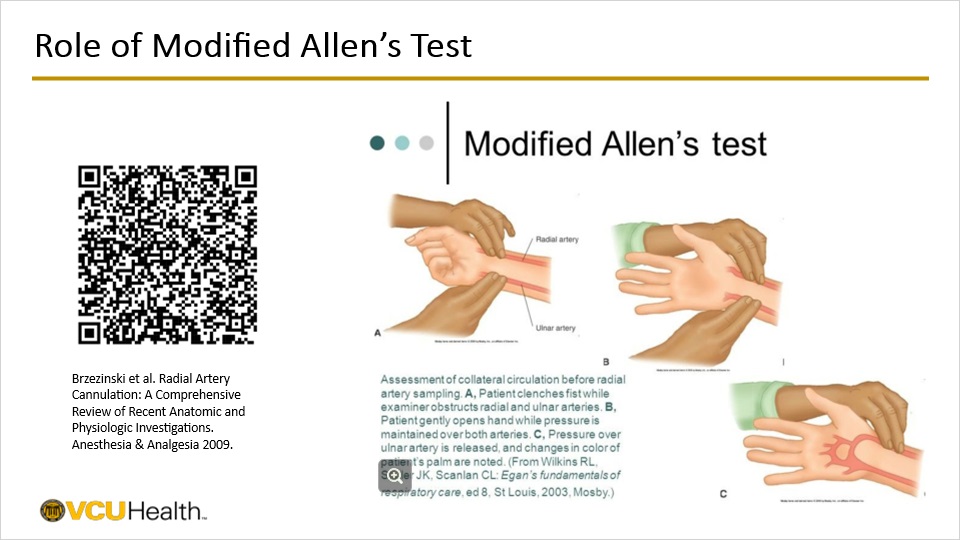


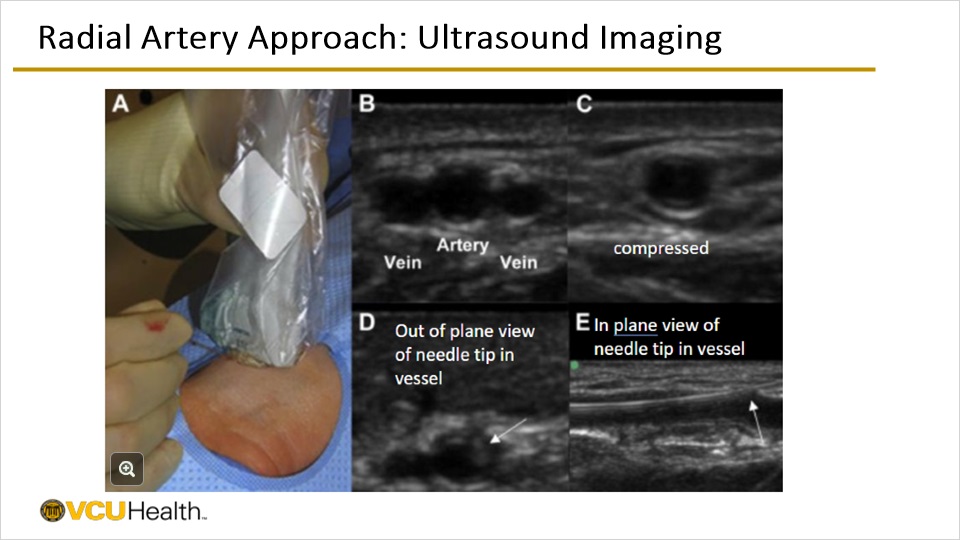


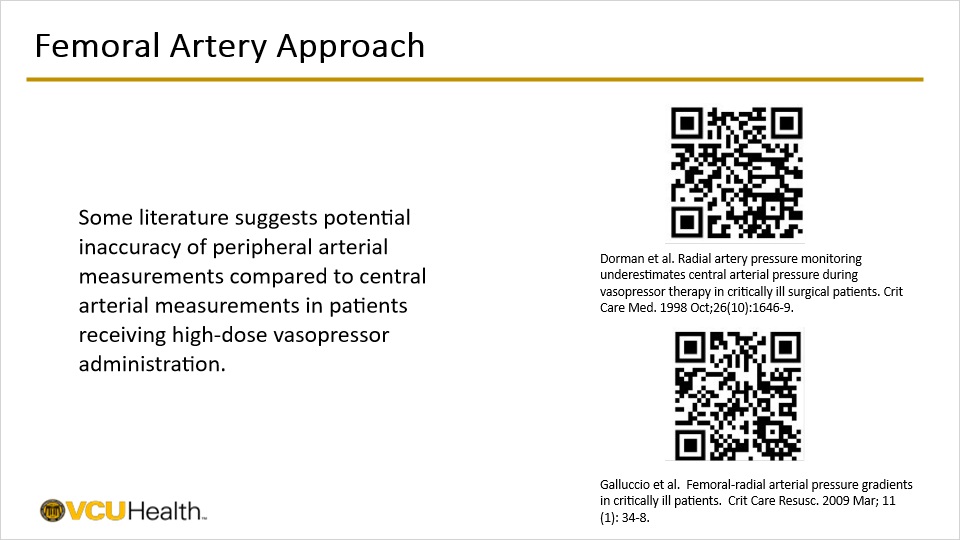


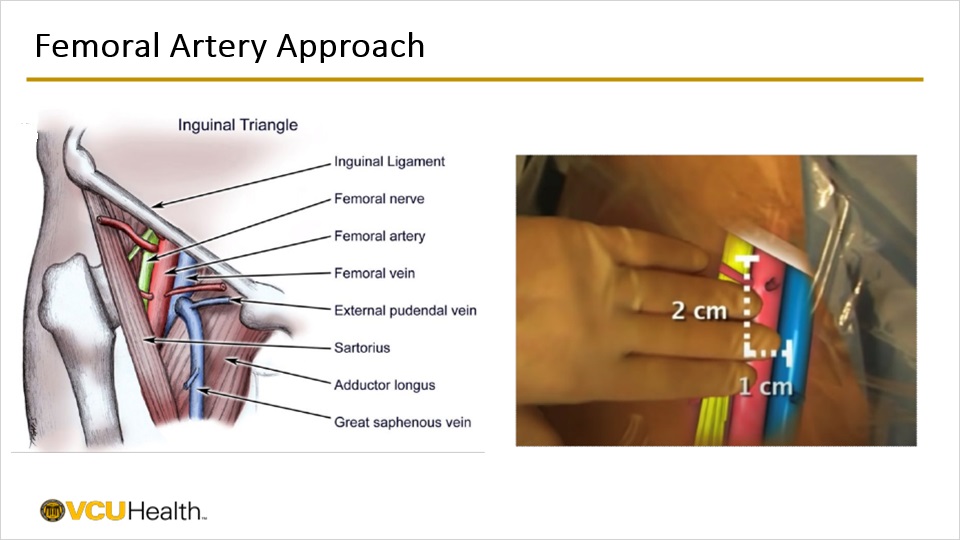


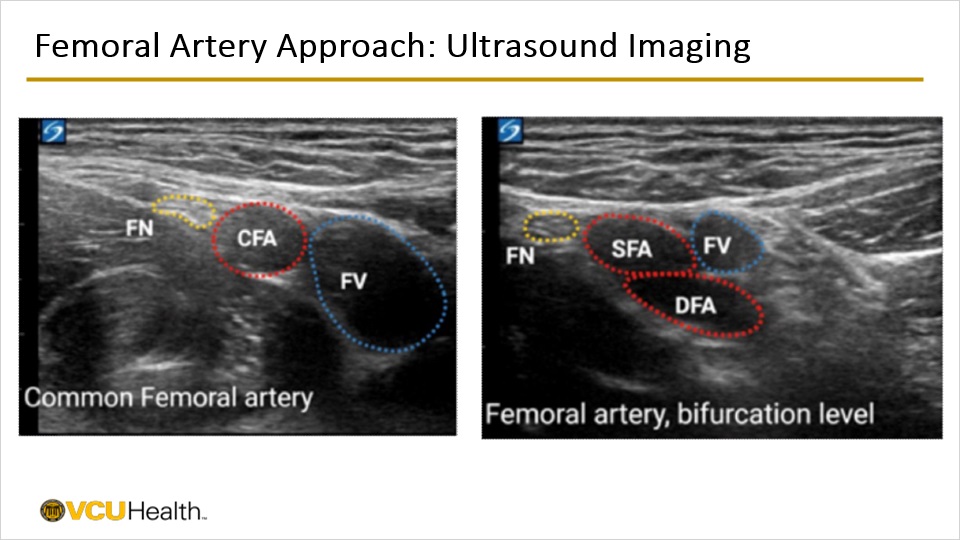


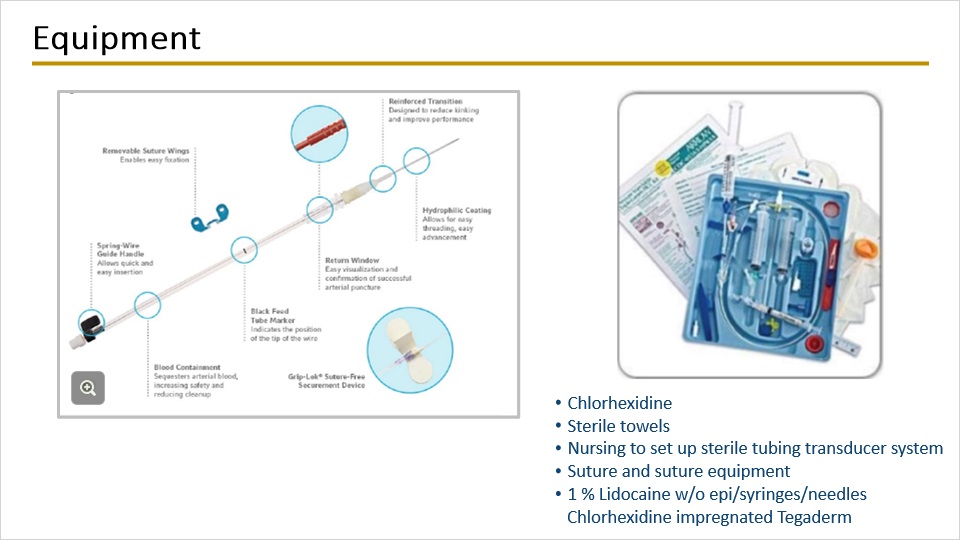


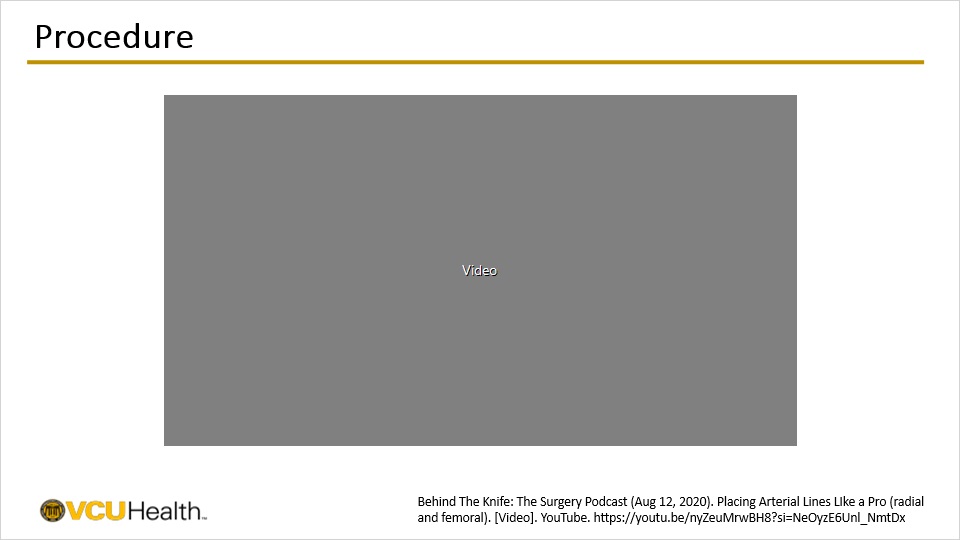


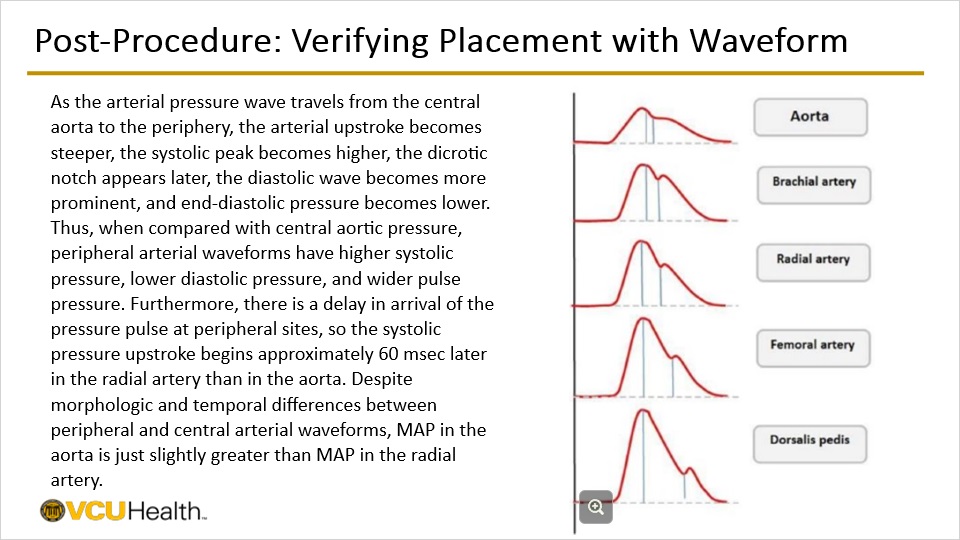


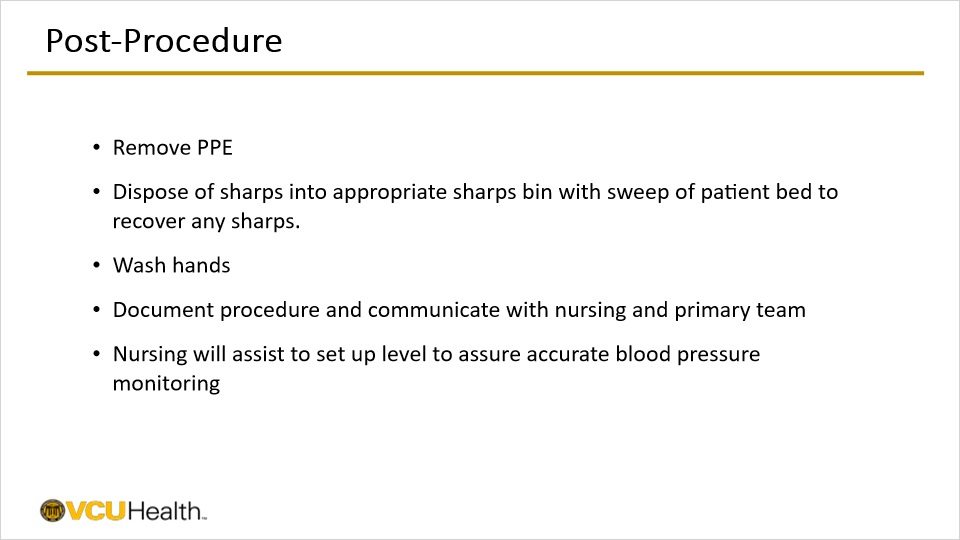


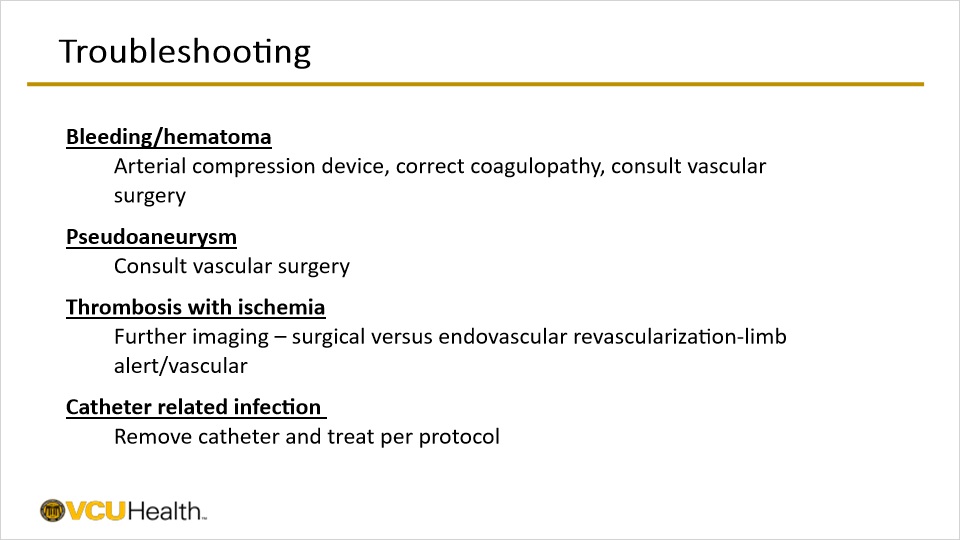


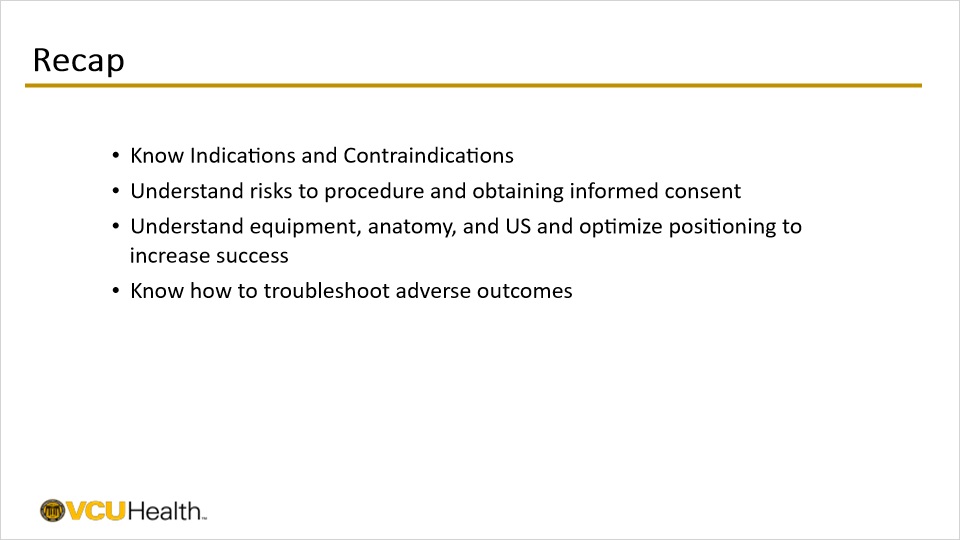


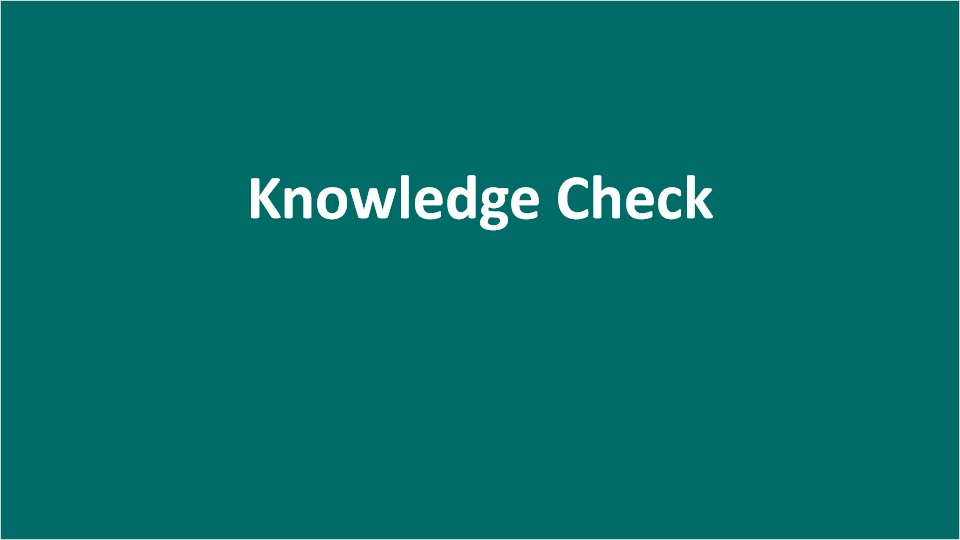


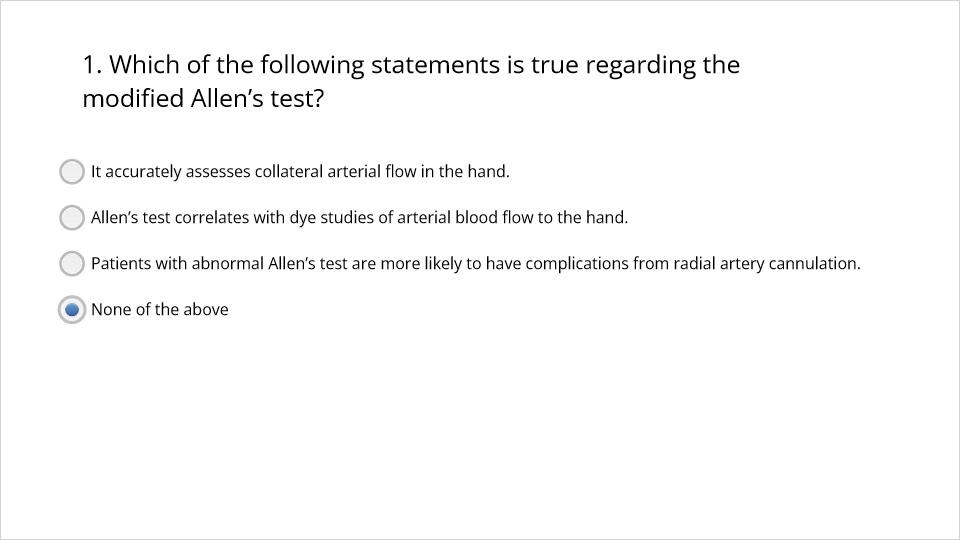


| Correct | Choice |
| --- | --- |
|  | It accurately assesses collateral arterial flow in the hand. |
|  | Allen’s test correlates with dye studies of arterial blood flow to the hand. |
|  | Patients with abnormal Allen’s test are more likely to have complications from radial artery cannulation. |
| X | None of the above |

**Feedback when correct:**

That's right! The modified Allen test showed good reliability as screening tool to avoid ischemia when pursuing radial artery harvest. Has been found comparable to doppler. Major argument against its routine use is the lack of evidence that I can predict hand ischemia after radial artery cannulation. Many studies have noted no issues if abnormal and also ischemia developing in patients with normal testing.

**Feedback when incorrect:**

You did not select the correct response.

| Correct | Choice |
| --- | --- |
|  | Radial artery occlusion is rare after radial artery line placement. |
|  | Ischemic complications usually accompany radial artery occlusion associated with radial artery line placement. |
|  | Infection following arterial line placement is common and requires close attention. |
|  | All of the above |
| X | None of the above |

**Feedback when correct:**

That's right! You selected the correct response. Although thrombosis is reported as high as 20 %, resultant ischemia is noted to be a rare occurrence.

**Feedback when incorrect:**

You did not select the correct response.


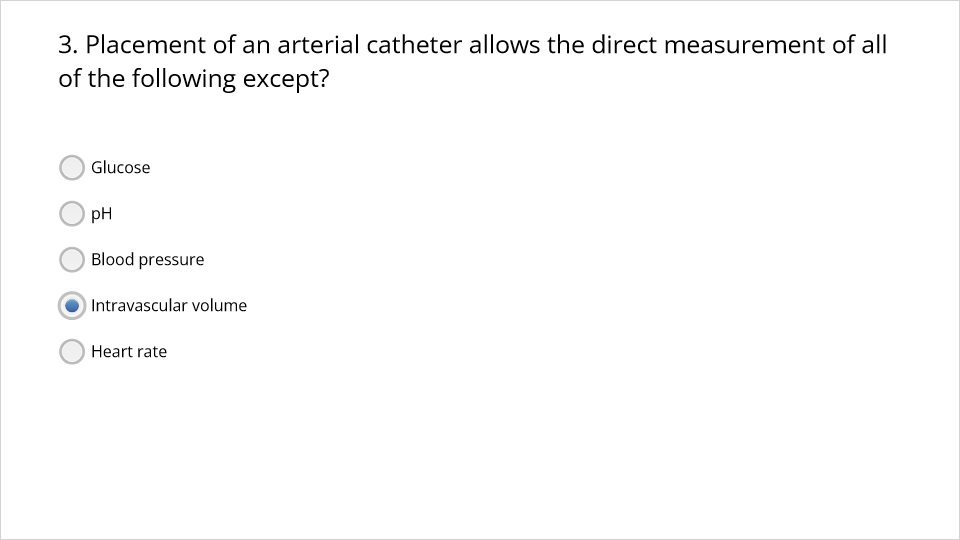


| Correct | Choice |
| --- | --- |
|  | Glucose |
|  | pH |
|  | Blood pressure |
| X | Intravascular volume |
|  | Heart rate |

**Feedback when correct:**

That's right! You selected the correct response. Arterial catheterization allows continuous blood pressure and heart rate monitoring allows routine blood sampling for diagnosis.

**Feedback when incorrect:**

You did not select the correct response.


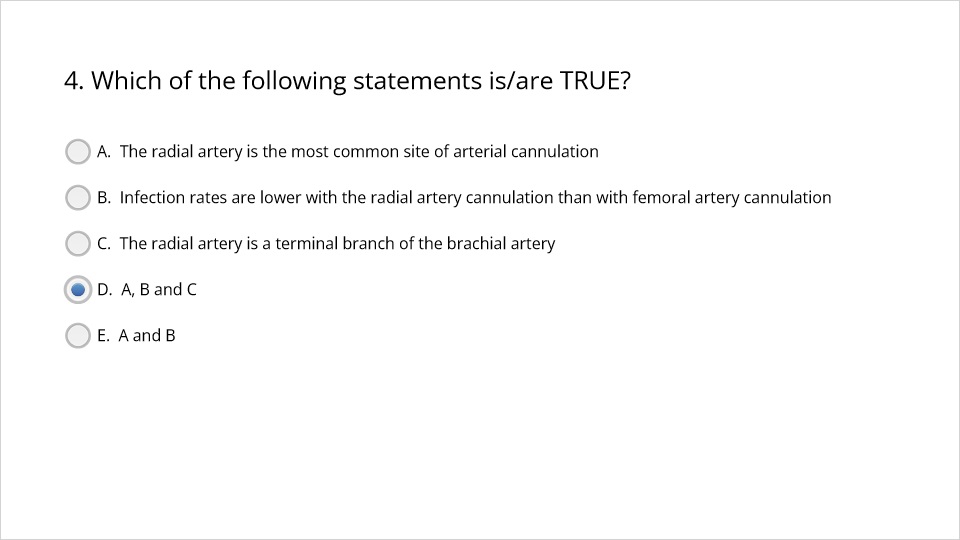


| Correct | Choice |
| --- | --- |
|  | A. The radial artery is the most common site of arterial cannulation |
|  | B. Infection rates are lower with the radial artery cannulation than with femoral artery cannulation |
|  | C. The radial artery is a terminal branch of the brachial artery |
| X | D. A, B and C |
|  | E. A and B |

**Feedback when correct:**

That's right! You selected the correct response. The radial site is the most common site for arterial cannulation and it is a terminal branch of the brachial artery. There is a slightly higher incidence of local infectio0n and sepsis reported with femoral artery access versus radial artery access <1% vs 2 %.

**Feedback when incorrect:**

You did not select the correct response.


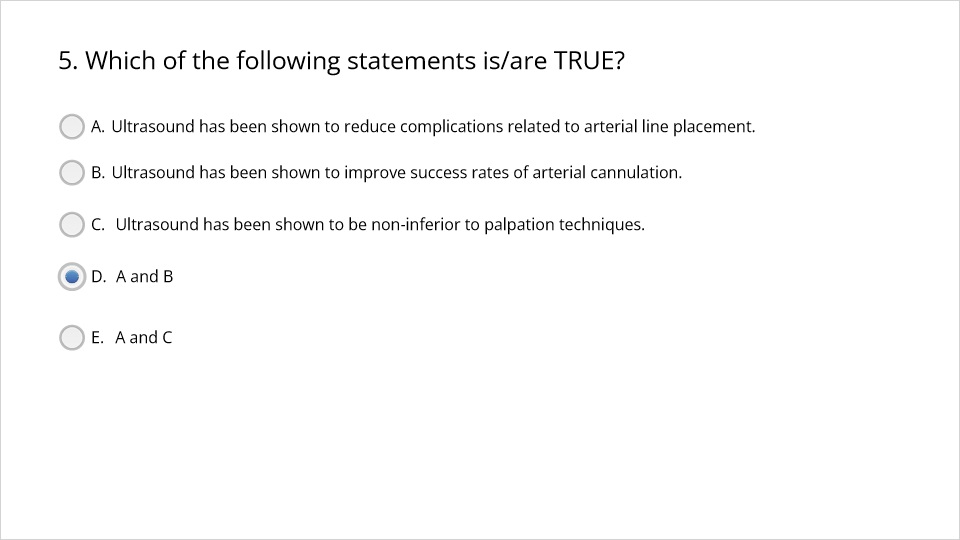


| Correct | Choice |
| --- | --- |
|  | Ultrasound has been shown to reduce complications related to arterial line placement. |
|  | Ultrasound has been shown to improve success rates of arterial cannulation. |
|  | Ultrasound has been shown to be non-inferior to palpation techniques. |
| X | A and B |
|  | A and C |

**Feedback when correct:**

That's right! You selected the correct response. US has been shown to reduce complications related to arterial line placement by improving success rate compared to traditional palpation techniques.

**Feedback when incorrect:**

You did not select the correct response.
