# Supplementary material for: Introducing a Comprehensive Framework for Competency-based Procedure Training
Source: J Gen Intern Med. 2025 Jul 8;40(15):3560–5. doi: 10.1007/s11606-025-09677-2 (PMC12612326; doi:10.1007/s11606-025-09677-2)
Supplement: Supplementary file 19 — Supplementary file19 (DOCX 16.3 KB) [file 11606_2025_9677_MOESM19_ESM.docx]

**Obtaining Consent**

**Pre-requisite Materials:**

1. Review videos on procedure application regarding informed consent and assessing capacity

**Goal**:

Residents should demonstrate the ability to obtain proper informed consent by example of consent for blood product administration in a simulated environment under the guidance of a proctor.

**Objectives**

1. List the 5 key elements of informed consent:
   1. An explanation of the diagnosis or indication of proposed intervention
   2. A description of the intervention
   3. Possible risks and benefits of the proposed intervention
   4. Possible alternative interventions and associated risks and benefits of the alternative
   5. Possible risks of not receiving proposed intervention
2. Identify exceptions to:
   1. Obtaining informed consent:
      1. Emergent situations: immediate intervention required to in order to prevent death or serious health impairment
      2. Extension doctrine: At time of performing a consented to procedure, issues are encountered that require another procedure where obtaining consent would significantly delay patient care or cause harm. (Eg. Placing a chest tube after central line complicated by pneumothorax)
   2. Patient refusal:
      1. When the patient is the only guardian of a minor and it is determined that the well being of the minor supersedes that of the patient
3. Order surrogate decision makers by legal right:
   1. Designee in Advanced Directive
   2. Legally appointed guardian or committee
   3. Spouse (even if estranged, unless divorce is filed but not yet complete)
   4. Adult Child
   5. Parent
   6. Adult Sibling
   7. Any other adult relative
   8. If multiple surrogates from the same hierarchy wish to make a decision, then a majority must agree on a decision (>50%)
4. Deem a patient to have capacity to consent/refuse to an intervention
   1. Identify key elements of capacity
      1. Understanding: Patient able to state meaning of conveyed information (state indication for intervention and risks/benefits/alternatives)
      2. Express a choice: Patient makes a decision, and is consistent with this decision on reassessment (within reason)
      3. Appreciation: Expression of conveyed information applied to the patient
      4. Reasoning: Able to compare choices and delineate possible consequences of choices
   2. Distinguish between competence and capacity
      1. Competence: legal designation, not within scope of resident practice
      2. Capacity: four key elements regarding specific medical decision at specific point in time (eg. Can change as mental status/illness changes)

**Activities**

1. Assigned pre-workshop reading. Assigned pre-workshop knowledge assessment quiz
2. Pre-briefing before workshop reviewing knowledge assessment quiz answers
3. Workshop with opportunity for skill practice in Sim Center with direct coaching and feedback from trained facilitators
4. Post-workshop knowledge assessment quiz
