# Supplementary material for: Introducing a Comprehensive Framework for Competency-based Procedure Training
Source: J Gen Intern Med. 2025 Jul 8;40(15):3560–5. doi: 10.1007/s11606-025-09677-2 (PMC12612326; doi:10.1007/s11606-025-09677-2)
Supplement: Supplementary file 20 — Supplementary file20 (DOCX 17.6 KB) [file 11606_2025_9677_MOESM20_ESM.docx]

**Sterile Technique**

**Pre-requisite Materials:**

1. **NEJM ‘Putting on and Removing Personal Protective Equipment’**
2. (Article) https://www.nejm.org/doi/pdf/10.1056/NEJMvcm1412105?articleTools=true
3. (Video) <https://www.nejm.org/doi/10.1056/NEJMvcm1412105>
4. **Sterile Technique Video**
   1. <https://www.youtube.com/watch?v=Q7t-zgBPQ1M&t=2s>

**Goal**:

Residents should demonstrate the ability, with or without prompting, to successfully place and personal protective equipment (PPE) as well as complete procedural set up using sterile technique in a simulated environment under the guidance of a proctor.

**Objectives**

1. List indications for the use of personal protective equipment and sterile technique as they relate to procedural intervention
   1. Indications for personal protective equipment: Prevent the risk of exposure to infectious material; protect the skin and mucous membranes from exposure to pathogens; identify various precautions including standard, contact, and droplet precautions
   2. Indications for sterile technique: Prevent contamination during invasive procedures; provide personal protection and prevent the risk of exposure to infectious material during procedural intervention
2. Identify and assemble (if indicated) the proper equipment needed for sterile technique
   1. Sterile gloves, boot coverings, sterile gown, face mask (with or without eye shield), eye protection, surgical cap, procedure kit (e.g. arterial line kit), sterile towels/field cover, cleaning solution (e.g. chlorhexidine, iodine)
3. Display the ability to successfully prepare invasive procedure using sterile technique with or without prompting from a proctor
   1. Select appropriate location for kit set up
   2. Practitioner/patient safety
      1. Barrier protection, sterility
   3. Performing adequate sterile technique
      1. Disinfect surface with approved cleaning supplies, prepare procedure kit (if indicated), wash hands, apply mask/shield/hat/boots (if indicated)
      2. Place gloves, gown, and prepare kit using sterile technique with or without assistance from proctor
      3. Open and access procedure kit in sterile fashion
      4. Review procedure preparation including cleaning selected site with chlorhexidine (or other indicated solution)
      5. Drape and cover procedure site to create full sterile field
      6. Successfully remove all equipment and dispose in appropriate fashion
4. Identify common causes of contamination involved with performing sterile technique and review protocol for correction
   1. Identify and remove all equipment in which the integrity of sterilization is compromised, damaged, or suspected
      1. Use sterile barriers and sterile handling to maintain sterility while disposing of contaminated materials
   2. Causes of contamination:
      1. Contact with any non-sterile surface or object
      2. Potential contamination by others (assistant, nurse, tech, etc.)

**Activities**

1. Assigned pre-workshop reading and videos. Assigned pre-workshop knowledge assessment quiz
2. Pre-briefing before workshop reviewing videos and knowledge assessment quiz answers
3. Workshop with opportunity for skill practice in Sim Center with direct coaching and feedback from trained facilitators
4. Log of procedure in New Innovations – co-signature of facilitator
5. Post-workshop knowledge assessment quiz
6. Facilitators or a proxy (APD, Chief Resident) log the procedure assessment (below) in New Innovations
