# Supplementary material for: Introducing a Comprehensive Framework for Competency-based Procedure Training
Source: J Gen Intern Med. 2025 Jul 8;40(15):3560–5. doi: 10.1007/s11606-025-09677-2 (PMC12612326; doi:10.1007/s11606-025-09677-2)
Supplement: Supplementary file 21 — Supplementary file21 (DOCX 18.2 KB) [file 11606_2025_9677_MOESM21_ESM.docx]

**VENIPUNCTURE AND PERIPHERAL IV CANNULATION**

Performance Checklist for Intern Orientation

**Name: ­­­­­ Date**:

**Proctor:**

| **Task** | | **Incompletely**  **Performed**  **(1 point)** | **Completely**  **Performed**  **(2 points)** | **Notes**  (Complete this section if learner does not complete tasks or incompletely performs) |
| --- | --- | --- | --- | --- |
| **Pre-Procedure** | 1. Lists indications and contraindications; review procedure and risks with patient |  |  |  |
|  | 1. Gather supplies: IV start kit, needle, tourniquet, gauze, occlusive dressing, collection tubes), gloves/goggles, antiseptic applicator/swab, saline flush, ultrasound with linear probe, probe cover |  |  |  |
|  | 1. Position Patient: Preferred veins are upper extremities; patient supine |  |  |  |
|  | 1. Wash hands and don personal protective equipment (nonsterile gloves, goggles) |  |  |  |
|  | | | | |
| **Procedure** | 1. Tie tourniquet 3-4 inches above site |  |  |  |
|  | 1. Inspect and palpate available veins |  |  |  |
|  | 1. Sterilize skin above the selected vein |  |  |  |
|  | 1. With dominant hand, insert needle bevel up at 5-30º angle, +/- US guidance |  |  |  |
|  | 1. Stop when flash of blood seen; lower needle to skin, and advance catheter with stabilized needle |  |  |  |
|  | 1. Remove tourniquet |  |  |  |
|  | 1. Remove needle and place in sharps box |  |  |  |
|  | 1. Flush cannula with normal saline |  |  |  |
|  | | | | |
| **Post-procedure** | 1. Inspect for signs of extravasation |  |  |  |
|  | 1. Attach stat lock and disinfecting cap |  |  |  |
|  | 1. Apply occlusive dressing, and secure tubing with tape |  |  |  |
|  | 1. Remove protective equipment; wash hands |  |  |  |
