# Supplementary material for: Introducing a Comprehensive Framework for Competency-based Procedure Training
Source: J Gen Intern Med. 2025 Jul 8;40(15):3560–5. doi: 10.1007/s11606-025-09677-2 (PMC12612326; doi:10.1007/s11606-025-09677-2)
Supplement: Supplementary file 23 — Supplementary file23 (DOCX 14.6 KB) [file 11606_2025_9677_MOESM23_ESM.docx]

*Longitudinal Curriculum Requirements*

**Endocrine/Allergy/Immunology:**

1. Basic procedure minimums (one ABG/PIV/Venipuncture)
2. Resident should participate in *3 paracenteses, 3 lumbar punctures, 3 CVC*

**Primary care/Rheumatology**

1. Basic procedure minimums as stated above
2. Resident should participate in 3 paracenteses, 3 lumbar punctures, 3 cvc, and *3 arthrocenteses*

**Nephrology**

1. Basic procedure minimums as stated above
2. Resident should participate in 3 paracentesis, 3 lumbar puncture, *10 CVC*
3. *Resident should try and achieve indirect supervision level for CVC by time of graduation if they have interest in procedures*

**Infectious disease/Hematology/Oncology**

1. Basic procedure minimums as stated above
2. Resident should participate in 3 paracentesis, *5 lumbar puncture*, 3 CVC
3. *Resident should try and achieve indirect supervision level for lumbar puncture prior to graduation*

**Gastroenterology**

1. Basic procedure minimums as stated above
2. Resident should participate in *10 paracentesis*, 3 lumbar puncture, 3 CVC
3. *Resident should try and achieve indirect supervision level for paracentesis by time of graduation*

**Cardiology**

1. Basic procedure minimums as stated above
2. Resident should participate in *10 arterial lines, 10 CVC,* 3 lumbar punctures, 3 paracenteses
3. *Resident should try and achieve indirect supervision level for CVC and art line insertion by time of graduation*

**Pulmonology Critical Care/Hospital Medicine***

1. Basic procedure minimums as stated above
2. Resident should participate in *10 CVC, 10 arterial lines, 10 paracentesis, 5 lumbar punctures, 3 thoracentesis, 3 arthrocentesis*
3. *Resident should try and achieve indirect supervision level for all procedures by time of graduation*

*Recommendations for hospitalists who desire to pursue procedures in their career
