# Supplementary material for: Introducing a Comprehensive Framework for Competency-based Procedure Training
Source: J Gen Intern Med. 2025 Jul 8;40(15):3560–5. doi: 10.1007/s11606-025-09677-2 (PMC12612326; doi:10.1007/s11606-025-09677-2)
Supplement: Supplementary file 24 — Supplementary file24 (DOCX 16.3 KB) [file 11606_2025_9677_MOESM24_ESM.docx]

**Graduation Procedure Competency Form**

| **Section II:  Procedures** | |
| --- | --- |
| **In our training program, all paracenteses and lumbar punctures are performed utilizing static ultrasound  for site identification and all central venous catheter insertions are performed with dynamic ultrasound guidance. We do NOT provide training or certification in point of care ultrasound and scope of ultrasound use is limited to the above.  All residents have demonstrated effectiveness in obtaining consent, utilizing standard universal precautions, establishing a sterile field, and applying local anesthetic.**  **Our procedural competency committee meets twice annually to review loggers, evaluations, and self-assessments to better assess resident performance.  Residents advance in the spectrum below when all end goals are achieved in each of the three categories. (see appendix)**  **We are unable to comment on requested clinical privileges/procedures outside the scope of a general internal medicine residency training program.  At the conclusion of this trainee’s Internal Medicine residency training, they were judged capable of performing the following procedures independently.** | |
| **☐ Abdominal paracentesis**  **☐ Arterial line insertion**  **☐ Lumbar Puncture**  **☐ Insertion of femoral central line**  **☐ Other  __ ______**  *** *If “Other” is selected, an explanation may be provided in the “Additional Comments” section below or in an enclosed separate document.*** | **☐ Insertion of internal jugular central line**  **☐ Insertion of subclavian central line**  **☐ Arthrocentesis of the knee**  **☐ Thoracentesis** |
| \| **Procedure** \| **Total Number** \| **S+A** \| **DS** \| **IND** \| **IND + SO** \| \| --- \| --- \| --- \| --- \| --- \| --- \| \| **Central Venous Access**   1. **Internal jugular approach** 2. **Femoral line Approach** 3. **Subclavian Line Approach** \|  \|  \|  \|  \|  \| \| **Paracentesis** \|  \|  \|  \|  \|  \| \| **Arthrocentesis** \|  \|  \|  \|  \|  \| \| **Lumbar Puncture** \|  \|  \|  \|  \|  \| \| **Thoracentesis** \|  \|  \|  \|  \|  \| \| **Arterial line placement** \|  \|  \|  \|  \|  \|   **S+A = supervision and assistance, DS = direct supervision, IND = independent, IND + SO = independent + supervision of others** | |
