# Supplementary material for: Introducing a Comprehensive Framework for Competency-based Procedure Training
Source: J Gen Intern Med. 2025 Jul 8;40(15):3560–5. doi: 10.1007/s11606-025-09677-2 (PMC12612326; doi:10.1007/s11606-025-09677-2)
Supplement: Supplementary file 27 — Supplementary file27 (DOCX 40.0 KB) [file 11606_2025_9677_MOESM27_ESM.docx]

**Educational Goals**

The purpose of the longitudinal curriculum in procedure medicine for Internal Medicine residents is provide trainees with ongoing opportunity to perform procedures with enhanced quality and safety via standardized training and with adequate supervision.

This curriculum ensures that residents not only meet the [minimum requirements](https://www.abim.org/~/media/ABIM%20Public/Files/pdf/publications/certification-guides/policies-and-procedures.pdf) to be eligible for certification by the American Board of Internal Medicine (ABIM), but have a clear understanding of the standards that must be met to be deemed competent to perform procedures independently and have an opportunity to meet those standards if so desired by the trainee.

By the end of training, residents will meet the minimum residency program requirements for procedures training and have a designated level of competency. for individual procedures that are taught but in which independence is not required to graduate (paracentesis, central venous catheterizations, and lumbar punctures).

**Curriculum Components**

Specific learning objectives are outlined within each component of the curriculum

**PGY1:**

1. Procedure Longitudinal Curriculum Overview: Orientation Week
2. Orientation Procedure Training: Masterly learning simulation experience for peripheral IV insertion, venous blood draw, arterial blood gas/arterial line placement, sterile technique, obtaining informed consent
3. Vascular Access Training Session: Pre-ICU mastery learning simulated experience of US guided CVC and arterial line insertion
   - Mandatory learning exchange module and post-test prior to attending session
4. Supervised practice in clinical environment

**PGY2:**

1. Summer Series Mastery Learning Simulation
   1. Mandatory learning exchange modules and post-test prior to attending sessions
   2. Log simulated assessments after having obtained minimum passing standard in in simulated environment – log to Pete Meliagros
2. [Procedure Rotation](file:///Users/petemeliagros/Downloads/Procedure%20Med%20Curriculum%20v1.0.docx)
   1. Sim lab skills sessions
   2. Supervised practice in clinical environment
3. ICU Rotations/Night Float
   1. Supervised practice in clinical environment

**PGY3:**

- Advanced tailored procedure elective if on procedure track, on request or if deemed necessary by procedure competency committee (all residents pursuing cardiology, PCCM, and hospital medicine with procedure interests/career aspirations in procedures will rotate through advanced procedure elective)
  1. Residents pursuing PCCM or hospital medicine with interest in procedures will have two half days/week to rotate with IP team on procedure team starting October 1^st^.
  2. Residents pursing cardiology will be individually scheduled in cath lab once or twice weekly to gain vascular access and participate in right heart catheterizations

1. Clinical rotations

**Instructional Activities/Assignments and Associated Resources**

The following resources (which can be found on the procedure app under resources) should be used to supplement knowledge in topics not covered through clinical experience and dedicated educational sessions:

- Sentinel Articles
- Learning Modules
- Videos
- Checklists
- Etc.

**Feedback from Learners/Rotation Evaluation**

- Learners will give feedback via Rotation Evaluation following the Procedure Medicine Rotation
- Learners will give feedback as needed to program leadership and via the Residency Advisory Committee or the Curriculum Committee
- Learners will have the opportunity to give feedback and express concerns on the Annual Program Survey

**Evaluation of Learner**

**Summary of Assessments** (also to be outlined in curricula of individual components)

PGY1

- Log intern orientation procedures/skills: PIVs, venous blood draw, ABGs,
- Attend vascular access course and log assessment for CVC and art line insertion to Pete Meliagros
- Completion of vascular access workday modules (art line insertion and CVC) with post tests
- Log all procedures
- Procedure Competency Committee will evaluate procedure portfolio and provide global report with comments twice annually. Residents will receive an email with this information from the Associate Program Director of Procedure Medicine

PGY2

- Complete workday modules (CVC, LP, Para) and post-tests prior to attending summer series training sessions
- Log attendance at Summer Series sim lab sessions
- Upon obtaining minimum passing score (as deemed by PCC) - assure log simulated assessment and submit to Pete Meliagros
- Simulation training refresher at start of procedure medicine rotation
- Assure receiving feedback and logging all procedures attempted on patients (procedure rotation AND outside of rotation) – if issues finding supervisor – send to APD for Procedure Medicine
- Procedure medicine rotation evaluation, with summative evaluation of [competency level](file:///Users/petemeliagros/Downloads/Procedure%20Competency%20Levels%20description.docx) for each procedure
- Procedure Competency Committee will evaluate procedure portfolio and provide global report with comments twice annually. Residents will receive an email with this information from the Associate Program Director of Procedure Medicine

PGY3

- Procedure Competency Committee will evaluate procedure portfolio and provide global report with comments twice annually. Residents will receive an email with this information from the Associate Program Director of Procedure Medicine.
- All graduates will receive personalized detailed committee procedure review of competency in each procedure
- PCC also will review for progression of competency upon request of trainee
- Residents to assure have participated in minimum number of procedures for their selected track prior to graduation.

**Safety and Supervision Policies**

1. All Internal Medicine residents (PGY1 and up) are required to have direct supervision by an attending or fellow who has undergone approved, standardized training regardless of competency level achieved.
2. “Key Elements” of a procedure are those that have significant safety implication and include needle/catheter insertion (excluding small needle for lidocaine injection), guidewire insertion/removal, and dilation.
3. Any Internal Medicine resident (PGY1 and up) must undergo simulation-based training and be deemed competent to perform key elements of a procedure in the simulated environment prior to attempting on a patient in the clinical environment. (residents who have completed and can verify similar training in medical school can reach out to Procedure Medicine APD to set up simulated experience with run through of checklist with minimum passing score to be able to conduct key components)
   1. By the curriculum levels of competency, the trainee would be designated as “able to perform with supervision and assistance” for this procedure.
   2. If a resident has not been deemed competent in the simulation environment but has undergone the components of the PGY1 curriculum, she or he may assist with non-key elements of the procedure (i.e. preparation of patient and equipment, superficial lidocaine injection, subsequent steps following key elements).
      1. By virtue of the curriculum, generally no PGY1 residents should be able to attempt key elements of a lumbar puncture and paracentesis. They should have special permission to do so by the Procedural Competency Committee.
      2. By virtue of the curriculum, almost all PGY2 residents should be able to perform these procedures with supervision and assistance starting in Block 3 (when they have completed the Summer Series simulation training).
      3. Refer to the competency levels for more information.
      4. By virtue of the current curriculum, arterial lines and thoracentesis should always be performed with supervision and ability to perform key component can be at the discretion of the supervising provider.

**Role of the Procedure Competency Committee**

- Biannual review of data from trainees and recommend the following:
  - Progress with completion of program procedure requirements
  - Level of competency for individual procedures
- On demand evaluation of level of competency as requested by trainees
  - If they wish to progress to “able to perform with supervision and assistance” prior to PGY2 summer series
  - If they wish to progress to “able to perform with supervision” prior to PGY2 Procedure Medicine Rotation
  - If they wish to progress beyond level determined at conclusion of PGY2 Procedure Medicine Rotation

**Medicine Residency Training Program Procedure Requirements**

1. By the end of residency, **all** VCU IM residents are required to serve as the primary operator in a minimum of these basic procedures:
   1. 1 venous blood draws
   2. 1 arterial blood draws (ABGs)
   3. 1 peripheral venous line placements
2. In order to graduate well rounded physicians and to also better prepare residents pursuing specialty fields that have more of a focus on procedures, the program is pursuing more specific graduation goals for residents for more advanced bedside procedures (if undetermined track or determined later in training, default will be for lowest requirements):
   1. **Endocrine/Allergy/Immunology:**
      1. Basic procedure minimums as stated above
      2. Resident should participate in *3 paracenteses, 3 lumbar punctures, 3 CVC*
   2. **Primary care/Rheumatology**
      1. Basic procedure minimums as stated above
      2. 3 paracentesis, 3 lumbar punctures, 3 cvc, and ***5*** *knee arthrocenteses*
   3. **Nephrology**
      1. Basic procedure minimums as stated above
      2. Resident should participate in 3 paracentesis, 3 lumbar puncture, ***5*** *CVC*
      3. Resident should try and achieve indirect supervision level for *CVC* by time of graduation
   4. **Infectious disease/Hematology/Oncology**
      1. Basic procedure minimums as stated above
      2. 3 paracentesis, ***5*** *lumbar puncture*, 3 cvc
      3. Resident should try and achieve indirect supervision level for *lumbar puncture* prior to graduation
   5. **Gastroenterology**
      1. Basic procedure minimums as stated above
      2. Resident should participate in ***10*** *paracentesis*, 3 lumbar puncture, 3 CVC
      3. Resident should try and achieve indirect supervision level for *paracentesis* by time of graduation
   6. **Cardiology/Pulmonology/Critical Care/Hospital Medicine***
      1. Basic procedure minimums as stated above
      2. Resident should participate in ***10*** *paracenteses,* ***10*** *arterial lines,* ***10*** *CVC,* ***5*** *lumbar punctures,* ***3 thoracentesis***
      3. Resident should try and achieve indirect supervision level for all procedures by time of graduation

*Hospitalists who have interest in conducting procedures in their career. Those who do not have an interest in procedures can default to participating in 3 para, 3 LP, 3 CVC

For all procedures, meeting the minimum number completed indicates that the resident has had adequate experience to obtain sufficient knowledge, attitudes, and basic skills to be board certified in Internal Medicine. Per ABIM requirements for certification, residents must:

1. Demonstrate competence in medical knowledge relevant to procedures through their ability to explain indications, contraindications, patient preparation methods, sterile techniques, pain management, proper techniques for handling specimens and fluids obtained, and test results
2. Be able to recognize and manage complications
3. Clearly explain to a patient all facets of the procedure necessary to obtain informed consent.

To help acquire both knowledge and performance competence, ABIM believes that residents should be active participants in performing procedures. Active participation is defined as serving as the primary operator or assisting another primary operator. ABIM encourages program directors to provide each resident with sufficient opportunity to be observed as an active participant in the performance of required procedures. ABIM does not specify a minimum number of procedures to demonstrate competency; however, to assure adequate knowledge and understanding of the common procedures in internal medicine, each resident should be an active participant for each procedure three or more times.

Completion of the minimum number required does NOT indicate the resident is competent to perform these procedures independently. Ability to perform procedures without direct supervision will be determined by trained supervisors who directly supervise and give feedback to the resident on those procedures.

At any given time, a resident will be rated at a certain level of competence for each procedure:

- 1. Able to observe
  2. Able to participate (non-key elements)
  3. Able to perform with supervision and assistance (attempt key elements of procedure)
  4. Able to perform with supervision
  5. Able to perform independently (with indirect supervision)
  6. Able to perform independently and supervise others

For advanced procedures, a level of able to perform with supervision and assistance is considered Active Participation (attempt serving as primary operator but may require assistance). Thus, residents must perform a minimum of 3 of each procedure with supervision and assistance to meet requirements for completion of training.

These requirements are in accordance with [ABIM Policies and Procedures for Certification](https://www.abim.org/certification/policies/internal-medicine-subspecialty-policies/internal-medicine/), including requirements for competency and educational experience. Procedures not listed above are those in which residents get adequate participation via the clinical rotation and other program
